# Conjugation of chemical handles and functional moieties to DNA during solid phase synthesis with sulfonyl azides

**DOI:** 10.1093/nar/gkac566

**Published:** 2022-07-08

**Authors:** Angel Santorelli, Kurt V Gothelf

**Affiliations:** Department of Chemistry and Interdisciplinary Nanoscience Centre (iNANO), Aarhus University, Aarhus, Central Denmark Region, 8000, Denmark; Department of Chemistry and Interdisciplinary Nanoscience Centre (iNANO), Aarhus University, Aarhus, Central Denmark Region, 8000, Denmark

## Abstract

Labelling of oligonucleotides with dyes, targeting ligands, and other moieties has become ever more essential in life-sciences. Conventionally, modifications are introduced to oligonucleotides during solid phase synthesis by special phosphoramidites functionalised with a chemical handle or the desired functional group. In this work, we present a facile and inexpensive method to introduce modifications to oligonucleotides without the need for special phosphoramidites. Sulfonyl azides are applied to react with one or more selected phosphite intermediates during solid phase synthesis. We have prepared 11 sulfonyl azides with different chemical handles such as amine, azide, alkyne, and thiol, and we have further introduced functionalities such as pyrene, other dyes, photo-switchable azobenzenes, and a steroid. The method is compatible with current phosphoramidite-based automated oligonucleotide synthesis and serves as a simple alternative to the unstable and expensive special phosphoramidites currently used for conjugation to oligonucleotides.

## INTRODUCTION

In 1981, Matteucci and Caruthers first reported on solid phase DNA synthesis using phosphoramidite chemistry (1), thus making it feasible to synthesise single-stranded DNA chemically. Since then, the technology has advanced and been greatly optimised, while the underlying chemistry is virtually unchanged (2–4). Nowadays, oligonucleotide synthesis has become essential in life-sciences. A large share of synthetic oligonucleotides is used as PCR primers in diagnostics, in sequencing, and in gene synthesis for synthetic biology. However, oligonucleotide therapies such as siRNA, antisense oligonucleotides, and CRISPR-Cas9 is a growing area within an increasing market (5–9).

Solid phase chemical synthesis has allowed the creation of highly customised oligonucleotides. The capability of changing reagents in any of the synthesis cycles allows for the introduction of a myriad of functional groups and structural changes, e.g. changing the sugar in the backbone and introducing non-canonical nucleobases. In particular, modification of the sugar, as in 2-flouro, 2-methoxyethyl, LNA and other nucleic acid analogues are widely used in nucleic acid therapeutics (10,11). In some cases, the internucleotide phosphodiester linkage is also modified (4). Another major area is the introduction of various functional chemical groups such as dyes or chemical handles for post-synthesis modification of the oligonucleotides.

Most chemical modifications of oligonucleotides with custom functional groups of interest are introduced via phosphoramidite building blocks. Commercial catalogues of custom phosphoramidites exceed several hundred variations. However, such custom phosphoramidites are in most cases much more expensive than the canonical nucleoside phosphoramidites. Furthermore, phosphoramidites are prone to hydrolysis and oxidation, and must be stored at −20°C under inert atmosphere. When applied to solid phase chemical synthesis these phosphoramidites are used in solution at room temperature where they degrade over time (12,13).

The internucleotide phosphate linker is an interesting site for chemical functionalisation of nucleic acids. The nucleophilic and redox properties of the phosphite intermediate formed in the synthesis cycle opens the door to the development of new methods to introduce different chemical groups. The classical modification is to oxidise intermediate phosphites to the corresponding phosphorothioates. This is largely used to increase the properties of antisense and siRNA oligonucleotides (4,14). Further efforts have been devoted to develop new modifications for the internucleotidic link. An interesting and emerging method is the application of sulfonyl azides to convert the intermediate phosphites to sulfonylphosphoramidates. In 1988, Nielsen and Carruthers reported on the first reactions of azides with an internucleoside phosphite triester in solution (15). The application of the method to solid phase synthesis of oligonucleotides was described in the patent literature 15 years ago (16). In recent years, Stetsenko *et al.*, and a few other groups have explored sulfonyl azides as a method to introduce multiple modifications of small hydrophobic groups in the same oligonucleotide to tune the metabolic stability and viability of the modified oligonucleotides for potential therapeutic applications (17–22). The sulfonylphosphoramidates at which the negative charge is conserved are very stable; furthermore, they have relatively small impact on the melting temperature of duplexes with DNA and RNA (23–25).

While sulfonyl azides have been used to modify the properties of oligonucleotides, they also offer an attractive opportunity to functionalise oligonucleotides with chemical handles, dyes etc. Compared to phosphoramidite-based modifiers, the use of sulfonyl azides offers several potential advantages. They are easy to synthesise, air and moisture stable, and their incorporation is not limited to terminal positions. In addition, the formation of sulfonylphosphoramidates does not compromise the sequence of the oligonucleotide, nor do they introduce gaps and nicks in the backbone. In this work, we demonstrate a versatile and straightforward protocol to modify oligonucleotides with a variety of different functional groups of interest using sulfonyl azides applied in solid phase oligonucleotide synthesis (SPOS).

## MATERIALS AND METHODS

### Supplementary methods

All chemicals were purchased from Sigma-Aldrich, Carbosynth, Jena Bioscience and LGC Biosearch Technologies and used without further purification. Solvents were of HPLC grade and anhydrous solvents were purchased in Sure/Seal bottles with inert atmosphere or dried prior use by an M-BRAUN solvent purification system. Yields of small molecules refer to the mass of the isolated compounds unless otherwise stated. Yields of the DNA strands refers to isolated DNA dissolutions, where the number of nmols was determined via photometric measurements. Reactions were monitored by thin-layer chromatography (TLC) on Merck silica 60 F254 plates and visualised by exposure to UV (254 nm) or by staining with solutions of molybdic acid, potassium permanganate, ninhydrin, vanillin, or p-anisaldehyde. Flash column chromatography was performed using Merck silica gel 60 (230–400 mesh) as stationary phase and dry vacuum column chromatography was performed using Merck Silica Gel 60: 0.015–0.040 mm. Synthetic steps involving small organic azides were performed using the appropriate safety protocols and safety equipment (the work was executed behind the protection of a polycarbonate blast shield). NMR spectra were recorded on a Bruker BioSpin GmbH Ascend™ 400 and were calibrated using deuterated solvents. ^1^H NMR was recorded at 400 MHz, ^13^C NMR was recorded at 101 MHz, ^19^F NMR was recorded at 376 MHz. Chemical shifts are reported in parts per million and following abbreviations were used to explain multiplicities: s = singlet, d = doublet, t = triplet, q = quartet and m = multiplet. Coupling constants are reported in Hz. HRMS was performed using electrospray ionisation on a Bruker Daltonics MicrOTOF.

### Solid phase DNA synthesis

Oligonucleotides were synthesised on a BioAutomation MerMade-12 automated oligonucleotide synthesiser; the preloaded 1000 Å CPG columns were purchased from LGC Biosearch Technologies Ltd. Oligonucleotide synthesis was carried out under standard conditions. The synthesised oligonucleotides were cleaved from solid support using AMA (1:1 40% methylamine/30–33% ammonium hydroxide).

Amino-modified oligonucleotides, prior the cleaving step, were treated with a 1:1 triethylamine:acetonitrile mixture on the solid support for 30 min at room temperature and then rinsed with acetonitrile to remove the cyano-ethyl protecting group. This step was necessary to avoid the formation of the cyanoethyl-amino adduct. Oligonucleotides containing fluorenylmethoxycarbonyl protecting groups were treated with a 20% piperidine solution in DMF on the solid support for 30 min at room temperature. This procedure was done after the triethylamine treatment and prior the cleaving step.

Oligonucleotide solid phase extraction purifications were performed using Agilent Top-DNA columns following the protocol according to the manufacturer specifications. Oligonucleotides purified via HPLC were passed through a Hewlett-Packard Agilent Expand C-18 stationary column using the following methods (solvent A: 0.1 M triethylammonium acetate, pH 7; solvent B: MeCN; gradient: 0% to 20% B over 15 min, 20–70% B 15–20 min or 5% to 20% B over 15 min, 15–70% B 15–20 min). The oligonucleotide masses were confirmed by UHPLC-ESI-TOF on a Shimadzu LCMS-2020 system.

The incorporation of the sulfonylphosphoramidate modifications was done at desired phosphite triester intermediates during the automated oligonucleotide synthesis. A 0.15 M acetonitrile solution of the corresponding sulfonyl azide was used as the oxidising agent, replacing the standard iodine/pyridine oxidation step. The Staudinger reaction between the sulfonyl azide and the solid support-bound 3′,5′-dinucleoside-β-cyanoethyl phosphite formed a stable *N*-modified phosphoramidate containing the modification of interest. A subsequent standard iodine oxidation step was performed immediately afterwards to form the stable phosphodiester on potential unreacted phosphites; this was necessary to avoid unexpected side-reactions. After this step, the synthesis proceeded following the standard protocol, until the full strand was synthesised. In cases where the solubility of the sulfonyl azide was poor in acetonitrile, it is possible to use other solvents.

### DNA melting studies

UV-melting experiments were carried out on a Thermo Scientific Evolution 260 Bio spectrophotometer. Quartz cuvettes (Hellma) were used with a path length of 1 cm in 10 mM sodium phosphate buffer with 100 mM sodium chloride. Oligonucleotides of 1 μM concentration were scanned at a rate of 1°C/min (for both heating and cooling), at a wavelength of 260 nm. Melting temperatures were determined by the first derivative maxima of the hexic equation fit. The DNA melting curves are included as Supplementary Figures S2 and S13.

#### Azobenzene hairpin cis/trans DNA melting studies

To measure the *cis*/*trans* effect of the azobenzene modifications, one sample was measured without irradiation (*trans*). A second identical sample was irradiated for 20 minutes with a 340 nm, 60 mW output LED, until a photo-stationary state (*cis*) was reached; its melting temperature was then measured. A non-modified hairpin strand was also measured as a control. The melting curves of the hairpins are included as Supplementary Figures S14–S17.

### Pyrene excimer fluoresence studies

These fluorescence studies were performed by using a Horiba Scientific instrument. High precision cell (Quartz cuvette, Hellma) was used at light path 3 × 3 mm. Samples were excited at 340 nm and the emission recorded from 350 to 650 nm at 22°C. Slits for excitation and emission were 5 nm. The single-stranded excimer sample consisted of a 0.25 μM solution of the pyrene modified strand in 10 mM, pH 7.4 phosphate buffer and 100 mM NaCl. The double-stranded analogue was made by taking the former solution and adding the complementary strand (100 μM) from a concentrated stock solution to reach a 1 μM concentration. The recorded data is shown in Figure 3.

### Organic synthesis of the sulfonyl azide probes and bioconjugation protocols

The 11 different probes were synthesised over a total of 25 synthetic steps. The different bioconjugation protocols were carried out using different conditions depending on the specific chemistry of the chemical handles. The experimental details and characterisation for these protocols can be found in the supplementary data. HPLC chromatograms and MS analysis of the bioconjugates are included as Supplementary Figures S18–S22.

## RESULTS

### Synthesis of sulfonyl azides

**Figure 1.**
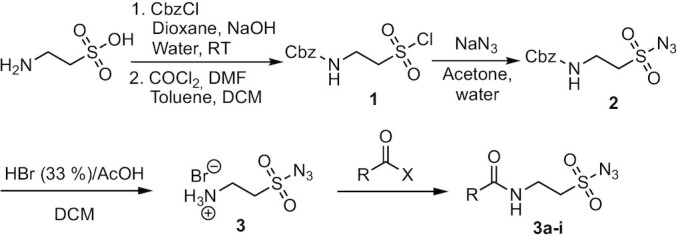
General synthesis of the functionalised sulfonyl azides.

The synthesis of sulfonyl azides proceeded with taurine as the starting material to ensure the linker was small and flexible (26,27). The synthetic pathway was straightforward; it began with the protection of the amino group with benzyl chloroformate, followed by the formation of the sulfonyl chloride, and subsequent nucleophilic substitution with sodium azide (26). The last step required for the removal of the carboxybenzyl (Cbz) protecting group was using HBr in acetic acid, which formed the hydrobromide salt (Figure 1). Using this synthetic pathway, we were able to accomplish a multi-gram scale synthesis of 2-aminoethane-sulfonyl azide hydrobromide (3) with an 81% overall yield. As small azides can potentially be explosive, the synthetic steps involving small organic azides was therefore performed using the appropriate safety protocols and equipment.

This sulfonyl azide-ammonium salt served as a chemical anchor for the incorporation of modifications to the sulfonyl azide moiety. In most cases, this was done by coupling the respective carboxylic acid in the presence of benzotriazol-1-yloxytripyrrolidinophosphonium hexafluorophosphate (PyBOP). In the case of the trifluoroacetate (TFA) protected amino handle, TFA anhydride was used. The fluorenylmethoxycarbonyl (Fmoc) protected amine was synthesised using the protocol reported by Liskamp (26), again starting from taurine, as described for the synthesis of **3**. For the incorporation of the dansyl fluorophore, the reaction was carried out with commercially available dansyl chloride and **3** in the presence of diisopropylethylamine (DIPEA).

**Figure 2.**
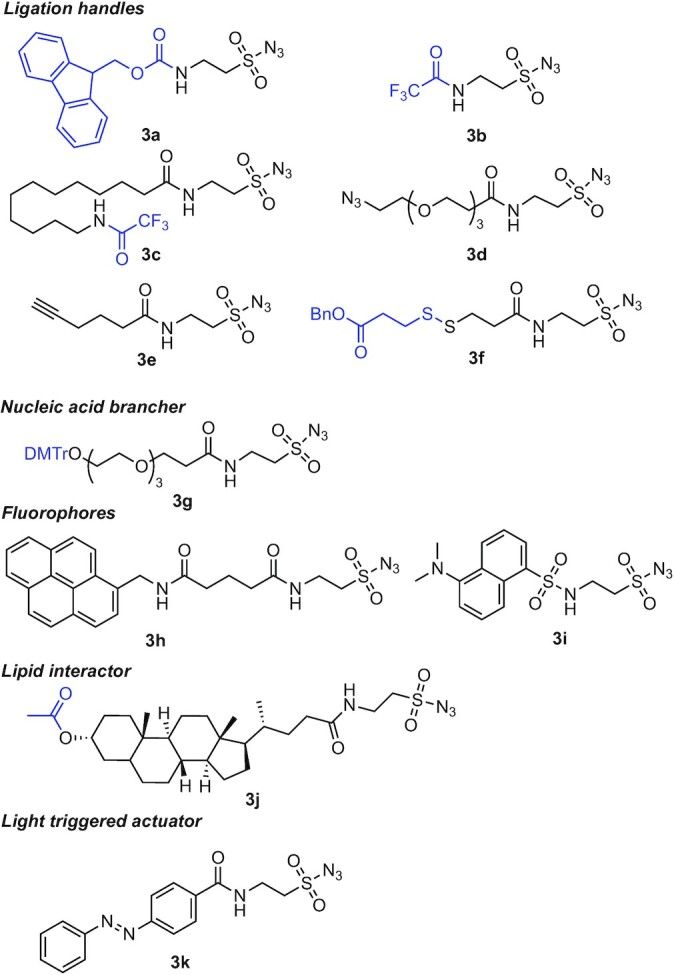
Structures of the 11 synthesised sulfonyl azides probes. Protective groups are shown in blue, and they are in all cases but **3g** replaced by a proton in the final DNA sequence.

A total of 11 sulfonyl azides with different functionalities were synthesised (Figure 2); six of which were chemical handles for bioconjugation: three with protected amines (**3a–c**), an azide (**3d**), an alkyne (**3e**) and a thiol group protected as a disulfide (**3f**). A DMT-protected alcohol (**3g**) was prepared for making branched oligonucleotides. Sulfonyl azides were also considered for incorporation of fluorophores into oligonucleotides and for this purpose we synthesised two sulfonyl azides fluorophores, pyrene (**3h**) and dansyl (**3i**). Miscellaneous groups such as lithocholic acid (**3j**) for interactions with lipids and azobenzene (**3k**) for light-triggered actuation were also developed.

### Modification of DNA oligonucleotides using modified sulfonyl azides via the Staudinger reaction

**Figure 3.**
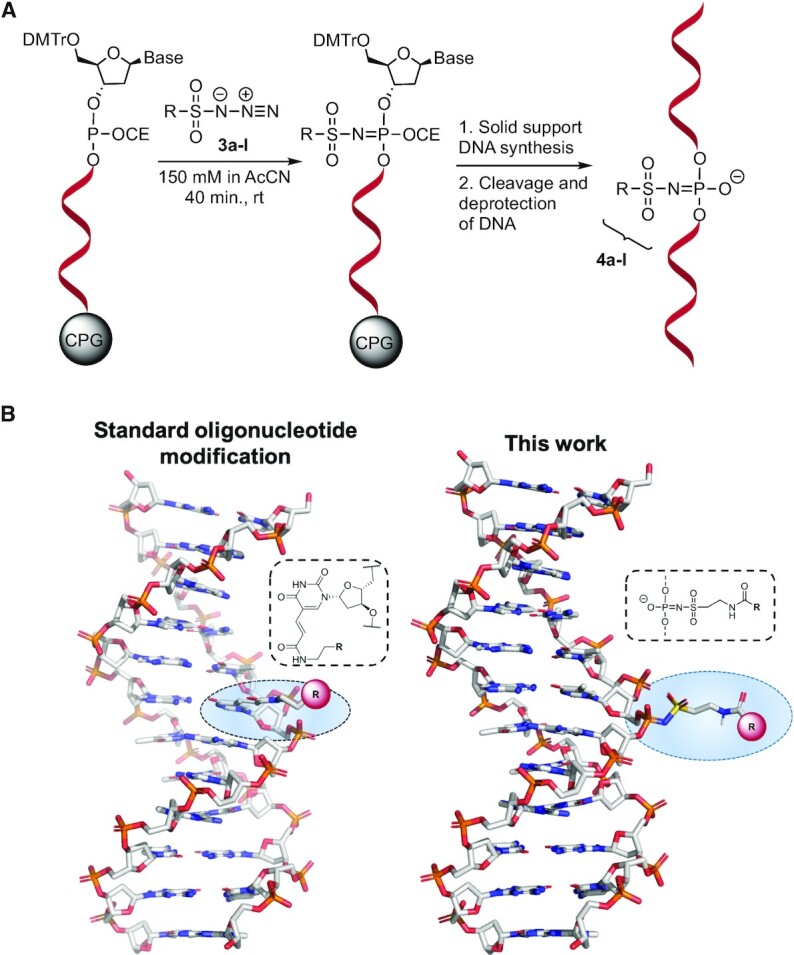
Functionalisation of DNA during solid phase oligonucleotide chemistry. (**A**) Reaction of a phosphite triester with the sulfonyl azide and subsequent completion of the DNA synthesis. (**B**) Comparison of the positioning of a chemical modification in a DNA helix by special phosphoramidites where it is placed in the major groove and this work where it is placed on the phosphate edge of the helix (Only one diastereomer of the sulfonylphosphoramidate is shown).

The incorporation of modifications was achieved with desired phosphite triester intermediates during the automated oligonucleotide synthesis. A 0.15 M acetonitrile solution of the corresponding sulfonyl azide was used as the oxidising agent, replacing the standard iodine/pyridine oxidation step. The Staudinger reaction between the sulfonyl azide and the solid support-bound 3′,5′-dinucleoside-β-cyanoethyl phosphite formed a stable N-modified amidophosphate containing the modification of interest. A subsequent standard iodine oxidation step was performed immediately afterwards to form the stable phosphodiester on potential unreacted phosphites; this was required to avoid undesired side-reactions. After this step, the synthesis proceeded following the standard protocol until the full strand was synthesised (Figure 3a). In cases where the solubility of the sulfonyl azide was poor in acetonitrile, it was possible to use other solvents (DMF was used for **3a**, **3h** and **3j** and THF for **3c**). Albeit, the resulting yields were lower. The oligonucleotide cleavage and deprotection step was completed in a 1:1 mixture of concentrated aqueous ammonia:methyl amine (AMA) at 65°C for 20 min. The crude products were purified by RP-HPLC or SPE. Since the sulfonylphosphoramidate is chiral, two diastereomers were formed; however, they were not separated by HPLC. Pure diastereomers may be obtained by employing stereodefined oxazaphospholidine nucleosides as shown for mesylphosphoramidates by Andersen *et al.* (22). The corresponding sulfonylphosphoramidate modifications in the DNA strands were labeled **4a-I** (Figure 3A, Table 1).

**Table 1.** Characterisation and yields of the oligonucleotides synthesised, modified with the different sulfonyl azides (**3a–j**)

Entry	Sulfonyl azide	Oligonucleotide	Mass calc.	Mass found	Isolated yield	*T*_M_^d^ (°C)	Δ*T*_M_
1	**3a**, amine	**5a**: 5′-CCTAATCAAC(**4a**)TCCACTCCCA-3′	6032.0	6031.1	55%^c^	63	+1
2	**3b**, amine^a^	**5b**: 5′-CCTAATCAAC(**4b**)TCCACTCCCA-3′	6032.0	6031.8	61%^c^	63	+1
3	**3c**, amine	**5c**: 5′-CCTAATCAAC(**4c**)TCCACTCCCA-3′	6229.4	6229.7	13%^d^	60.3	-1.7
4	**3c**, amines	**5c3**: 5′-C(**4c**)CTAATCAAC(**4c**)TCCACTCCC(**4c**)A-3′	6836.3	6836.0	4%^d^	62.8	+0.8
5	**3d**, azide	**5d**: 5′-CCTAATCAAC(**4d**)TCCACTCCCA-3′	6261.3	6260.8	10%^d^	62	0
6	**3e**, alkyne	**5e**: 5′-CCTAATCAAC(**4e**)TCCACTCCCA-3′	6126.2	6125.4	25%^d^	62	0
7	**3f**, thiol	**5f**: 5′-CCTAATCAAC(**4f**)TCCACTCCCA-3′	6119.9	6119.1	13%^d^	61.5	-0.5
8	**3g**, branch	**6g**: (5′-CCTAATCAA)_2_C(**4g**)TCCACTCCCA-3′	8966	8964.8	22%^d^	_	_
9	**3h**, pyrenes	**5h2**: 5′-CCTAATCA(**4h**)ACTCC(**4h**)ACTCCCA-3′	6793	6791.3	10%^d^	70.6	+8.6
10	**3i**, dansyl	**5i**: 5′-CCTAATCAAC(**4i**)TCCACTCCCA-3′	6265.3	6265.0	29%^d^	60.1	-1.9
11	**3j**, steroid	**5j**: 5′-CCTAATCAAC(**4j**)TCCACTCCCA-3′	6390.6	6390.4	13%^d^	58.5	-3.5
12	**3b, 3d, 3e**	**5bde**: 5′-C(**4e**)CTAATCAAC(**4d**)TCCACTCCC(**4b**)A-3′	6567.7	6567.4	36%^d^	60	-2
13	**3k**, azobenz.	**5k**: 5′-CCTAATCAAC(**4k**)TCCACTCCCA-3′	6240.3	6239.8	21%^d^	63	+1
14	**3k**, azobenz.	**8k4**: 5′-ATCTACATATTTT T(**4k**)AT(**4k**)GT(**4k**)AG(**4k**)AT-3′	7970.8	7970.8	16%^d^	_	_
15^b^	**3d**, azide	**7d1**′: 5′-TTTTTTT(**4d**)TTTTTTT-3′	4532.2	4531.4	90%^e^	_	_
16^b^	**3d**, azide	**7d2**′: 5′-T(**4d**)TTTTTTTTTTTTT-3′	4532.2	4531.6	90%^e^	_	_
17^b^	**3e**, alkyne	**7e**: 5′-T(**4e**)TTTTTTTTT-3′	3180.3	3179.3	^f^	_	_

^a^Modifications derived from **3a** and **3b** are identical (**4a** = **4b**, **5a** = **5b**).

^b^Experiment used for the optimisation of reaction time.

^c^Isolated yield from SPE purification.

^d^Isolated yield from RP-HPLC purification.

^e^Isolated yield obtained directly from the crude.

^f^ *T*_M_ of the duplex with a complementary sequence.

When a modification is positioned in the middle section of a DNA strand by special phosphoramidites, it is typically extending from a base in the major groove after hybridisation to a complementary DNA strand (Figure 3B). In this work, the sulfonylphosphoramidate linkage places the modification in the exterior part of the helix; this potentially may give rise to less interaction with the DNA helix.

The Staudinger reaction between the sulfonyl azides and the solid supported phosphite triester is slow compared to the standard oxidation protocol. To optimise the reaction, the reaction time for the Staudinger reaction in the synthesis of poly-T 10-sequence **7e** was investigated. In the synthesis of this sequence, the final phosphite intermediate was subjected to the sulfonyl azide **3e** at different coupling times (Supplementary Figure S1A). Compound **3e** was selected due its hydrophobicity and solubility. It was sufficiently hydrophobic to yield a good separation from the unreacted strand and it was highly soluble in acetonitrile.

The coupling yields were calculated from the areas on the RP-HPLC chromatogram. It was observed that after 40 min the yield reached a plateau, where it increased from 89.6% to 92.1% along 20 min of reaction time (Supplementary Figure S1B). Upon inspection of the masses of the compounds, it was found that the MS of the product obtained after 10 minutes of elution time was consistently the expected modified 10-mer product. From the peak appearing after 9-min elution, the mass corresponded to the unmodified poly-T 10-mer. This was the same for reaction times of 10, 20 and 30 min. The remaining 8–10% of non-modified strands remaining after 40 min reaction time (9 min elution time) did not match the unmodified 10-mer. Presumably, it was composed of truncated DNA strands along with other by-products.

Seventeen different DNA strands with sulfonyl-phosphoramidate modifications were synthesised following the previously described protocol. In the case of **3a**, a concentration of 0.15 M in AcCN was close to saturation, and sometimes it caused precipitation. Furthermore, the Fmoc protecting group must be removed post-synthetically via piperidine treatment, prior to the cleaving and deprotection step. A solution to this problem was the synthesis of **3b**, which is a more soluble molecule. It was further deprotected by standard SPOS cleaving conditions, without the mentioned post-synthetic step. A drawback of the amino modifications **3a** and **3b** that result in the same oligonucleotide (**5a** = **5b**, Table 1) was their low hydrophobicity. If the sulfonyl azide coupling did not go to full conversion, the unmodified DNA strands co-eluted with the desired product during HPLC purification, making it difficult to fully purify them by standard methods. This problem became apparent when we attempted to incorporate three amino handles in the same strand. The LCMS of the isolated peak showed a mixture of co-eluted mono-, di-, and tri-modified strands. The desire for a more hydrophobic amino handle resulted in the synthesis of **3c**. This modification allowed purification of the desired oligonucleotides **5c3** (Table 1) via RP-HPLC, where the separation of the mono-, di- and tri-modified strands was clear.

The introduction of azides into oligonucleotides presents a special challenge due their reactivity towards phosphorous(III) species. Hence, azide-containing phosphoramidites are unstable and degrade rapidly by intra- or intermolecular Staudinger reactions (9,28,29). The usual way around this problem involves the synthesis of an amino-modified oligonucleotide, followed by post-synthetic conjugation with an azide-containing NHS-ester. The use of **3d** proved to be an effective way to introduce azide groups without the need of post-synthetic processes. An initial concern in relation to this method was the possible Staudinger side-reactions of the introduced aliphatic azide with each subsequent phosphoramidite coupling cycle. To test the potential azide degradation during subsequent synthetic steps, two poly-T sequences were synthesised; one in which **3d** was introduced in the middle of the strand (Table 1, entry 15), and another one where it was introduced at the last phosphate of the synthesis (Table 1, entry 16). In the latter case, the azide is not exposed to any phosphoramidite coupling steps. The yield and MS results for both strands showed no difference, confirming no appreciable degradation of the incorporated aliphatic azide occurred during the phosphoramidite coupling cycles. Hence, this is one of the few methods to introduce an azide handle to DNA during solid phase synthesis and it can be introduced at any phosphorous of the strand (28,29).

The sulfonyl azide **3e** served as a novel reagent to incorporate an alkyne into oligonucleotides without the need of special phosphoramidites, as shown in entries 6, 12 and 17 in Table 1. The disulfide **3f** enabled modification of the DNA strand with a thiol after cleavage of the disulfide. The isolated yield of 13% was, however, only moderate. Different by-products resulting from oxidised sulfur species, such as sulfonic and sulfinic acids, and from dimerised DNA strands, were identified. The multiple oxidation steps in the DNA synthesis cycle, in addition to the harsh nucleophilic and basic conditions used during the solid support cleavage/deprotection can degrade the disulfide bond (30). Together, the chemical handles introduced in **5a–f** represent the four most commonly used chemical handles used for conjugation to oligonucleotides. Another chemical interesting moiety is the DMT protected alcohol in **3g** which can be used to prepare branched oligonucleotides as described further below.

The ability to incorporate fluorophores into oligos is valuable for their many applications in nucleic acid technology. Both dansyl and pyrene moieties have been reported to be conjugated to DNA strands for several applications (31–38). The probes **3h** and **3i** proved to successfully incorporate these functionalities into the DNA strands with moderate to good yields. This shows that the method works for the incorporation of dyes (if they are compatible with the solid-phase chemical cycles) into oligonucleotides.

In the case of modifications for lipid interactions, the steroid derivative lithocholic acid, was the molecule of choice. It is commercially available, cheap, and structurally similar to cholesterol, which has been used as interactor with lipid membranes of artificial and living-cell origin (39–41). The sulfonyl azide **3j** incorporated with 13% yield, which is moderate for oligonucleotide synthesis.

In a similar manner, azobenzene can also be incorporated into nucleic acids with sulfonyl azides (**3k**). Azobenzenes are widely used in DNA-nanotechnology due to their *cis*/*trans* photo-switching behaviour (42). The incorporation of sulfonyl azide **3k** proceeded smoothly with a yield of 16%. This may seem moderate, but it should be considered that the strand **5k** contained four incorporations in the same strand.

### Melting behaviour of the sulfonylphosphoramidate modified DNA strands

A 20-mer oligonucleotide sequence (5′-TGGGAGTGGAGTTGATTAGG-3′) which is complementary to the sequences in Table 1, entries 1–13, was used to study the changes in melting temperatures (*T*_M_). The *T*_M_ of the duplexes with the unmodified complementary sequence is 62°C. To our satisfaction, most of the modifications (except for **5h**) caused only minor changes in the *T*_M_ as shown in Table 1.

### Conjugation to the sulfonyl phosphoramidate-linked handles

**Figure 4.**
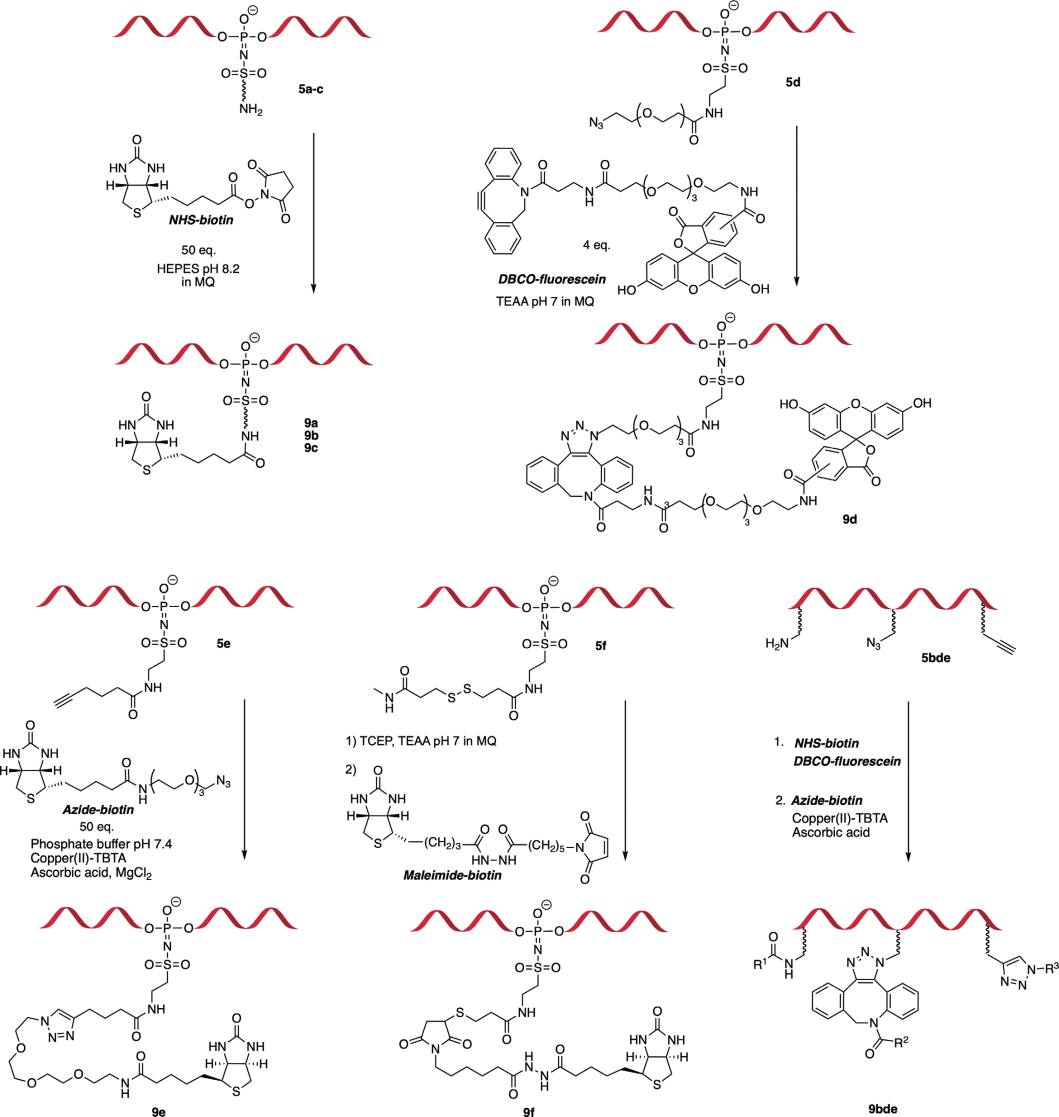
Conjugation tests of the sulfonyl phosphoramidate handles.

The chemical handles incorporated via the Staudinger reaction were functionally tested in a series of reactions with groups of complementary reactivity. Modifications introduced in **5a**-**f** were used in bioconjugation protocols (Table 2). Oligonucleotides **5a,c** modified with amino-groups were tested in a reaction with biotin-NHS ester at pH 8.2, in aqueous HEPES buffer (Figure 4 and Table 2). The hydrophilic amino modification in **5a** showed full conversion into **9a** when reacted under these conditions, while **5c** yielded **9c** in 62%. We hypothesised that the hydrophobicity of the linker led to poor solvation of the reacting group, making it less available for reaction. To test this, a second conjugation protocol was used, where the solvent system was changed to 1:1 water:DMSO. Under these conditions, the reaction with **5c** afforded full conversion to **9c**. To test the reactivity of the azide-modified oligonucleotide **5d**, a modified DNA 20-mer (Table 1, entry 5) was reacted with DBCO-PEG_4_-5/6-carboxyfluorescein overnight, leading to full conversion to **9d**.

**Table 2.** Characterisation and coupling yield of the post-synthetically modified DNA strands with bioconjugation handles

Oligo product	Mass calculated (Da)	Mass found (Da)	Conjugation yield (%)^a^
**9a**	6258.3	6257.7	Quant.
**9c**	6455.6	6457.7	62^b^ & Quant.^c^
**9d**	7143.2	7143.1	Quant.
**9e**	6570.5	6570.4	85
**9f**	6571.7	6571.5	36

^a^Yield calculated using the chromatogram area of each peak.

^b^Yield obtained in deionised water solution.

^c^Yield obtained in a 1:1 MQ:DMSO solution.

The functionality of the alkyne modification in **5e** was tested via CuAAC, which involved reacting a modified DNA 20-mer with azide-PEG_3_-biotin, using Cu(II) in the presence of TBTA and ascorbic acid as the reducing agent (Figure 4, Table 2, entry 6). The essay yielded the expected product **9e** in 85%. In the case of **5f**, a DNA 20-mer (Table 2, entry 7) was conjugated with biotin-maleimide in the presence of TCEP, and obtained the expected product **9f** in 36% yield.

To demonstrate the power of the method, we next attempted to incorporate three different chemical handles (using **3b**, **3d** and **3e**) in the same DNA 20-mer **5bde** (Table 1, entry 12). The synthesised oligonucleotide contained an amino, an azide and an alkyne group in the same strand; an overall HPLC-isolated yield of 36% was achieved.

### Synthesis of a branched oligonucleotide

**Figure 5.**
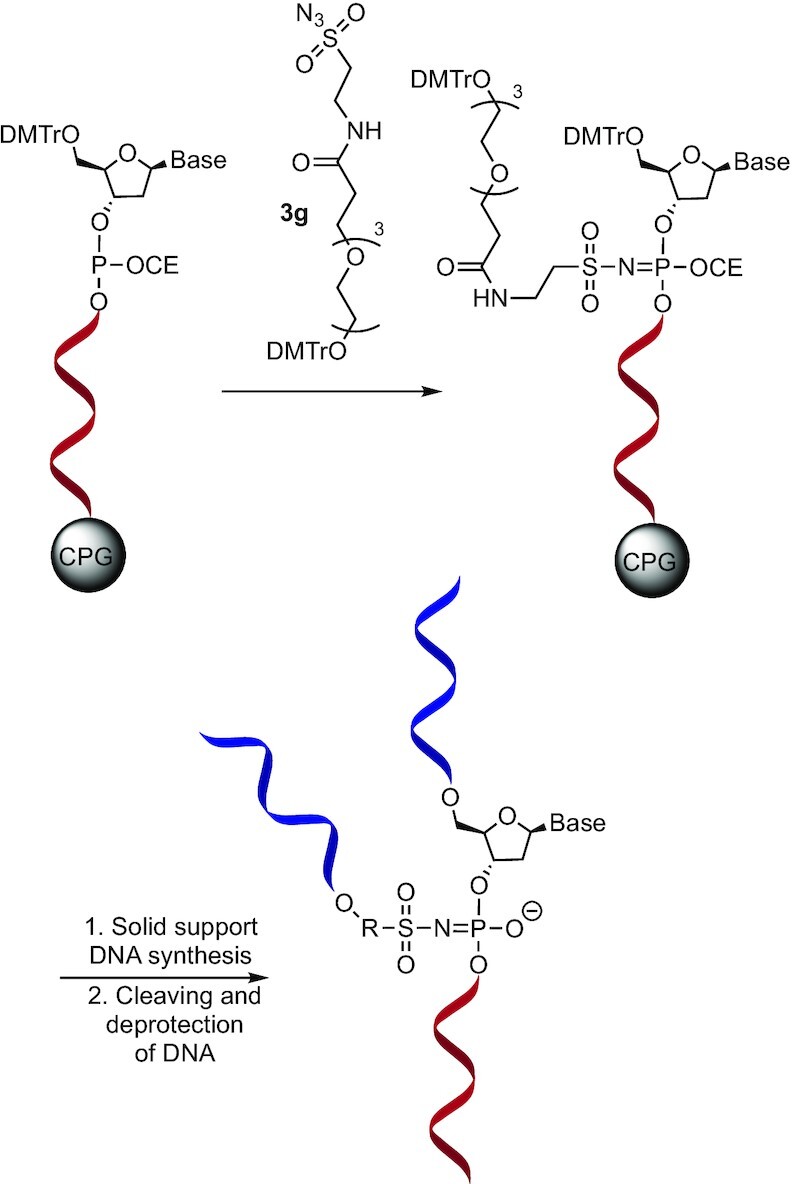
Synthesis of a symmetrical-branched DNA strand with using sulfonyl azide **3g**.

The sulfonyl azide **3g** was tested for the synthesis of the branched sequence **6g**. The DMT protected alcohol modification was inserted at the 10th phosphate. After the subsequent acidic deprotection of the 5′ and modified alcohols, the synthesis proceeded from two reactive alcohols in parallel, yielding a branched DNA-strand **6g** with symmetric sequences in the branched region (Figure 5). The product was then cleaved, deprotected, and purified by RP-HPLC; an expected 22% yield was achieved (Table 1, entry 8). The product was characterised by LC–MS and showed the expected mass.

### Functional tests of the pyrene and azobenzene functionalised sequences

#### Formation and disruption of pyrene excimers in DNA, introduced via sulfonylphosphoramidate modifications

**Figure 6.**
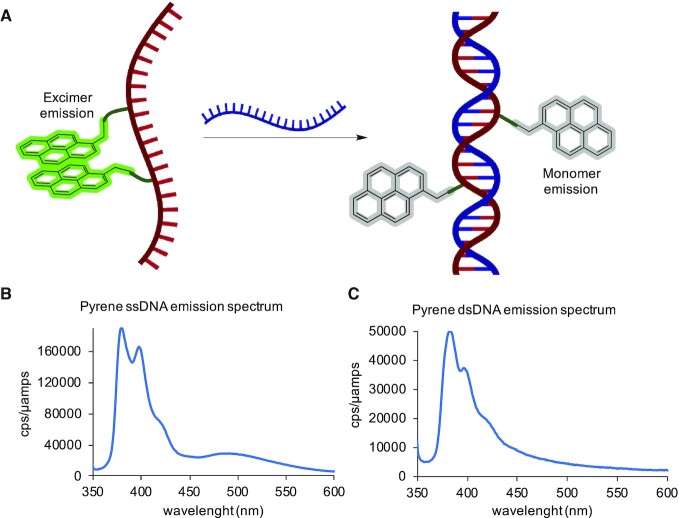
Functionality essay for the pyrene-modified DNA strand (sequence: 5′-CCTAATCA_(_**_3h_**_)_ACTCC_(_**_3h_**_)_ACTCCCA-3′). (**A**) Graphical representation of the excimer emission quenching upon formation of the DNA-duplex. (**B**) Emission spectra of pyrene-modified single stranded DNA excited at 340 nm. (**C**) Emission.

The sequence modified with two pyrenes **5h2** was tested for its special fluorescence properties. When two pyrenes are in close proximity, they can stack on top of each other and form an excimer upon excitation. It is a short-lived excited dimer which has a different absorption and emission spectra compared to the monomer. One characteristic feature of the excimer is a broad emission band at 500 nm (31). This has been exploited in DNA nanotechnology to generate nano-devices where oligonucleotides with pyrene modifications are used as probes to determine location, proximity, and assembly of the different motifs in secondary and tertiary structures (31–33,35,36). Based on this, we designed a simple experiment to test the pyrene modification. The single-stranded DNA 20-mer **5h2** was modified with **3h** at the 7th and 12th phosphate (sense: 3′ to 5′, sequence in Table 1, entry 9). In accordance with the work completed by Ejlersen *et al.* (32), we designed this DNA strand where two pyrene moieties in the single-strand would be close enough to form the excimer, given the flexibility of the strand and the length of **3h** linkers. By positioning the pyrenes 5 nucleobases apart, the modified phosphates would extend from opposite sides of the double helix when a duplex is formed. Therefore, upon addition of a complementary strand, excimer formation should not be allowed (Figure 6A).

Upon excitation at 340 nm the emission spectrum of single stranded **5h2** showed a main emission band between 350–440 nm, corresponding to the monomer fluorescence, while also showing a small band in the 450–550 nm region, indicating the partial formation of the pyrene excimer (Figure 6B). Upon addition of the complementary strand, the excimer band disappeared (Figure 6C), and the emission intensity decreased drastically. This corroborates that, upon formation of the duplex, the excimer cannot be formed.

#### Azobenzene-based light-triggered DNA-hairpin melting, via sulfonylphosphoramidate modification

The last functionalisation test was the *cis/trans* photo-switching of the azobenzene(s) in **5k** and **8k4** (Table 1, entries 13 and 14). Azobenzene units have been widely used as photoactuators in DNA-based devices due to their *cis*/*trans* photo-switching properties. The planar *trans*-configuration intercalates in nucleic acid duplexes, while the *cis*-configuration is non-planar and bulky, destabilising the duplex in which the azo-unit is intercalated. This phenomenon was used to hybridise/denature duplexes upon specific light irradiation, generating an actuation.

The most commonly used method to incorporate azobenzene units involves the use of threonine phosphoramidites with an azo-group as a base surrogate (43). Using solid phase synthesis, the azobenzene phosphoramidite was incorporated as part of the nucleic acid sequence which introduced a gap in the phosphate backbone. The azobenzenes now lie intercalated between bases, forming the double helix (43). Using the sulfonyl azide **3k** we aimed to introduce azobenzene to the phosphate backbone and investigate its switching ability and how that affect the duplex stability.

**Figure 7.**
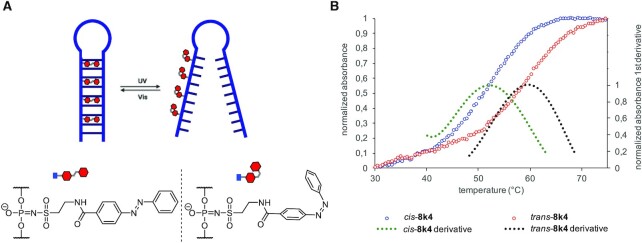
DNA Azo-hairpin (**8k4**) light-triggered denaturalisation via *cis*/*trans* isomerisation of the azobenzene units. (**A**) Graphical representation of the light-triggered hairpin denaturalisation. (**B**) Melting curve of **8k4** for its *cis*/*trans*-azobenzene configuration.

In an initial test, one **3k** unit was introduced in the middle of the DNA 20-mer **5k** (Δ*T*_m_ = +1°C, Table 1, entry 13). The slight increase of the melting temperature indicated that the azobenzene moiety did intercalate the duplex, which led us to develop more complex experiments. An azo-hairpin (**8k4**) consisting of a self-complementary section of 9 base pairs and four thymines in the loop was then synthesised (Table 1, entry 14). One side of the complementary motif of the hairpin was modified with 4 azobenzene units, which were introduced each two bases apart (Figure 7A). The hairpin **8k4** was irradiated at 340 nm until the *cis*-photostationary state was reached. The melting temperature was measured and compared to the melting temperature of the *trans* state, as well as the unmodified hairpin (Supplementary Table S1). It should be noted that **8k4** was prone to thermal relaxation, and could reverse back to the *trans*-configuration after only one melting cycle. The melting temperature of **8k4** in its trans state was 2°C higher than the unmodified hairpin, indicating a slight stabilisation of the secondary structure via intercalation and π-stacking of the azobenzene units. The Δ*T*_m_ between the *cis* and *trans*-**8k4** was 8°C (Figure 7B), showing a clear photo-induced actuation on the hairpin.

## DISCUSSION

The use of sulfonyl azides to react with intermediate phosphites in solid phase oligonucleotide synthesis is demonstrated to be an effective and versatile method to introduce handles and functional groups to oligonucleotides. The Staudinger reaction occurs in <1 h, allowing for the introduction of different functional groups in the solid phase synthesis stage, without the need of post-synthetic laborious steps. Moreover, this method enables the introduction of functional groups, such as azides and disulfides which are difficult to build into DNA by conventional methods. We have developed 11 new probes for conjugation to oligonucleotides with different functionalities and tested them individually. This method is compatible with current automated oligonucleotide synthesis protocols and serves as an inexpensive and simple alternative to modified phosphoramidites for the introduction of functionality in oligonucleotides. Regarding the characterisation of the modified oligonucleotides’ thermal stability, we observed that the non-hydrophobic and positively charged amino handle (**5a**) stabilised the duplex slightly (Δ*T*_m_ = +1°C, Table 1, entries 1, 2), while the more hydrophobic amino-modification in **5c** generated a destabilising effect (Δ*T*_m_ = −1.7°C, Table 1, entry 3). On the other hand, when three units of **3c** were incorporated, the effect was reversed (Δ*T*_m_ = +0.8°C, Table 1, entry 4). To explain this, we speculate that the hydrophobic moieties may interact (38). Modifications in **5d–f** showed no significant *T*_m_ change (Table 1, entries 5**–**7). The two pyrene modifications in **3h** showed a significant increase in the melting temperature (Δ*T*_m_ = +8.6°C, Table 1, entry 9). Pyrene-modified oligonucleotides have previously been reported to form more stable duplexes due to intercalation than the unmodified counterparts (29,34). The dansyl modification in **5i** showed decreased thermal stability (Δ*T*_m_ = −1.9°C, Table 1, entry 10) despite its planarity. It is possible that the short length of the linker, coupled with the steric hindrance of the dimethylamino group prevents the structure from intercalating in the duplex. The oligonucleotide with lithocholic acid **5j** (Δ*T*_m_ = −3.5°C, Table 1, entry 11) showed the lowest thermal stability. The thermal stability decrease is consistent with previous work reported with analogous of cholesterol-modified oligonucleotides (44,45).

When we tested the functionality of the individual modifications, we found that the bioconjugation handles yielded the desired products in good to moderate yields, depending on the specific chemistry being carried out. Furthermore, several chemical handles can be installed easily with good to moderate yields in a single strand. As shown in Figure 4, three different modifications in **5bde** can be used in an orthogonal manner to introduce three different moieties, starting with a strain-promoted azide-alkyne cycloaddition to consume the azide on the oligomer alongside an NHS reagent, followed by CuAAC of the terminal alkyne. The ability to synthesise oligomers with several orthogonal groups in same strands proves the versatility and applicability of this method for nucleic acid modification.

The experiments involving photo-responsive behaviours demonstrated the potential that these kind of probes have for sensing or actuation in oligonucleotide devices. In the case of the pyrene modified strand, we observed a steep decline in fluorescence which is consistent with the quenching of the pyrene emission when it intercalates in the DNA duplex, as previously reported in literature (32,33,37). The strands were not optimised for excimer formation and therefore, the excimer band in Figure 6B is relatively weak. Nevertheless, these results show the viability of this modification in the design and synthesis of pyrene-based fluorescent probes. In the case of azobenzene phosphoramidate modified oligonucleotides, this new azobenzene-based sulfonyl azide shows potential as a photo-actuating modification for light triggered DNA denaturalisation, although further optimisation is required for application of the switching mechanism in nanodevices. Expanding on the molecular design of the molecule, such as increasing the length and characteristics of the linker, testing different functional groups in the aromatic system, etc., could lead to fully functional light-triggered actuators. Most importantly, we have shown how easy four such functional moieties can be introduced into a DNA strand by the sulfonyl azides.

An obvious next step involves expanding the diversity of probes that can be attached to nucleic acids. This could, for example, be non-covalent ligation handles for protein interactions like biotin, or amino acids, short peptides, and all kinds of small-molecule antigens used in biosciences, as long as that they are compatible with the oligonucleotide synthesis. Expanding the catalogue of sulfonyl azide fluorophores would also be a natural extension of the technology. Having access to sulfonyl azide versions of more widely used dyes such as those based on cyanine or fluorescein structures may have great usability. For instance, it would allow for a cheaper and easier introduction of several fluorophores within the same strand at specific known distances, not limited by terminal positioning and without introducing disruptions in the phosphate backbone. In general, a large share of the commercially available phosphoramidites on the market used to functionalise oligonucleotides can be adapted to sulfonyl azide probes.

## DATA AVAILABILITY

All the data supporting the findings of this study are available within the article and supplementary information file and from the corresponding author upon reasonable request. An experimental summary for this article is available as a Supplementary Information file.

## Supplementary Material

gkac566_Supplemental_File
