# Supplementary material for: Conjugation of chemical handles and functional moieties to DNA during solid phase synthesis with sulfonyl azides
Source: Nucleic Acids Res. 2022 Jul 8;50(13):7235–46. doi: 10.1093/nar/gkac566 (PMC9303310; doi:10.1093/nar/gkac566)
Supplement: gkac566_Supplemental_File [file gkac566_supplemental_file.docx]

**Conjugation of Chemical Handles and Functional Moieties to DNA during Solid Phase Synthesis with Sulfonyl Azides**

Angel Santorelli^1^, Kurt Gothelf^1,*^

^1^ Department of Chemistry and Interdisciplinary Nanoscience Centre (iNANO), Aarhus University, Aarhus, Central Denmark Region, 8000, Denmark

* To whom correspondence should be addressed. Tel: +45 60202725; Email: [kvg@chem.au.dk](mailto:kvg@chem.au.dk)

**Organic synthesis of probes**

*Synthesis of compound* ***1***

Benzyl (2-(chlorosulfonyl)ethyl)carbamate (1).

Taurine (3.15 g, 24,6 mmol, 1 eq.) was dissolved in an aqueous solution of NaOH (1M, 25 mL), followed by the dropwise addition of Cbz-chloride (5.3 mL, 35.64 mmol, 1.45 eq.) in dioxane (30 mL), and additional NaOH solution (1M, 37.5 mL, also dropwise). Once all the reagents were incorporated, the reaction mixture was left stirring at rt for one hour. After the time, the excess of Cbz-Cl was extracted with EtOAc (2x50 mL). Concentration of the aqueous layer in vacuum, followed by co-evaporation with toluene, and MeOH yielded the crude sodium sulfonate salt (white solid), which was subjected to high vacuum overnight. Subsequently, the adduct was dispersed in 40 mL of DCM, put in an ice-bath, and mixed with a phosgene solution in toluene (20 % w/w, 24 mL, 1.9 eq.) and DMF (2 mL, 1 eq.). The reaction mixture was left stirring for one in the ice-bath, and second hour at rt. After the time, the reaction was filtered through a celite pad and the solvents removed in vacuum. The crude product was purified via flash column chromatography using DCM, yielding a yellow crystalline solid (5.64 g, 20.3 mmol, 82 % over two steps).

**HRMS (ESI) *m/z* [M+H]^+^** calc**.** 278.0248; found 278.0246

**^1^H NMR (400 MHz, CDCl_3_)** δ 7.52 – 7.29 (m, 5H), 5.32 (bs, 1H), 5.13 (s, 2H), 3.96 – 3.77 (m, 4H).

**^13^C NMR (101 MHz, CDCl_3_)** δ 156.2, 136.0, 128.8, 128.6, 128.3, 67.5, 64.9, 36.5

*Synthesis of Compound* ***2***

Benzyl (2-(azidosulfonyl)ethyl)carbamate (2).

A solution of compound **1** (5.64 g, 20.3 mmol, 1 eq.) in acetone (100 mL) was added dropwise over 40 minutes to a NaN_3_ (1.35 g, 1.0 eq.) solution in water (100 mL). The mixture was left stirring for additional 80 minutes at rt. The acetone was then removed in vacuum, and the aqueous layer extracted with EtOAc. The organic layer was then washed with saturated sodium bicarbonate and brine. The organic layer was dried over sodium sulfate, and the solvent removed in vacuum, yielding the desired product as a white powder (5.58 g, 19.6 mmol, 97%).

**HRMS (ESI) *m/z* [M+H]^+^** calc**.** 285.0652; found 285.0642

**^1^H NMR (400 MHz, CDCl_3_)** δ 7.46 – 7.30 (m, 5H), 5.29 (s, 1H), 5.13 (s, 2H), 3.75 (q, *J* = 6.0 Hz, 2H), 3.57 (t, *J* = 5.8 Hz, 2H).

**^13^C NMR (101 MHz, CDCl_3_)** can be found in reference 1

*Synthesis of Compound* ***3***

2-(Azidosulfonyl)ethan-1-aminium bromide (3).

To a suspension of compound **2** (5.58 g, 19.63 mmol) in DCM (70 mL), 40 mL of HBr in AcOH was added (33 % w/w). The reaction was left stirring at rt for 75 minutes after which a precipitate was formed. The mixture was diluted with 180 mL of DCM and filtered. The filtrate was washed with DCM, Et_2_O and dried under vacuum, obtaining the desired product as a white powder (4.21 g, 18.2 mmol, 93 %)

**HRMS (ESI) *m/z* [M]^+^** calc**.** 151.0284; found 151.0291

**^1^H NMR (400 MHz, D_2_O)** δ 4.02 (t, *J* = 6.4 Hz, 2H), 3.59 (t, *J* = 6.4 Hz, 2H).

**^13^C NMR (101 MHz, D_2_O)** δ 51.7, 33.7.

*Synthesis of Compound* ***S1***

(9H-Fluoren-9-yl)methyl (2-(chlorosulfonyl)ethyl)carbamate (1).

A solution of taurine (1.0 g, 7.99 mmol, 1 eq.) in water (17 mL) was adjusted to pH 8-9 by the addition of 1M solution of NaOH. Subsequently, a solution of Fmoc-chloride (2.49 g, 9,64 mmol, 1.21 eq.) in acetonitrile (35 mL) was added, during which the pH was kept at 8-9 by addition of small amounts of 1M NaOH. The reaction mixture was stirred for approximately 1 h. The reaction was quenched by addition of 50 mL of water. The excess of Fmoc-chloride was removed by washing with EtOAc. The aqueous layer was concentrated in vacuum, followed by co-evaporation with toluene, EtOH, MeOH and DCM yielding the crude sodium sulfonate salt as a white powder, which was dried under high vacuum overnight. Subsequently, the adduct was dispersed in 20 mL of DCM, put in an ice-bath, and mixed with a phosgene solution in toluene (20 % w/w, 7.5 mL, 1.6 eq.) and DMF (1.1 mL, 1.6 eq.). The reaction mixture was left stirring at rt for 2.5 h. After the time, the reaction was filtered through a celite pad and the solvents removed in vacuum. The crude product was purified via flash column chromatography using DCM, yielding a yellow crystalline solid (1.47 g, 4.0 mmol, 50 % over two steps).

**^1^H NMR (400 MHz, CDCl_3_)** δ 7.77 (d, *J* = 7.6 Hz, 2H), 7.62 – 7.52 (m, 2H), 7.41 (t, *J* = 7.5 Hz, 2H), 7.32 (t, *J* = 7.4 Hz, 2H), 5.32 (bs, 1H), 4.47 (d, *J* = 6.8 Hz, 2H), 4.22 (t, *J* = 6.8 Hz, 1H), 3.86 (m, 4H).

**^13^C NMR (101 MHz, CDCl_3_)** δ 143.7, 141.5, 128.0, 127.2, 125.1, 120.2, 67.3, 64.9, 47.3, 36.5, 1.2.

Due to the presence of different rotamers the **^1^H NMR** of compound **S1** contains more peaks than anticipated. This has previously been observed for Fmoc protected compounds (1,2).^[1,2]^

*Synthesis of Compound* ***3a***

(9H-Fluoren-9-yl)methyl (2-(azidosulfonyl)ethyl)carbamate (2).

A solution of compound **S1** (431 mg, 1.18 mmol, 1 eq.) in acetone (7.5 mL) was added dropwise to a NaN_3_ (76.5 mg, 1.0 eq.) solution in water (5 mL). The mixture was left stirring for 1.5 h at rt. The acetone was then removed in vacuum, until a water suspension was left. The solid was further precipitated by addition of water (30 mL). The mixture was filtered, and the filtrate was washed with water and an aqueous solution of NaHCO_3_ (5 %), the solid was further dried in high vacuum, yielding the desired product as a white solid (396 mg, 1.06 mmol, 90 %).

**HRMS (ESI) *m/z* [M+H]^+^** calc**.** 373.0965; found: 373.0978

**^1^H NMR (400 MHz, CDCl_3_)** δ 7.77 (d, *J* = 7.5 Hz, 2H), 7.58 (d, *J* = 7.4 Hz, 2H), 7.41 (t, *J* = 7.4 Hz, 2H), 7.32 (t, *J* = 7.4 Hz, 2H), 5.30 (s, 1H), 4.45 (d, *J* = 6.8 Hz, 2H), 4.22 (t, *J* = 6.9 Hz, 1H), 3.74 (q, *J* = 6.0 Hz, 2H), 3.54 (t, *J* = 5.8 Hz, 2H).

**^13^C NMR (101 MHz, DMSO)** δ 156.0, 143.8, 140.8, 127.7, 127.1, 125.1, 120.2, 65.6, 54.2, 46.7, 35.20.

Due to the presence of different rotamers the **^1^H NMR** of compound **3a** contains more peaks than anticipated. This has previously been observed for Fmoc protected compounds (1,2).

*Synthesis of Compound* ***3b***

2-(2,2,2-Trifluoroacetamido)ethane-1-sulfonyl azide

Compound **3** (302 mg, 1.31 mmol, 1eq.) and trifluoroacetic anhydride (0.3 mL, 2.16 mmol, 1.6 eq.) were mixed in DCM (10 mL) and set into an ice-bath. Followed by the dropwise addition of DIPEA (0.7 mL, 4.02 mmol, 3.02 eq.). The mixture was left stirring on the ice-bath for 2 h. The solvent was then removed in vacuum and the crude product purified by flash column chromatography using DCM:EtOAc (20:0 to 19:1), yielding the desired product as a yellow solid (218 mg, 0.89 mmol, 68 %).

**HRMS (ESI) *m/z* [M+Na]^+^** calc**.** 268.9932; found 268.9926

**^1^H NMR (400 MHz, CDCl_3_)** δ 6.97 (s, 1H), 3.93 (q, *J* = 6.0 Hz, 2H), 3.61 (t, *J* = 6.9 Hz, 2H).

**^13^C NMR (101 MHz, CDCl_3_)** δ 54.21, 34.46 (Carbonyl and CF_3_ carbons are not observed due to the complex ^19^F coupling patterns).

**^19^F NMR (376 MHz, CDCl_3_)** δ -75.97.

*Synthesis of Compound* ***S2***

12-(2,2,2-Trifluoroacetamido)dodecanoic acid

To a dispersion of 12-aminododecanoic acid (300.3 mg, 1.39 mmol, 1 eq.) in MeOH (2 mL), ethyl trifluoroacetate (250 µL, 2.10 mmol, 1.5 eq.) was added, followed by the addition of DIPEA (270 µL, 1.55 mmol, 1.1 eq.). The mixture was left stirring at rt for 19 h. The reaction was quenched with 10 mL of 2M HCl solution and the product extracted with EtOAc. The organic phase was washed two times with 2M HCl solution, dried over sodium sulfate, filtered and the solvent removed in vacuum, yielding the desired product as a white powder (428,6 mg, 1.38 mmol, 99 %).

**HRMS (ESI) *m/z* [M-H]^-^** calc**.** 310.1636; found 310.1648

**^1^H NMR (400 MHz, CDCl_3_)** δ 10.02 (bs, 1H), 6.44 – 6.06 (bs, 1H), 3.36 (q, *J* = 6.8 Hz, 2H), 2.35 (t, *J* = 7.5 Hz, 2H), 1.61 (m, 4H), 1.29 (m, 14H).

**^13^C NMR (101 MHz, CDCl_3_)** δ 178.2, 40.1, 33.8, 29.5, 29.4, 29.4, 29.3, 29.2, 29.1, 29.1, 26.8, 24.8 (Amide and CF_3_ carbons are not observed due to the complex ^19^F coupling patterns).

**^19^F NMR (376 MHz, CDCl_3_)** δ -75.94

*Synthesis of Compound* ***3c***

2-(12-(2,2,2-Trifluoroacetamido)dodecanamido)ethane-1-sulfonyl azide

Compound **S2** (337.9 mg, 1.09 mmol, 1 eq.), PyBOP (619 mg, 1.19 mmol, 1.1 eq.) and DIPEA (650 µL, 3.73 mmol, 3.4 eq.) were dissolved in 5 mL of DCM, and left stirring at rt for 1 hour. To this mixture, compound **3** (250.4 mg, 1.08 mmol, 1 eq.) was added. The resulting reaction mixture was left stirring at rt for 20 h. The reaction was diluted with EtOAc, washed with 2 M HCl solution, a 10 % solution of LiCl and brine. The organic phase was dried over magnesium sulfate and the solvent removed in vacuum. The crude product was purified via flash column chromatography using DCM:EtOAc 8:2 yielding the desired product as a white solid (415 mg, 0,94 mmol, 86 %).

**HRMS (ESI) *m/z* [M+Na]^+^** calc**.** 466.1712; found 466.1715

**^1^H NMR (400 MHz, CDCl_3_)** δ 6.28 (bs, 1H), 6.03 (bs, 1H), 3.79 (q, *J* = 6.0 Hz, 2H), 3.56 (t, *J* = 6.9 Hz, 2H), 3.36 (q, *J* = 6.8 Hz, 2H), 2.20 (t, *J* = 7.6 Hz, 2H), 1.61 (m, 4H), 1.30 (m, 14H).

**^13^C NMR (101 MHz, CDCl_3_)** δ 173.8, 55.4, 40.1, 36.6, 34.1, 29.4, 29.4, 29.3, 29.3, 29.1, 29.1, 26.7, 25.5 (Amide and CF_3_ carbons are not observed due to the complex ^19^F coupling patterns).

**^19^F NMR (376 MHz, CDCl_3_)** δ -75.93.

*Synthesis of compound* ***S3***

Tert-butyl 3-(2-(2-(2-hydroxyethoxy)ethoxy)ethoxy)propanoate

Triethylene glycol (26 mL, 193.91 mmol, 4 eq.) and sodium (98 mg, 4.26 mmol, 0.1 eq.) were dissolved in THF (20 mL). To this solution, tert-butyl acrylate (6.15 g, 48 mmol, 1 eq.) was added dropwise and the reaction mixture was left stirring at rt for 22 h. The solution was neutralized to pH 6-7 with 1M HCl solution and the THF was removed in vacuum. The residue was diluted in EtOAc and washed with brine. The organic layer was dried over magnesium sulfate and the solvent removed in vacuum, yielding the desired product as a light-yellow oil (11.15 g, 40.0 mmol, 84 %).

**^1^H NMR (400 MHz, CDCl_3_)** δ 3.77 – 3.54 (m, 14H), 2.61 (bs, 1H), 2.50 (t, *J* = 6.6 Hz, 2H), 1.43 (s, 9H).

**^13^C NMR (101 MHz, CDCl_3_)** δ 171.1, 80.7, 72.6, 70.8, 70.7, 70.5, 70.4, 67.1, 61.9, 36.4, 28.2.

*Synthesis of compound* ***S4***

Tert-butyl 3-(2-(2-(2-azidoethoxy)ethoxy)ethoxy)propanoate

Compound **S3** (1.48 g, 5.31 mmol, 1 eq.) and DIPEA (1.4 mL, 8.04 mmol, 1.5 eq.) were dissolved in DCM (20 mL) and the flask was placed in an ice-bath. To this solution, mesyl chloride (510 µL, 6.59 mmol, 1.25 eq.) was added dropwise and the reaction mixture was removed from the ice-bath. The mixture was left stirring at rt for 13 h. The solvent was removed in vacuum and redissolved in EtOAc. The solution was washed with a 5 % solution of citric acid, followed by a 5 % solution of sodium bicarbonate and brine. The organic layer was dried over magnesium sulfate and the solvent removed in vacuum. The crude product was dissolved in DMF (13 mL) along with sodium azide (524.5 mg, 8.07 mmol, 1.52 eq.) and left stirring at 50 °C for 16 h. The solution was diluted in EtOAc and washed with water, saturated sodium bicarbonate, a 10 % solution of LiCl, and brine. The organic phase was dried over sodium sulfate and the solvent removed in vacuum. The crude product was purified via flash column chromatography using PE:EtOAc 8:2 to 3:7, yielding the desired product as a clear yellow oil (1.34 g, 4.88 mmol, 83 % over two steps).

**^1^H NMR (400 MHz, CDCl_3_)** δ 3.73 – 3.59 (m, 12H), 3.38 (t, *J* = 5.1 Hz, 2H), 2.49 (t, *J* = 6.6 Hz, 2H), 1.44 (s, 9H).

**^13^C NMR (101 MHz, CDCl_3_)** δ 171.0, 80.6, 70.9, 70.8, 70.7, 70.5, 70.2, 67.0, 50.8, 36.4, 28.2.

*Synthesis of Compound* ***S5***

3-(2-(2-(2-Azidoethoxy)ethoxy)ethoxy)propanoic acid

Compound **S4** (1.34 g, 4.42 mmol, 1 eq.) was dissolved in MeOH (12 mL). To this solution, NaOH (1.45 g in 12 mL of water, 36.25 mmol, 8.2 eq.) was added and the mixture was left stirring at 40 °C for 2 h. The solvent was removed in vacuum and the remaining material acidified with concentrated aqueous HCl until a pH below 2 was reached. The solution was extracted with EtOAc and the organic phase washed with brine. The organic solution was dried over sodium sulfate, and the solvent removed in vacuum, yielding the desired product as pale-yellow oil (1.01 g, 4.08 mmol, 92 %).

**^1^H NMR (400 MHz, CDCl_3_)** δ 3.75 (t, *J* = 6.3 Hz, 2H), 3.68 – 3.62 (m, 10H), 3.37 (t, *J* = 5.1 Hz, 2H), 2.62 (t, *J* = 6.3 Hz, 2H).

**^13^C NMR (101 MHz, CDCl_3_)** δ 176.6, 70.8, 70.7, 70.6, 70.5, 70.2, 66.4, 50.8, 34.9.

*Synthesis of Compound* ***3d***

1-Azido-12-oxo-3,6,9-trioxa-13-azapentadecane-15-sulfonyl azide

Compound **S5** (176 mg, 712 µmol, 1 eq.), PyBOP (407 mg, 782 µmol, 1.1 eq.) and DIPEA (600 µL, 3.44 mmol, 4.8 eq.) were dissolved in 5 mL of DCM, and left stirring at rt for 15 minutes. To this mixture, compound **3** (164 mg, 710 µmol, 1 eq.) was added. The resulting reaction mixture was left stirring at rt for 16 h. The solvent was removed in vacuum and the crude purified via dry vacuum column chromatography using EtOAc:heptane from 10:0 to 0:10, yielding the desired product as pale oil (226 mg, 596 µmol, 84 %).

**HRMS (ESI) *m/z* [M+H]^+^** calc**.** 380.1347; found 380.1360

**^1^H NMR (400 MHz, CDCl_3_)** δ 7.14 (bs, 1H), 3.77 (q, *J* = 6.0 Hz, 2H), 3.72 (t, *J* = 5.5 Hz, 2H), 3.69 – 3.63 (m, 10H), 3.60 (t, *J* = 6.0 Hz, 2H), 3.44 (t, *J* = 5.0 Hz, 2H), 2.52 (t, *J* = 5.5 Hz, 2H).

**^13^C NMR (101 MHz, CDCl_3_)** δ 172.7, 70.6, 70.5, 70.3 (2C), 69.9, 67.0, 55.0, 50.8, 36.6, 34.1.

*Synthesis of Compound* ***3e***

2-(Hex-5-ynamido)ethane-1-sulfonyl azide

5-Hexynoic acid (239.7 mg, 2.07 mmol, 1 eq.), PyBOP (1.11 g, 2.13 mmol, 1.0 eq.) and DIPEA (1 mL, 5.74 mmol, 2.8 eq.) were dissolved in DCM (5 mL), and left stirring at rt for 10 minutes. To this mixture, compound **3** (476 mg, 2.06 mmol, 1 eq.) was added. The resulting reaction mixture was left stirring at rt for 16 h. The reaction was diluted with EtOAc and washed with water, 1M HCl solution, saturated sodium bicarbonate, 10 % LiCl solution and brine. The organic layer was dried over magnesium sulfate and the solvent removed in vacuum. The crude product was purified by flash column chromatography using DCM:EtOAc 10:0 to 8:2 yielding the desired product as a white solid (422 mg, 1.73 mmol, 84 %).

**HRMS (ESI) *m/z* [M+H]^+^** calc**.** 245.0703; found 245.0716

**^1^H NMR (400 MHz, CDCl_3_)** δ 6.08 (s, 1H), 3.86 – 3.74 (m, 2H), 3.61 – 3.52 (m, 2H), 2.37 (t, *J* = 7.4 Hz, 2H), 2.27 (td, *J* = 6.8, 2.6 Hz, 2H), 1.99 (t, *J* = 2.6 Hz, 1H), 1.86 (p, *J* = 7.0 Hz, 2H).

**^13^C NMR (101 MHz, CDCl_3_)** δ 172.9, 83.3, 69.6, 55.3, 34.8, 34.2, 23.9, 17.9.

*Synthesis of Compound* ***S6***

3-((3-(Benzyloxy)-3-oxopropyl)disulfaneyl)propanoic acid

Benzyl alcohol (445.4 mg, 4.12 mmol, 1 eq.), *N*-Ethyl-*N*′-(3-dimethylaminopropyl)carbodiimide hydrochloride (792.8 mg, 4.14 mmol, 1 eq.), DMAP (49.5 mg, 405 µmol, 0.10 eq.) and 3,3′-and dithiodipropionic acid (4.33 g, 20.62 mmol, 5.0 eq.) were dissolved in DMF (20 mL) and left stirring at rt for 14 h. The solvent was removed in high vacuum and the remaining oil diluted in DCM. The organic layer was washed with a 10 % LiCl solution to remove any DMF leftover, causing the precipitation of excess dithiodipropionic acid. The biphasic mixture was filtered, and the organic layer separated. The organic phase was further washed with LiCl solution and brine, dried over sodium sulfate and the solvent removed in vacuum. The crude product was purified via flash column chromatography using DCM + 2 % MeOH, yielding the desired product as a yellow oil that slowly crystalizes (352.7 mg, 1.18 mmol, 28 %).

**HRMS (ESI) *m/z* [M+Na]^+^** calc**.** 323.0382; found 323.0388

**^1^H NMR (400 MHz, CDCl_3_)** δ 7.40 – 7.32 (m, 5H), 5.15 (s, 2H), 2.93 (m, 4H), 2.82 – 2.75 (m, 4H).

**^13^C NMR (101 MHz, CDCl_3_)** δ 171.7, 135.8, 128.8, 128.5, 128.5, 66.8, 34.3, 33.8, 33.2, 32.8.

*Synthesis of Compound* ***3f***

Benzyl 3-((3-((2-(azidosulfonyl)ethyl)amino)-3-oxopropyl)disulfaneyl)propanoate

Compound **S6** (150.8 mg, 502.2 µmol, 1 eq.), PyBOP (274 mg, 527 µmol, 1.05 eq.) and DIPEA (270 µL, 1.55 mmol, 3.1 eq.) were dissolved in 5 mL of DCM, and left stirring at rt for 10 minutes. To this mixture, compound **3** (116 mg, 502.2 µmol, 1 eq.) was added. The resulting reaction mixture was left stirring at rt for 4 h. The reaction was diluted with EtOAc and washed with water, 10 % LiCl solution, saturated sodium bicarbonate solution and brine. The organic layer was dried over magnesium sulfate and the solvent removed in vacuum. The crude product was purified by flash column chromatography using DCM:EtOAc 10:0 to 8:2 yielding the desired product as a white solid (166.4 mg, 385 µmol, 77 %).

**HRMS (ESI) *m/z* [M+H]^+^** calc**.** 433.0669; found 433.0696

**^1^H NMR (400 MHz, CDCl_3_)** δ 7.39 – 7.22 (m, 5H), 6.16 (s, 1H), 5.09 (s, 2H), 3.72 (q, *J* = 5.9 Hz, 2H), 3.49 (dd, *J* = 6.7, 4.9 Hz, 2H), 2.88 (dt, *J* = 8.8, 7.0 Hz, 4H), 2.72 (t, *J* = 7.0 Hz, 2H), 2.52 (t, *J* = 7.0 Hz, 2H).

**^13^C NMR (101 MHz, CDCl_3_)** δ 171.8, 171.5, 135.7, 128.8, 128.6, 128.5, 66.9, 55.2, 35.6, 34.3, 34.2, 33.4, 33.3.

*Synthesis of Compound* ***S7***

2-(2-(2-(Bis(4-methoxyphenyl)(phenyl)methoxy)ethoxy)ethoxy)ethan-1-ol (4).

Triethylene glycol (2.80 mL, 20.96 mmol, 2.1 eq.), DMAP (61 mg, 499.3 µmol, 0.05 eq.) and DIPEA (10 mL, 57.4 mmol, 5.7 eq.) were dissolved in 15 mL of DCM. To this mixture, a solution of 4,4′-dimethoxytrityl chloride (3.40 g, 10.03 mmol, 1 eq.) in DCM (20 mL) was added dropwise. The reaction was left stirring at rt for 15 h. The reaction was quenched by the addition of MeOH (20 mL), letting the mixture stir for 30 minutes. The solvent was removed in vacuum and the crude mixture dissolved in EtOAc. The organic solution was washed with water, saturated sodium bicarbonate solution and brine, dried over sodium sulfate and the solvent removed in vacuum. The crude product was purified via flash column chromatography starting with PE:EtOAc 1:1 + 1 % Et_3_N up to EtOAc:MeOH 19:1 + 1 % Et_3_N, yielding the desired product as a yellow oil (3.19 g, 7.05 mmol, 70 %).

**HRMS (ESI) *m/z* [M+Na]^+^**calc**.**475.2091; found 475,2107

**^1^H NMR (400 MHz, CDCl_3_)** δ 7.48 – 7.44 (m, 2H), 7.37 – 7.32 (m, 4H), 7.31 – 7.26 (m, 2H), 7.23 – 7.17 (m, 1H), 6.85 – 6.78 (m, 4H), 3.79 (s, 6H), 3.75 – 3.66 (m, 8H), 3.63 (m, 2H), 3.25 (t, *J* = 5.2 Hz, 2H), 2.36 (t, *J* = 6.1 Hz, 1H).

**^13^C NMR (101 MHz, CDCl_3_)** δ 158.6, 145.2, 136.5, 130.2, 128.4, 127.9, 126.8, 113.2, 86.2, 72.7, 71.0, 70.8, 63.3, 62.0, 55.4, 46.4.

*Synthesis of Compound* ***S8***

Tert-butyl 1,1-bis(4-methoxyphenyl)-1-phenyl-2,5,8,11-tetraoxatetradecan-14-oate

Compound **S7** (1.17 g, 2.60 mmol, 1 eq.), *tert*-butyl acrylate (1.60 mL, 10.92 mmol, 4.21 eq.) and NaH (12 mg of a 60 % dispersion, 300 µmol, 0.12 eq.) were mixed in 20 mL of THF. The reaction was left stirring at rt for 18 h. The solvent was removed in vacuum and the crude dissolved in EtOAc. The organic solution was washed with water, saturated sodium bicarbonate solution and brine, dried over magnesium sulfate and the solvent removed in vacuum. The crude product was purified via flash column chromatography using PE:Et_2_O + 1 % Et_3_N 10:0 to 7:3, yielding the desired product as a pale-pink oil (1.33 g, 2.29 mmol, 88 %).

**HRMS (ESI) *m/z* [M+Na]^+^** calc**.** 603.2928; found 603.2934

**^1^H NMR (400 MHz, CDCl_3_)** δ 7.48 – 7.43 (m, 2H), 7.37 – 7.32 (m, 4H), 7.30 – 7.24 (m, 2H), 7.22 – 7.17 (m, 1H), 6.84 – 6.79 (m, 4H), 3.78 (s, 6H), 3.72 – 3.64 (m, 10H), 3.63 – 3.59 (m, 2H), 3.22 (t, *J* = 5.3 Hz, 2H), 2.49 (t, *J* = 6.6 Hz, 2H), 1.44 (s, 9H).

**^13^C NMR (101 MHz, CDCl_3_)** δ 171.1, 158.5, 145.2, 136.5, 130.2, 128.3, 127.9, 126.8, 113.2, 86.0, 80.6, 70.9, 70.8 (2C), 70.7, 70.5, 67.0, 63.2, 55.3, 36.4, 28.2.

*Synthesis of Compound* ***S9***

1,1-Bis(4-methoxyphenyl)-1-phenyl-2,5,8,11-tetraoxatetradecan-14-oic acid

Compound **S8** (1.32 g, 2.28 mmol, 1 eq.) was dissolved in MeOH (20 mL), to this solution NaOH (794.4 mg, 19.86 mmol, 8.71 eq.) in water (10 mL) was added. The reaction was left stirring at 40 °C 16 h. The MeOH was removed in vacuum and the resulting aqueous solution was acidified to pH 5 with a 1M HCl solution. The water from the mixture was removed in vacuum, followed by co-evaporations with toluene. The crude product was suspended/dissolved in DCM, dried over sodium sulfate, filtered and the solvent removed in vacuum. The crude product was purified via flash column chromatography packing the column with DCM + 3 % Et_3_N and eluting with DCM + 1 % Et_3_N + MeOH (3 % to 10 %), yielding the desired product as a yellow oil (1.18 g, 2.25 mmol, 99 %).

**HRMS (ESI) *m/z* [M-H]^-^** calc**.** 523.2337; found 523.2351

**^1^H NMR (400 MHz, CDCl_3_)** δ 7.47 – 7.43 (m, 2H), 7.37 – 7.31 (m, 4H), 7.30 – 7.17 (m, 3H), 6.85 – 6.80 (m, 4H), 3.79 (s, 6H), 3.72 – 3.62 (m, 14H), 3.52 (dd, *J* = 6.8, 5.4 Hz, 2H), 3.23 (t, *J* = 5.1 Hz, 2H), 2.46 (t, *J* = 5.5 Hz, 2H).

**^13^C NMR (101 MHz, CDCl_3_)** δ 175.2, 158.5, 145.2, 136.5, 130.2, 128.3, 127.9, 126.8, 113.2, 86.1, 70.9, 70.8 (2C), 70.6, 70.5, 67.2, 63.3, 55.4, 35.7.

*Synthesis of Compound* ***3g***

1,1-Bis(4-methoxyphenyl)-14-oxo-1-phenyl-2,5,8,11-tetraoxa-15-azaheptadecane-17-sulfonyl azide

Compound **S9** (279.3 mg, 532 µmol, 1 eq.), PyBOP (304.6 mg, 585 µmol, 1.1 eq.), DIPEA (500 µL, 2.87 mmol, 5.4 eq.) and compound **3** (123.4 mg, 534 µmol, 1 eq.) were dissolved in DCM (8 mL), and left stirring at rt for 20 h. The reaction was diluted with EtOAc and washed with 10 % LiCl solution, saturated sodium bicarbonate solution and brine. The organic layer was dried over magnesium sulfate and the solvent removed in vacuum. The crude product was purified by flash column chromatography using EtOAc + 2 % Et_3_N yielding the desired product as a pale-yellow oil (151 mg, 230 µmol, 43 %).

**HRMS (ESI) *m/z* [M+Na]^+^** calc**.** 679.2408; found 679.2426

**^1^H NMR (400 MHz, CDCl_3_)** δ 7.47 – 7.43 (m, 2H), 7.37 – 7.31 (m, 4H), 7.30 – 7.26 (m, 2H), 7.25 – 7.17 (m, 2H), 6.85 – 6.80 (m, 4H), 3.79 (s, 6H), 3.72 – 3.62 (m, 14H), 3.52 (t, *J* = 5.5 Hz, 2H), 3.23 (t, *J* = 5.1 Hz, 2H), 2.46 (t, *J* = 5.5 Hz, 2H).

**^13^C NMR (101 MHz, CDCl_3_)** δ 172.6, 158.5, 145.2, 136.4, 130.2, 128.3, 127.9, 126.9, 113.2, 86.1, 70.9 (2C), 70.8, 70.4 (2C), 66.95, 63.26, 55.36, 54.87, 36.74, 34.10.

*Synthesis of Compound* ***S10***

5-Oxo-5-((pyren-1-ylmethyl)amino)pentanoic acid

1-Pyrenemethylamine hydrochloride (204.8 mg, 727 µmol, 1 eq.) and glutaric anhydride (216.5 mg, 1.8 mmol, 2.5 eq.) were dissolved in pyridine (20 mL) and left stirring at rt for 24 h in the dark. The pyridine was removed in vacuum, followed by co-evaporation with heptane. The crude oil was dispersed in an HCl solution (5M, 100 mL), and extracted several times with DCM. The organic layer was washed with brine, dried over sodium sulfate and the solvent removed in vacuum, yielding the desired product as a pale-yellow solid (146.5 mg, 424 µmol, 58 %).

**HRMS (ESI) *m/z* [M+H]^+^** calc**.** 346.1438; found 346.1446

**^1^H NMR (400 MHz, DMSO)** δ 12.05 (s, 1H), 8.55 (t, *J* = 5.7 Hz, 1H), 8.39 – 8.21 (m, 5H), 8.16 (s, 2H), 8.08 (t, *J* = 7.6 Hz, 1H), 8.02 (d, *J* = 7.8 Hz, 1H), 5.00 (d, *J* = 5.7 Hz, 2H), 2.23 (q, *J* = 7.7 Hz, 4H), 1.78 (p, *J* = 7.4 Hz, 2H).

**^13^C NMR (101 MHz, DMSO)** δ 174.3, 171.6, 133.1, 130.8, 130.3, 130.1, 128.1, 127.5, 127.4, 127.0, 126.7, 126.3, 125.3, 125.2, 124.7, 124.1, 124.0, 123.3, 40.4, 34.5, 33.2, 20.8.

*Synthesis of Compound* ***3h***

2-(5-Oxo-5-((pyren-1-ylmethyl)amino)pentanamido)ethane-1-sulfonyl azide

Compound **S10** (146 mg, 423 µmol, 1 eq.), PyBOP (238.4 mg, 585 µmol, 1.07 eq.), DIPEA (250 µL, 1.44 mmol, 3.4 eq.) and compound **3** (99.7 mg, 431 µmol, 1.02 eq.) were dissolved in DMF (5 mL), and left stirring at rt for 22 h. The reaction was diluted with EtOAc and washed with 10 % LiCl solution and brine. The organic layer was dried over magnesium sulfate and the solvent removed in vacuum. The crude product was purified by flash column chromatography using EtOAc + MeOH (0 % to 3 %) yielding the desired product as a pale-yellow solid (82 mg, 172 µmol, 40 %).

**HRMS (ESI) *m/z* [M+H]^+^** calc**.** 478.1544; found 478.1573

**^1^H NMR (400 MHz, DMSO)** δ 8.52 (t, *J* = 5.7 Hz, 1H), 8.37 (d, *J* = 9.3 Hz, 1H), 8.33 – 8.23 (m, 4H), 8.16 (s, 3H), 8.08 (t, *J* = 7.6 Hz, 1H), 8.02 (d, *J* = 7.8 Hz, 1H), 5.00 (d, *J* = 5.6 Hz, 2H), 3.85 (t, *J* = 6.4 Hz, 2H), 3.50 (q, *J* = 6.2 Hz, 2H), 2.19 (t, *J* = 7.5 Hz, 2H), 2.13 (t, *J* = 7.5 Hz, 2H), 1.79 (p, *J* = 7.5 Hz, 2H).

**^13^C NMR (101 MHz, DMSO)** δ 172.3, 171.7, 133.1, 130.8, 130.3, 130.1, 128.1, 127.6, 127.4, 127.0, 126.7, 126.3, 125.3, 125.2, 124.7, 124.1, 124.0, 123.3, 54.2, 40.4, 34.7, 33.4, 21.5.

*Synthesis of Compound* ***3i***

2-((5-(Dimethylamino)naphthalene)-1-sulfonamido)ethane-1-sulfonyl azide

Dansyl chloride (234.9 mg, 871 µmol, 1.0 eq.), compound **3** (200.2 mg, 870 µmol, 1.0 eq.) and DIPEA (500 µL, 2.87 mmol, 3.3 eq.) were dissolved in DCM (10 mL), and left stirring at rt for 5 h. The solvent removed in vacuum and the crude purified by flash column chromatography using DCM + EtOAc (0 % to 5 %) yielding the desired product as a yellow solid (208 mg, 542 µmol, 62 %).

**HRMS (ESI) *m/z* [M+H]^+^** calc**.** 384.0795; found 384.0809

**^1^H NMR (400 MHz, CDCl_3_)** δ 8.59 (d, *J* = 8.6 Hz, 1H), 8.26 (dd, *J* = 7.3, 1.2 Hz, 1H), 8.21 (d, *J* = 8.6 Hz, 1H), 7.61 (dd, *J* = 8.6, 7.6 Hz, 1H), 7.55 (dd, *J* = 8.5, 7.3 Hz, 1H), 7.22 (d, *J* = 7.6 Hz, 1H), 5.30 (t, *J* = 6.0 Hz, 1H), 3.45 (m, 4H), 2.90 (s, 6H).

**^13^C NMR (101 MHz, CDCl_3_)** δ 152.3, 133.7, 131.4, 130.1, 130.0, 129.5, 129.0, 123.3, 118.4, 115.7, 55.6, 45.5, 37.8.

*Synthesis of Compound* ***S11***

(4R)-4-((3R,8R,9S,10S,13R,14S,17R)-3-acetoxy-10,13-dimethylhexadecahydro-1H-cyclopenta[a]phenanthren-17-yl)pentanoic acid

Lithocholic acid (734.5 mg, 1.85 mmol, 1.0 eq.), acetic anhydride (750 µL, 7.93 mmol, 4.3 eq.) and pyridine (1.5 mL, 18.62 mmol, 10 eq.) were mixed and heated to 100 °C for 30 minutes. The solution was left to cool down and crashed with water (100 mL). The crude product was extracted with DCM and the organic layer was washed with brine, dried over sodium sulfate and the solvent removed in vacuum. The crude product was purified via flash column chromatography using PE:EtOAc 8:2 to 1:1, yielding the desired product as a white foam (650.7 mg, 1.55 mmol, 84 %).

**HRMS (ESI) *m/z* [M-H]^-^** calc**.** 417.3010; found 417.0325

**^1^H NMR (400 MHz, CDCl_3_)** δ 4.72 (tt, *J* = 11.4, 4.7 Hz, 1H), 2.40 (ddd, *J* = 15.5, 10.2, 5.1 Hz, 1H), 2.26 (ddd, *J* = 15.8, 9.7, 6.5 Hz, 1H), 2.03 (s, 3H), 0.95 – 0.88 (m, 6H), 0.64 (s, 3H).

**^13^C NMR (101 MHz, CDCl_3_)** δ 178.92, 170.86, 74.57, 56.63, 56.11, 42.89, 42.02, 40.54, 40.28, 35.93, 35.46, 35.17, 34.73, 32.39, 30.91, 28.33, 27.16, 26.77, 26.46, 24.32, 23.48, 21.64, 20.97, 18.39, 12.19.

*Synthesis of Compound* ***3j***

(3R,8R,9S,10S,13R,14S,17R)-17-((R)-5-((2-(Azidosulfonyl)ethyl)amino)-5-oxopentan-2-yl)-10,13-dimethylhexadecahydro-1H-cyclopenta[a]phenanthren-3-yl acetate

Compound **S11** (300 mg, 717 µmol, 1 eq.), PyBOP (400.5 mg, 754 µmol, 1.05 eq.) and DIPEA (620 µL, 3.56 mmol, 5 eq.) were dissolved in DCM (5 mL), and left stirring at rt for 30 minutes. To this mixture, compound **3** (164.7 mg, 713 µmol, 1 eq.) was added. The resulting reaction mixture was left stirring at rt for 7 h. The reaction solvent was removed in vacuum and redissolved in EtOAc, washed with water, 1M HCl solution, saturated sodium bicarbonate and brine. The organic layer was dried over magnesium sulfate and the solvent removed in vacuum. The crude product was purified by flash column chromatography using DCM:EtOAc 100:00 to 93:07 yielding the desired product as a white foam (275.6 mg, 500 µmol, 70 %).

**HRMS (ESI) *m/z* [M+H]^+^** calc**.** 551.3262; found 551.3262

**^1^H NMR (400 MHz, CDCl_3_)** δ 6.09 – 5.96 (m, 1H), 4.71 (tt, *J* = 11.3, 4.8 Hz, 1H), 3.84 – 3.73 (m, 2H), 3.62 – 3.50 (m, 2H), 2.32 – 2.06 (m, 2H), 2.03 (s, 3H), 0.91 (m, 6H), 0.64 (s, 3H).

**^13^C NMR (101 MHz, CDCl_3_)** δ 174.24, 170.84, 74.56, 56.62, 56.12, 55.40, 42.90, 42.02, 40.54, 40.28, 35.92, 35.60, 35.17, 34.73, 34.16, 33.49, 32.39, 31.62, 28.39, 27.15, 26.77, 26.45, 24.32, 23.47, 21.64, 20.97, 18.49, 12.19.

*Synthesis of Compound* ***3k***

2-(4-(Phenyldiazenyl)benzamido)ethane-1-sulfonyl azide

4-(Phenyldiazenyl)benzoic acid (203.4 mg, 881 µmol, 1 eq.), PyBOP (515.2 mg, 970 µmol, 1.1 eq.) and DIPEA (650 µL, 3.73 mmol, 4.23 eq.) were dissolved in DMF (7 mL), and left stirring at rt for 15 minutes. To this mixture, compound **3** (203.6 mg, 881 µmol, 1 eq.) was added. The resulting reaction mixture was left stirring at rt for 14 h. The reaction was diluted with EtOAc and washed with water, 10 % LiCl solution, saturated sodium bicarbonate solution and brine. The organic layer was dried over sodium sulfate and the solvent removed in vacuum. The crude product was purified by flash column chromatography using PE:EtOAc 8:2 to 1:1 yielding the desired product as a shiny orange solid (266.4 mg, 743 µmol, 84 %).

**HRMS (ESI) *m/z* [M+H]^+^** calc**.** 359.0921; found 359.0931

**^1^H NMR (400 MHz, CDCl_3_)** δ 8.05 – 7.85 (m, 6H), 7.60 – 7.46 (m, 3H), 6.83 (t, *J* = 5.8 Hz, 1H), 4.14 – 3.96 (m, 2H), 3.78 – 3.62 (m, 2H).

**^13^C NMR (101 MHz, DMSO)** δ 165.8, 153.5, 151.9, 136.0, 132.1, 129.6, 128.6, 122.8, 122.5, 53.9, 34.3.

**NMR Spectra**

^1^HNMR of compound **1**


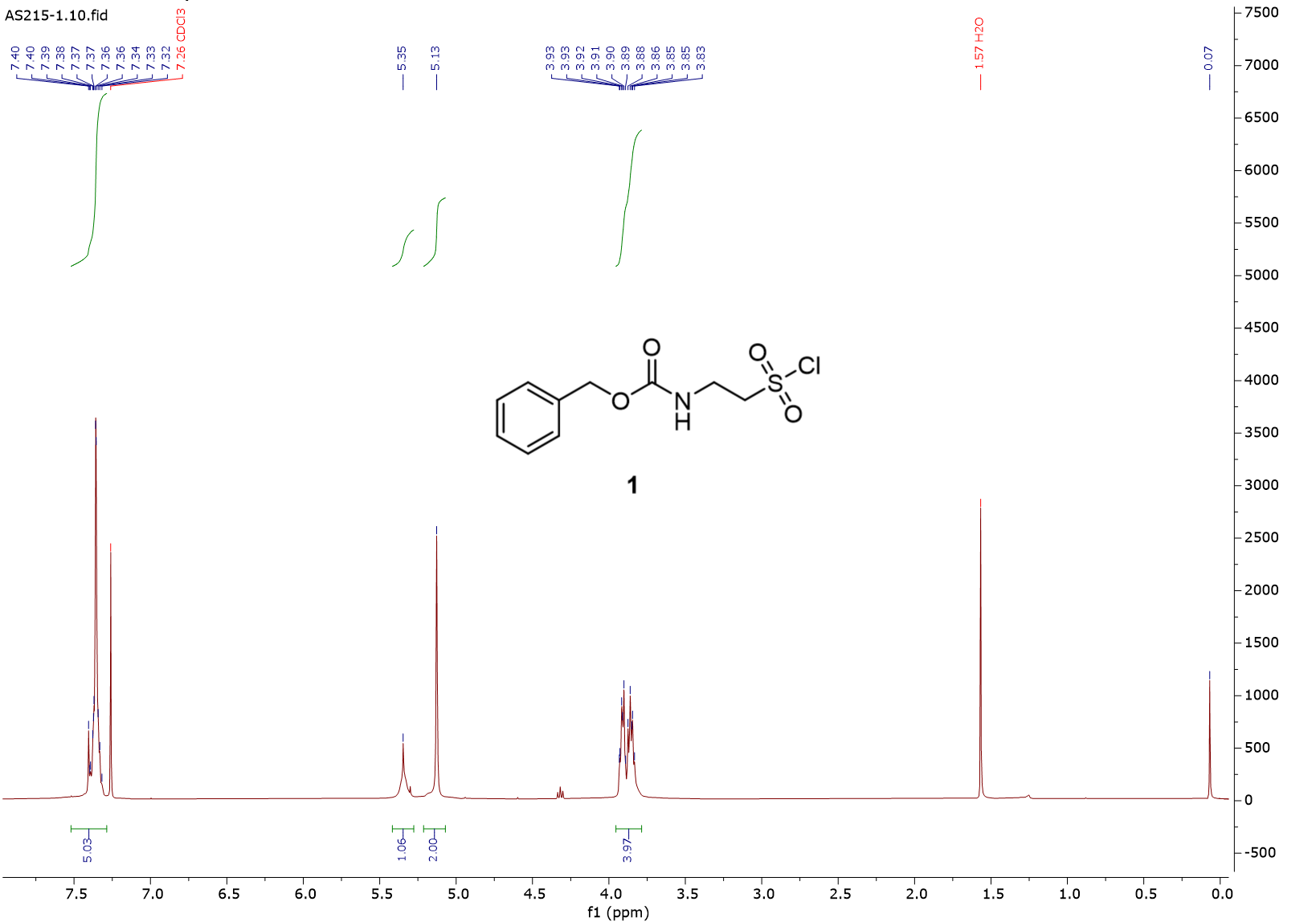


^13^CNMR of compound **1**

**
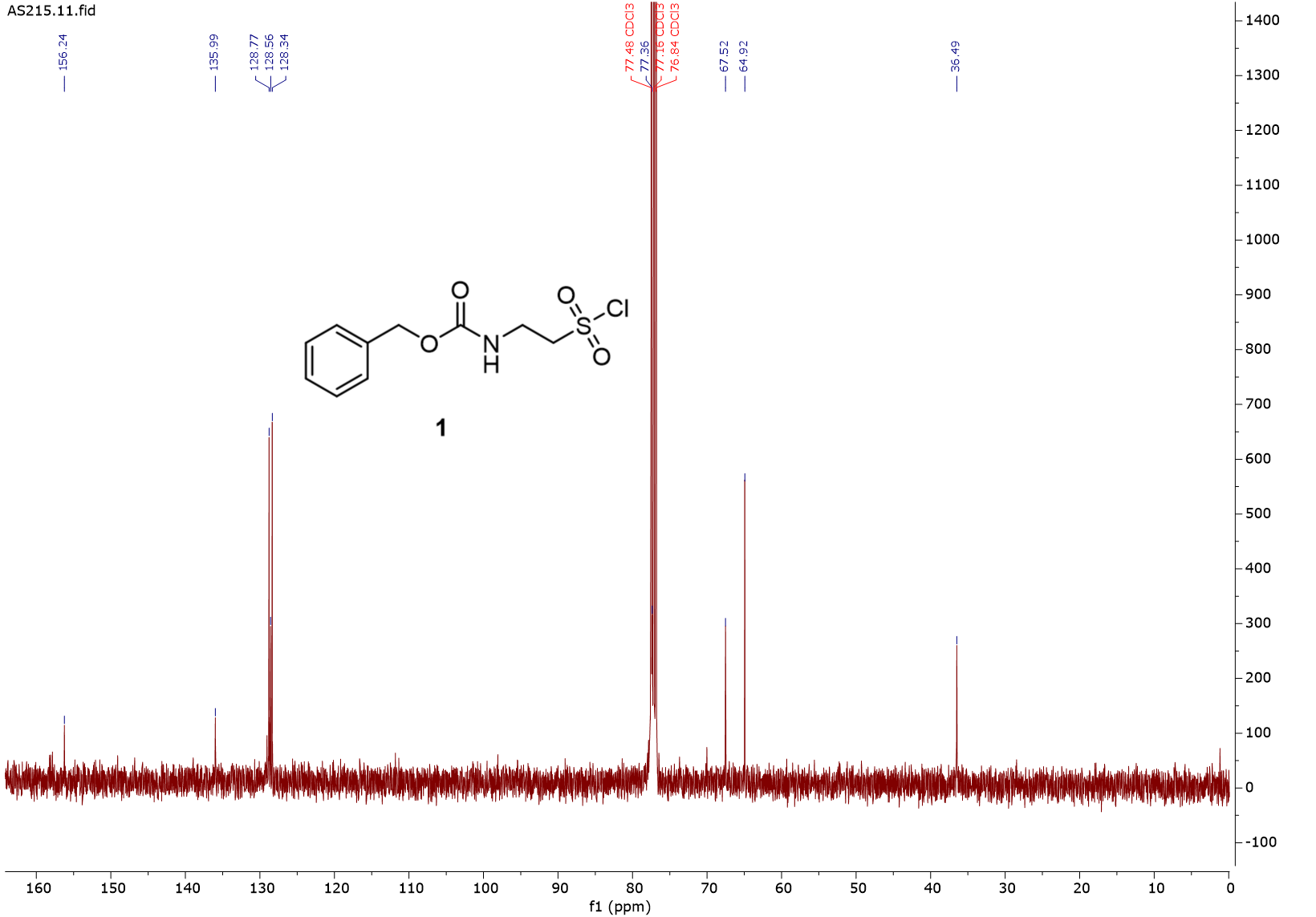
**

^1^HNMR of compound **2**


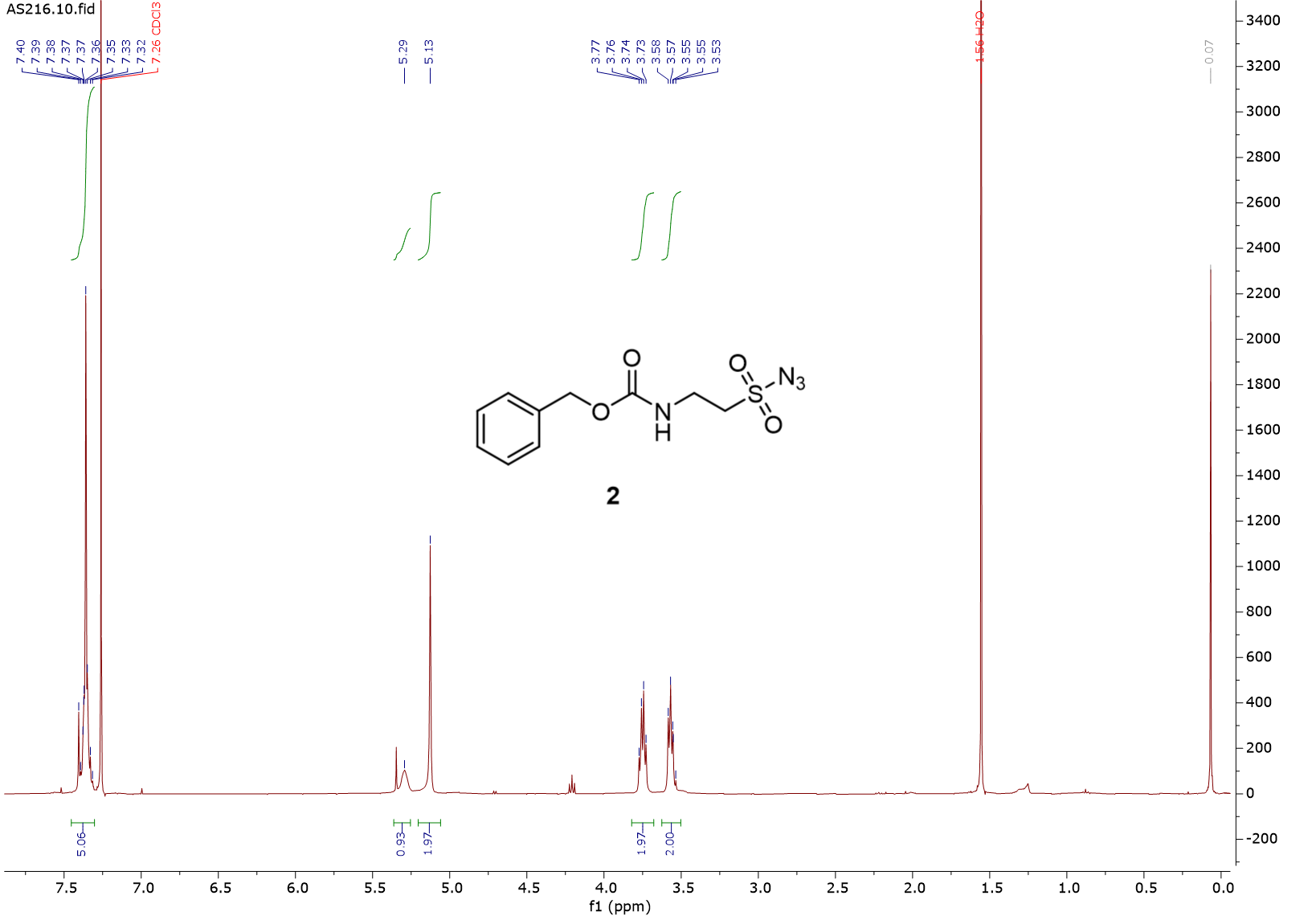


^1^HNMR of compound **3**

**
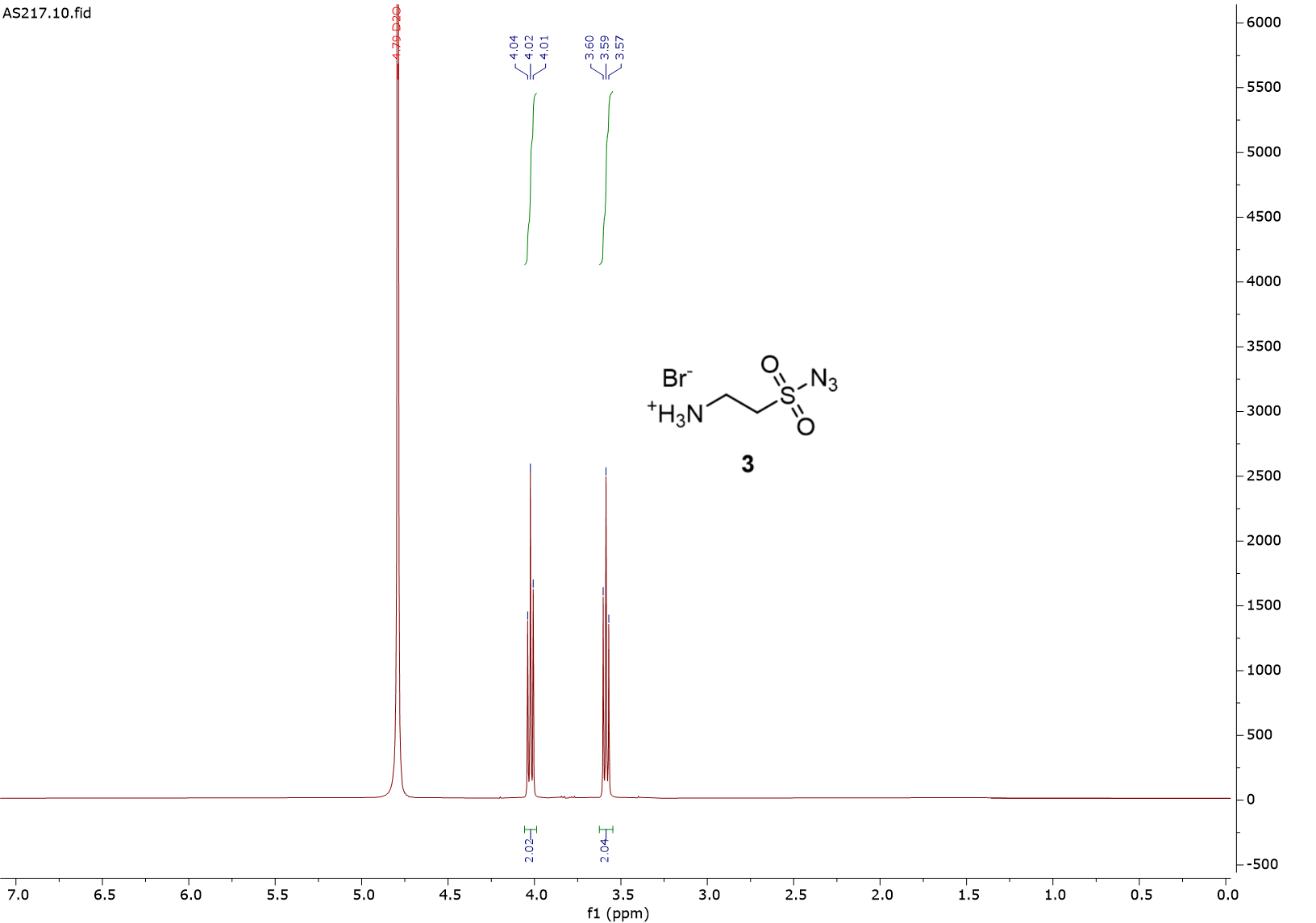
**

^13^CNMR of compound **3**

**
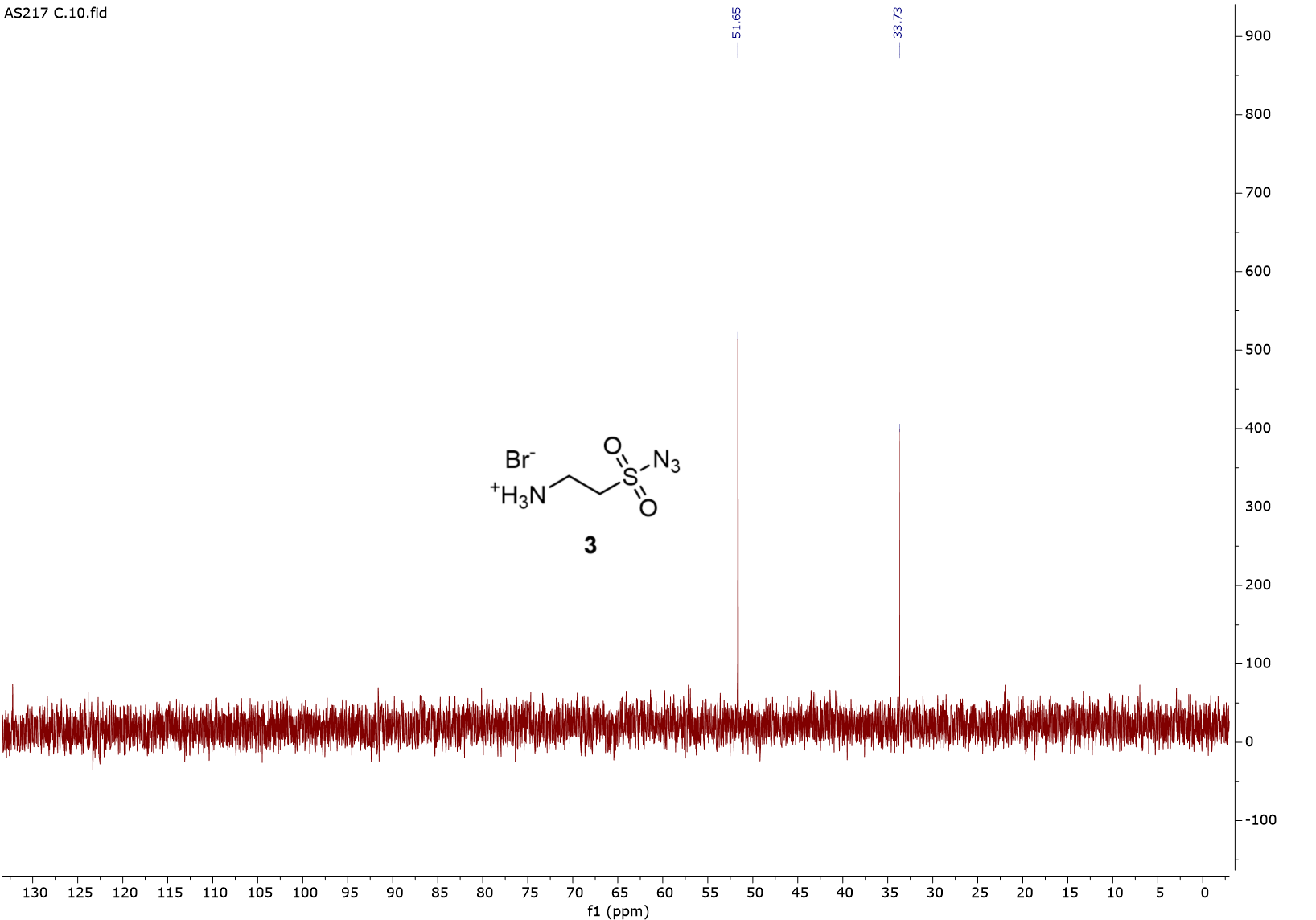
**

^1^HNMR of compound **S1**

**
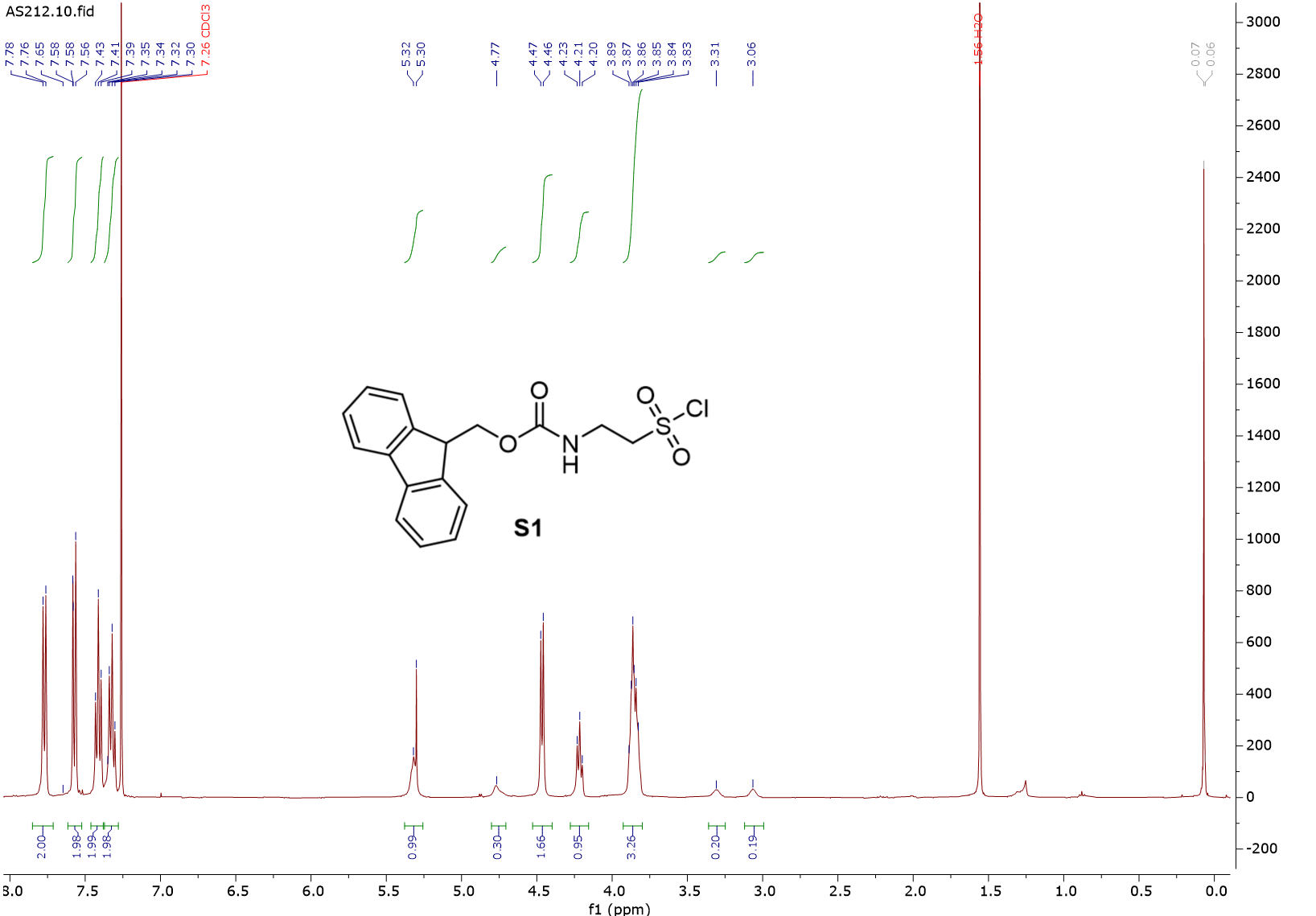
**

^13^CNMR of compound **S1**

**
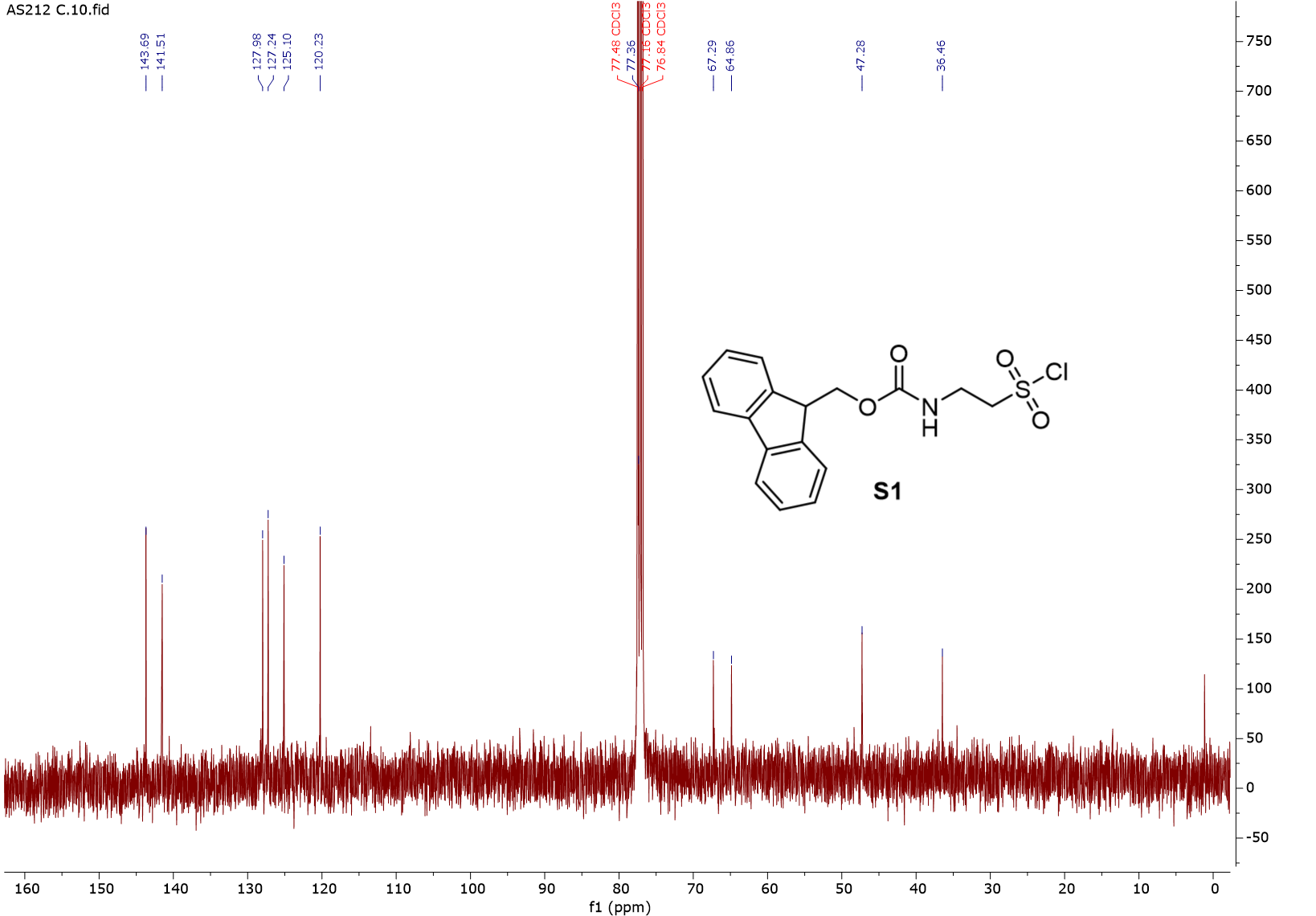
**

^1^HNMR of compound **3a**

**
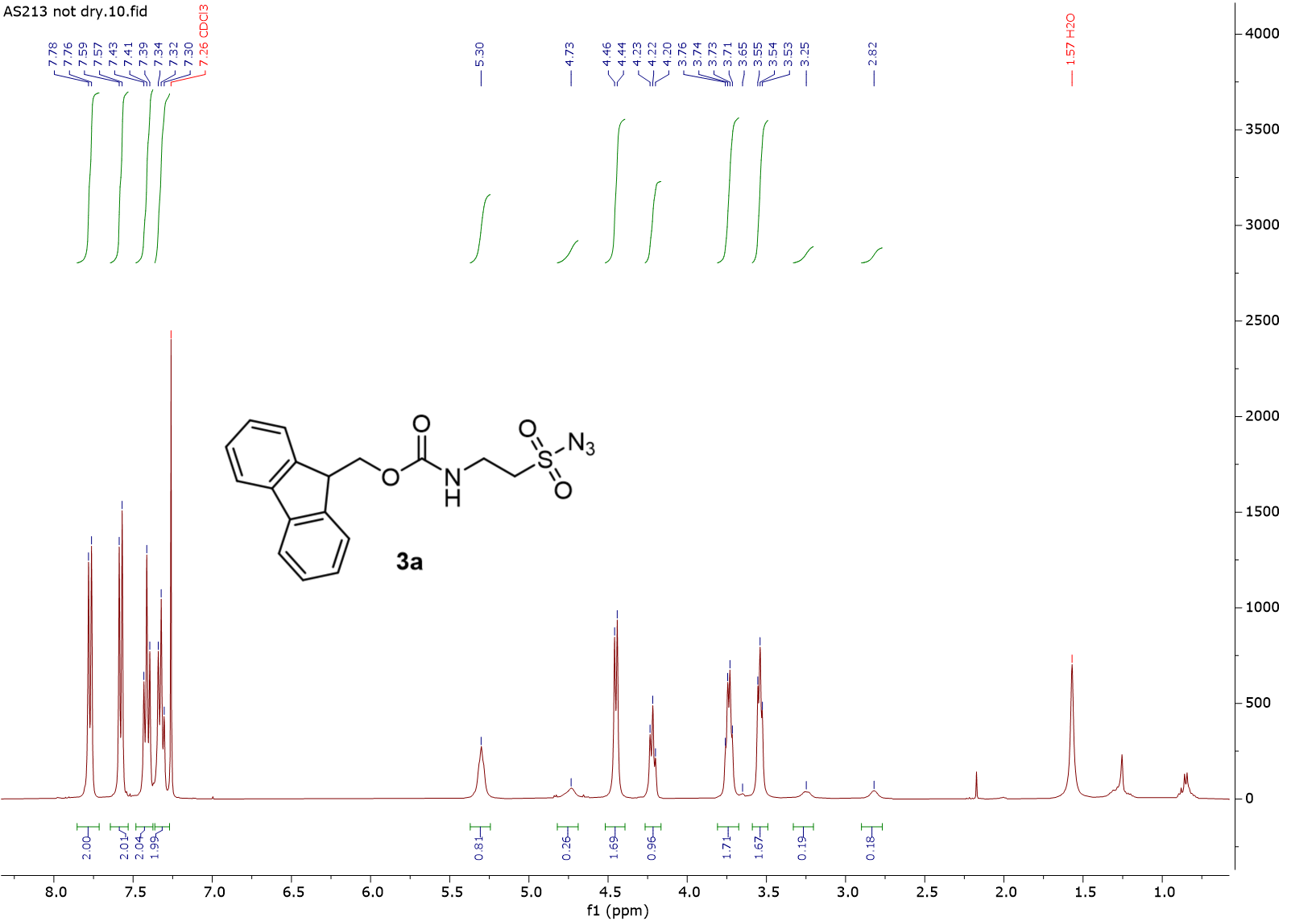
**

^13^CNMR of compound **3a**

**
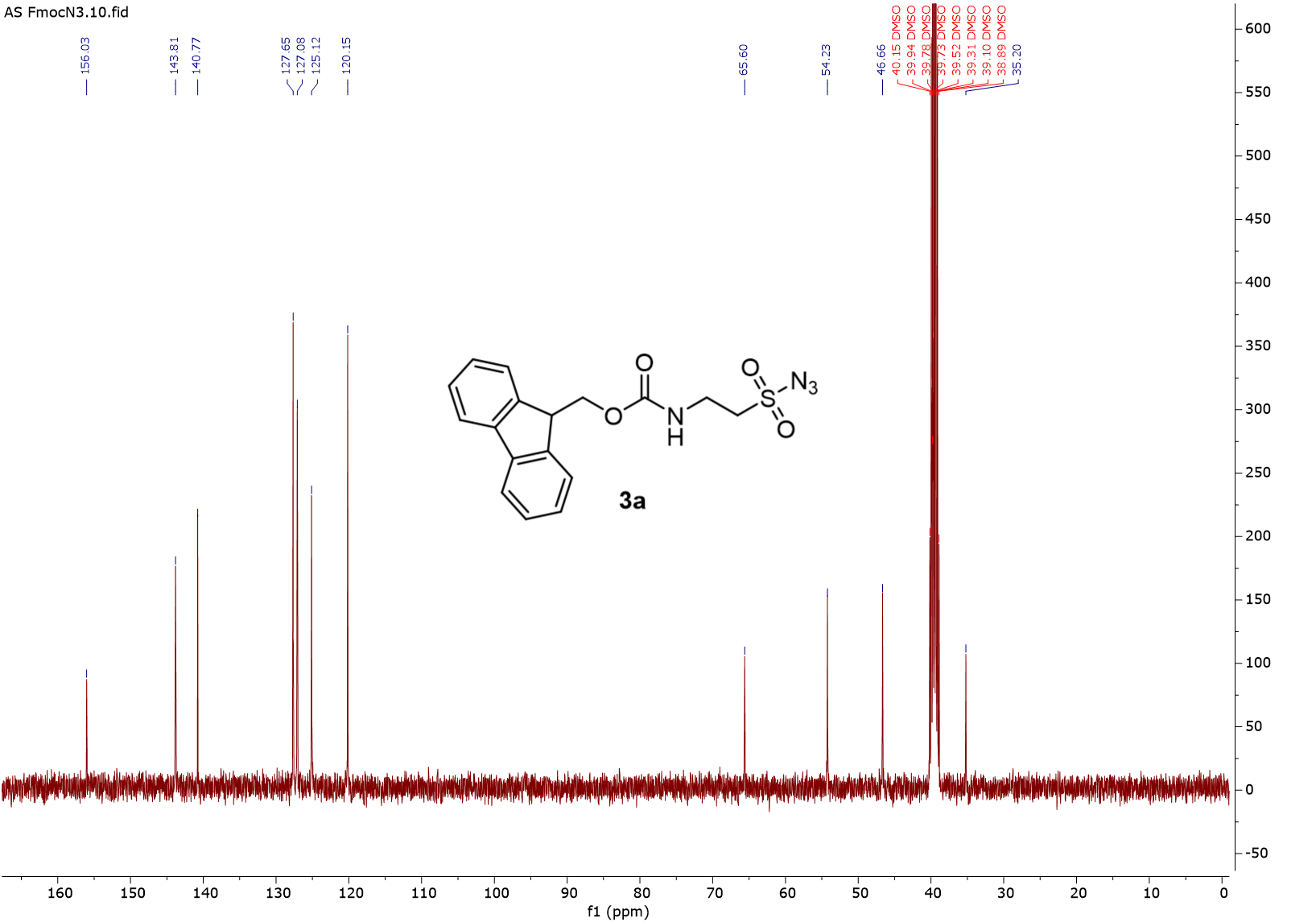
**

^1^HNMR of compound **3b**

**
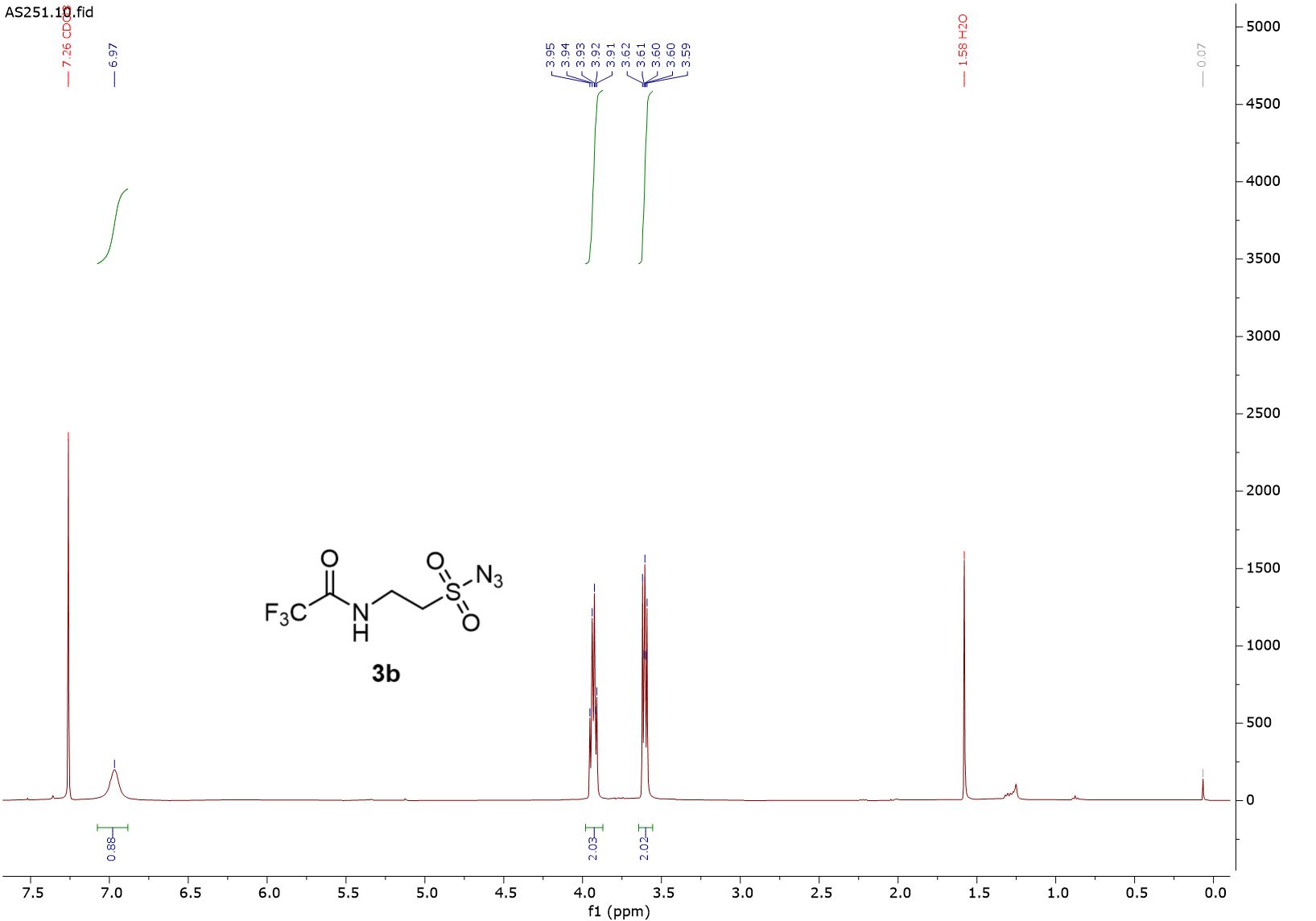
**

^13^CNMR of compound **3b**

**
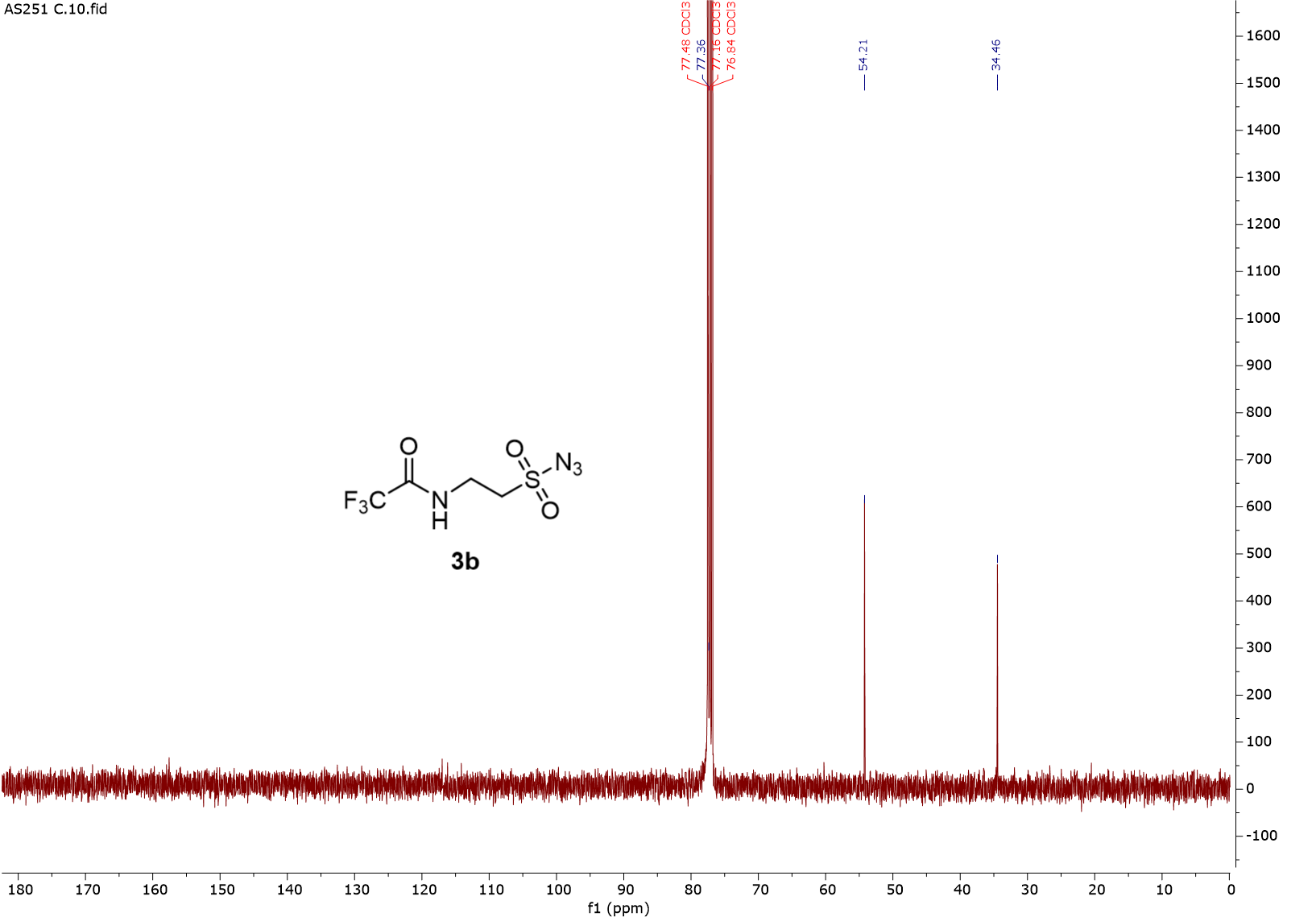
**

^19^FNMR of compound **3b**

**
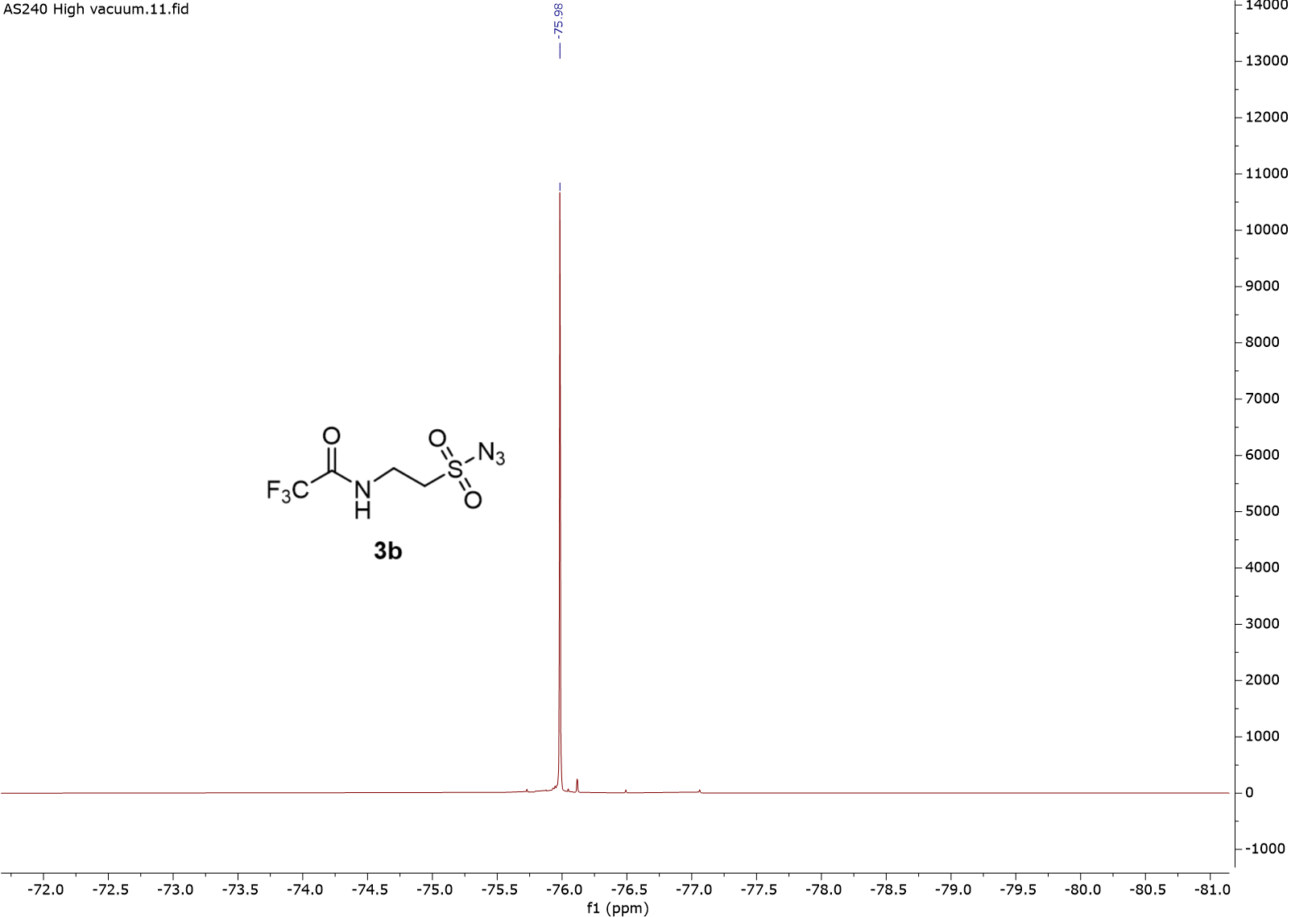
**

^1^HNMR of compound **S2**

**
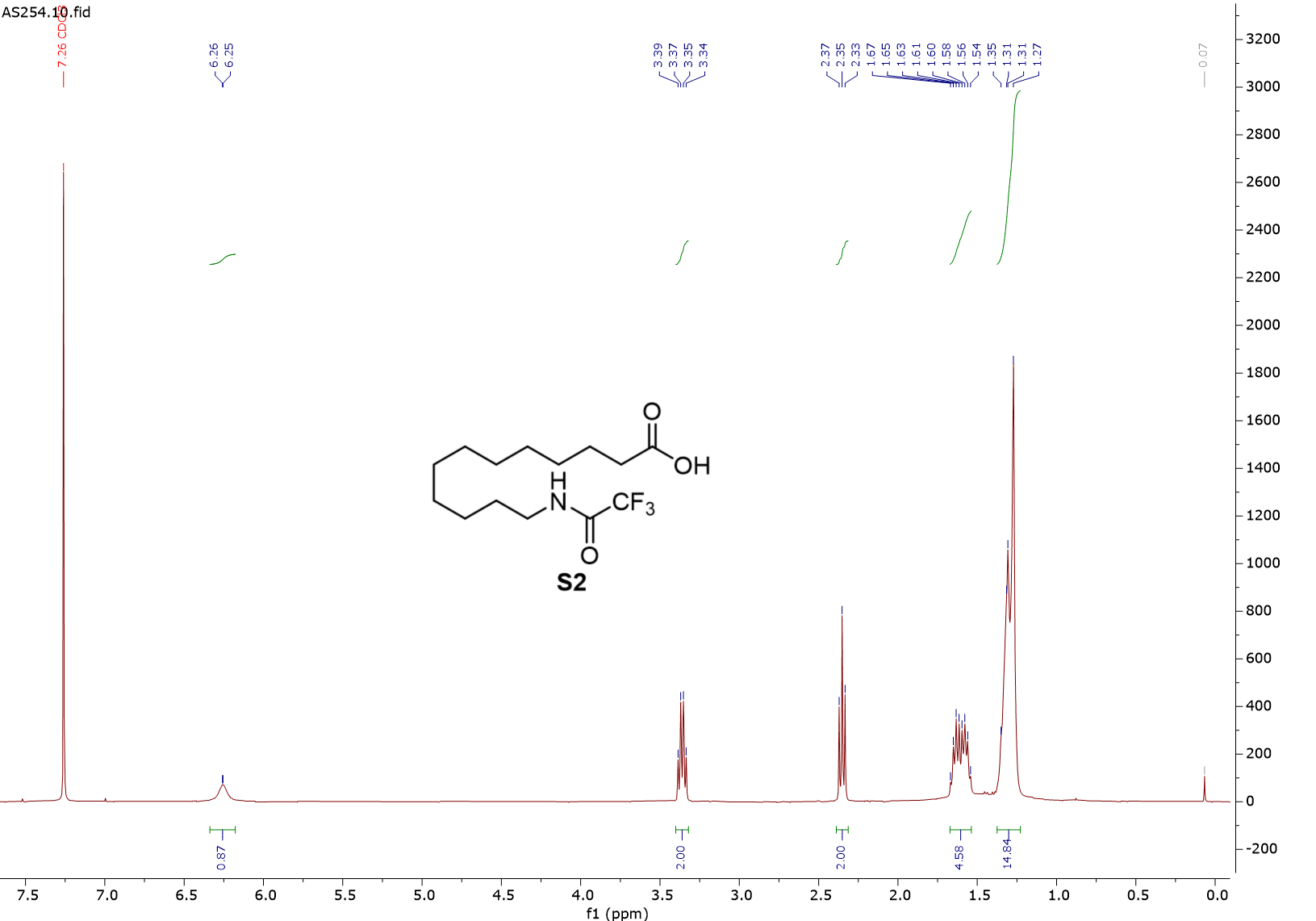
**

^13^CNMR of compound **S2**

**
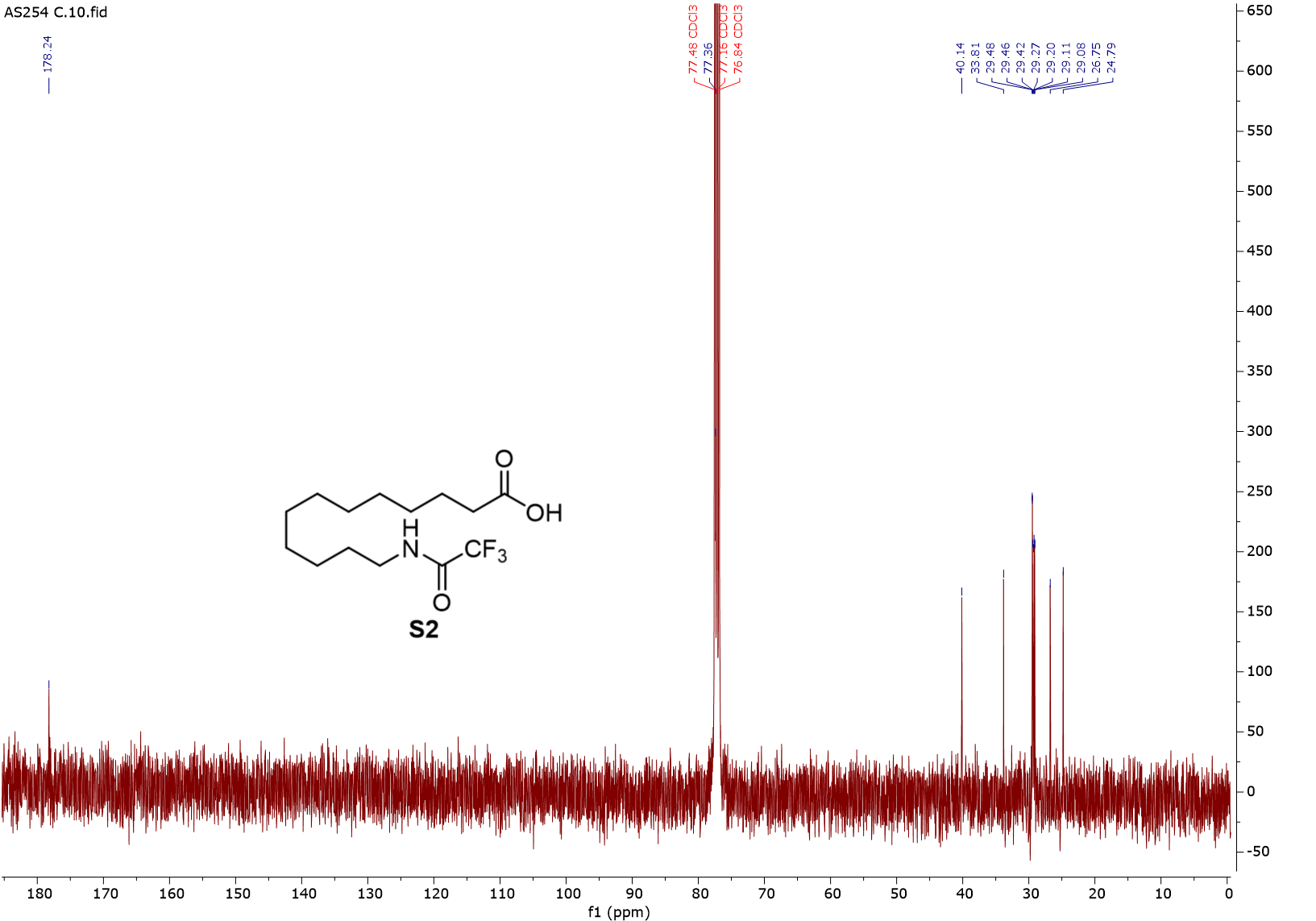
**

^19^FNMR of compound **S2**

**
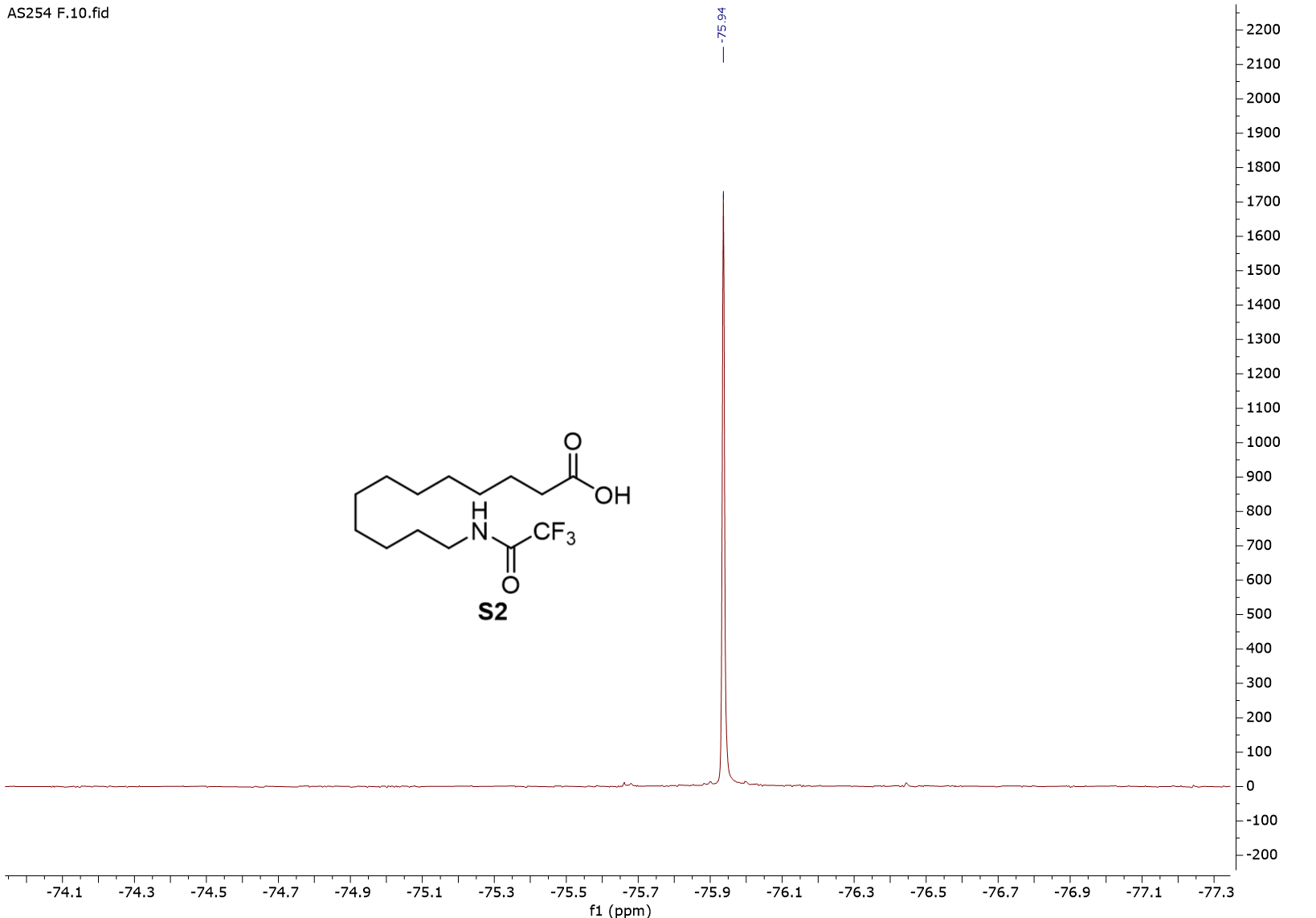
**

^1^HNMR of compound **3c**

**
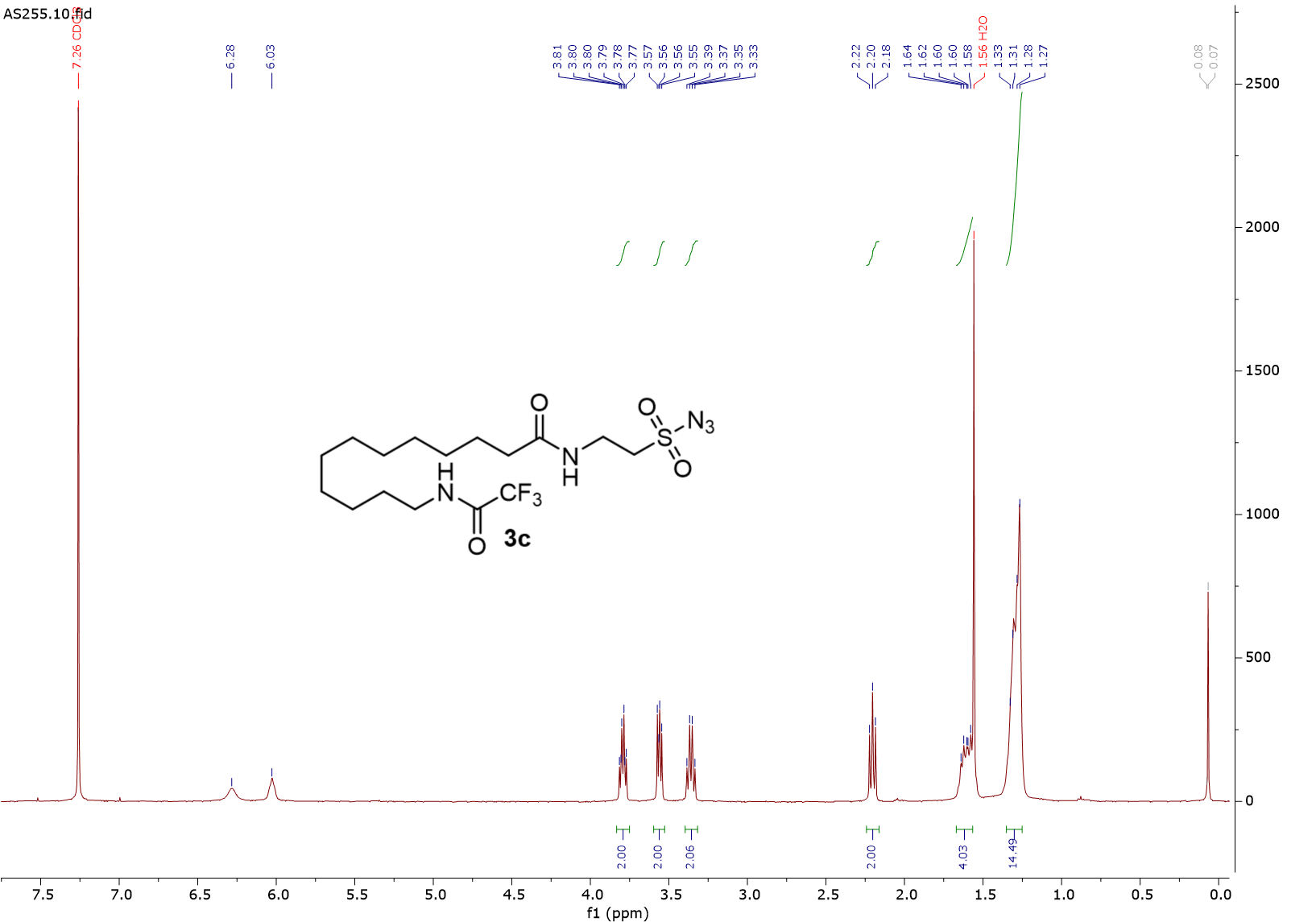
**

^13^CNMR of compound **3c**

**
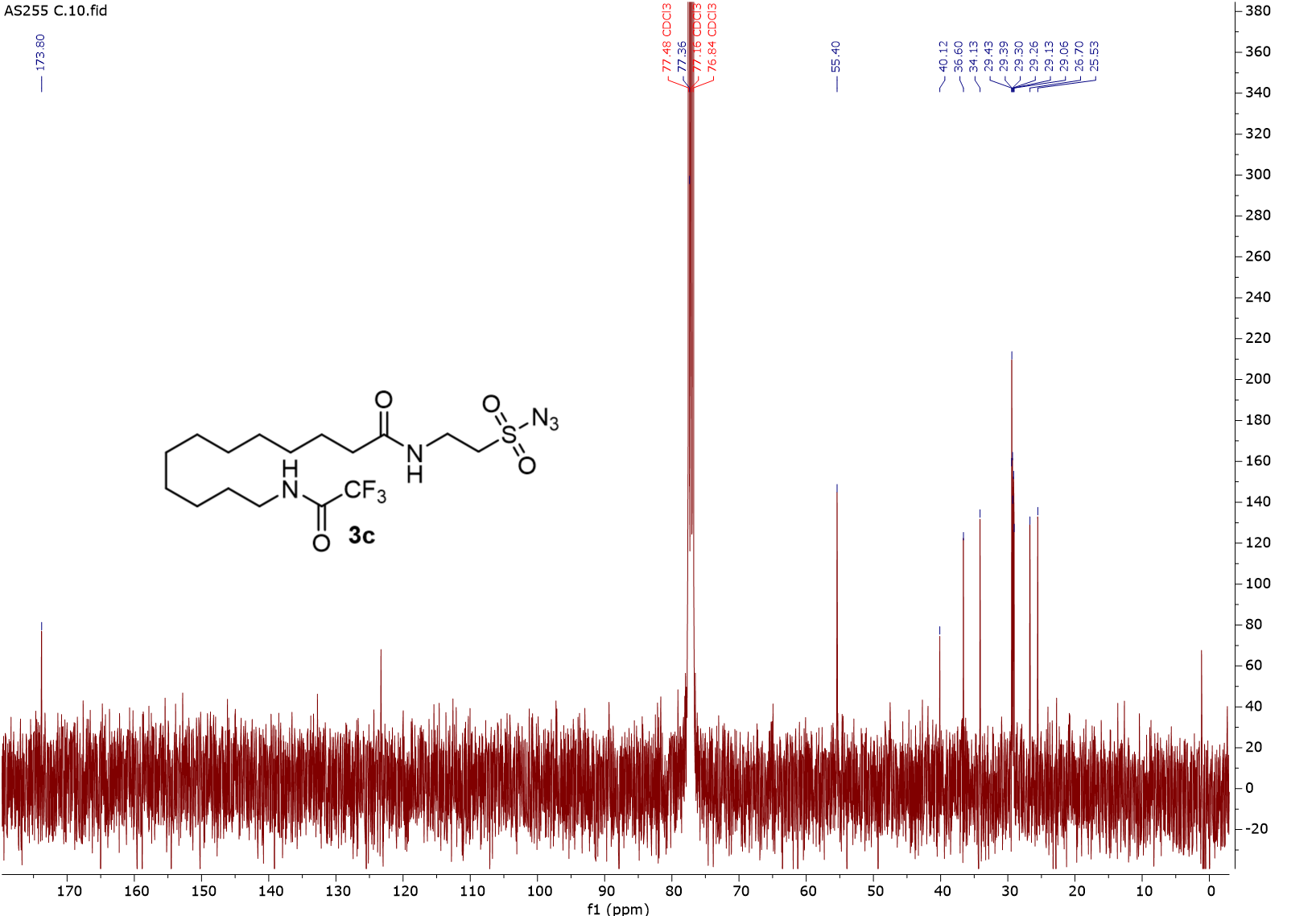
**

^19^FNMR of compound **3c**

**
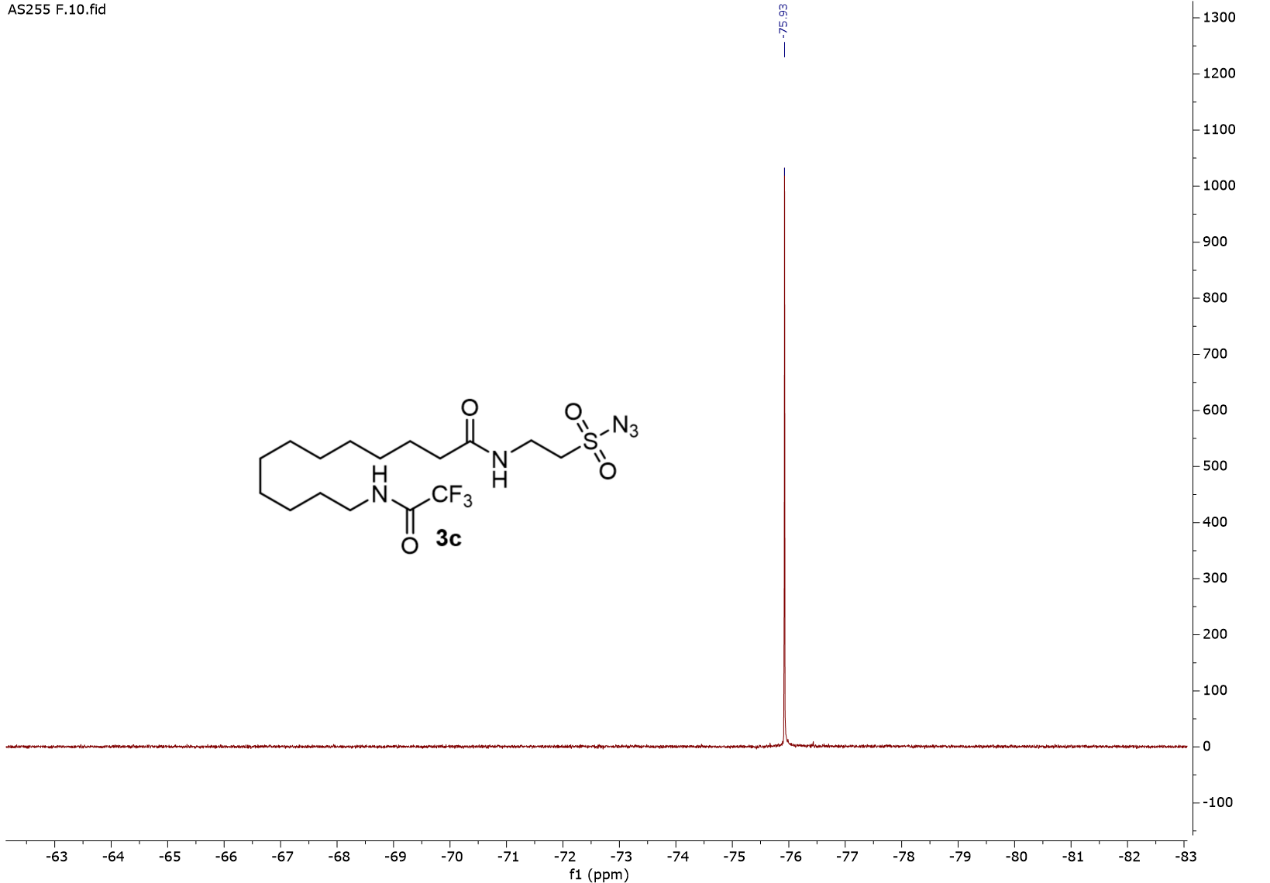
**

^1^HNMR of compound **S3**

**
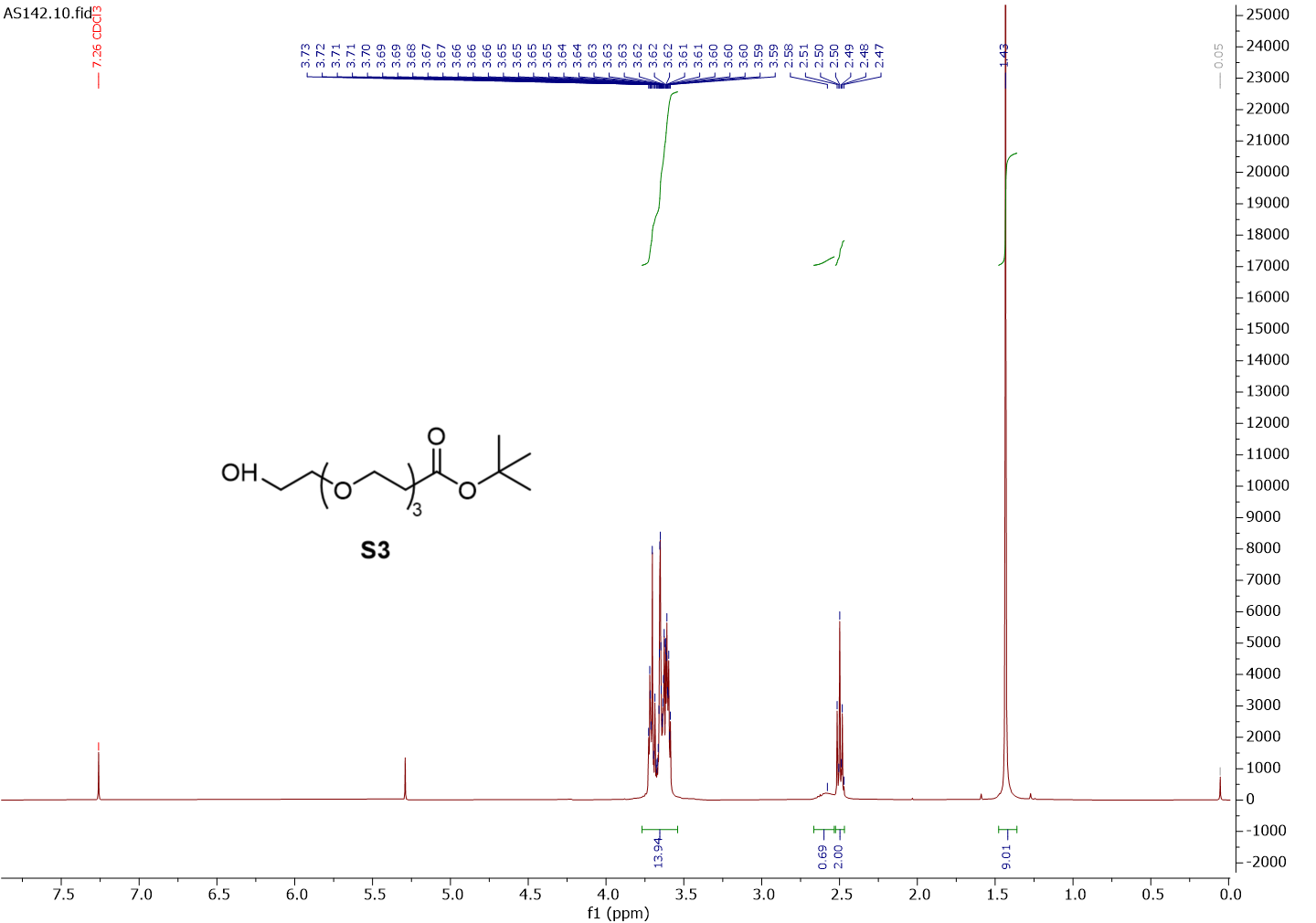
**

^13^CNMR of compound **S3**

**
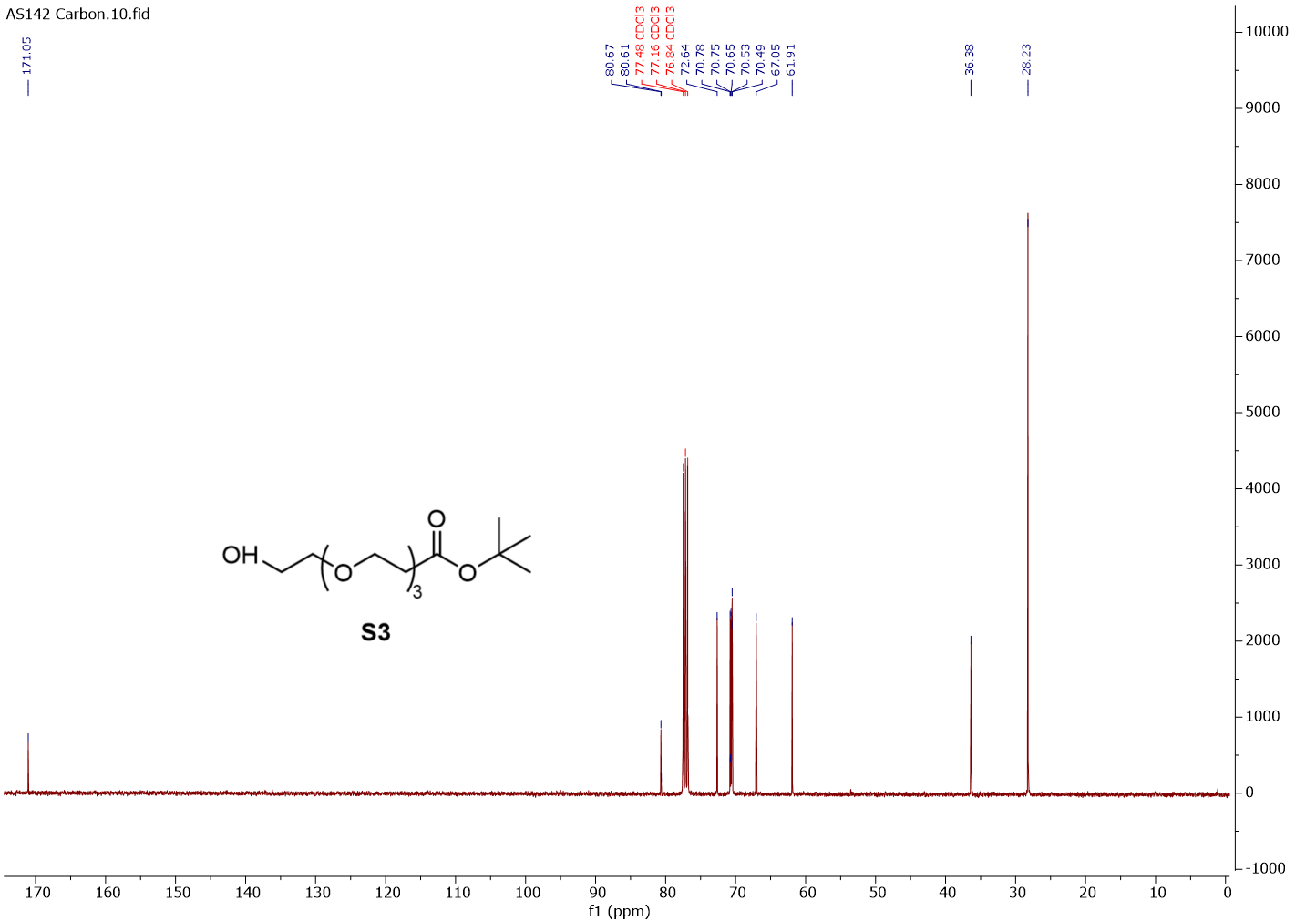
**

^1^HNMR of compound **S4**

**
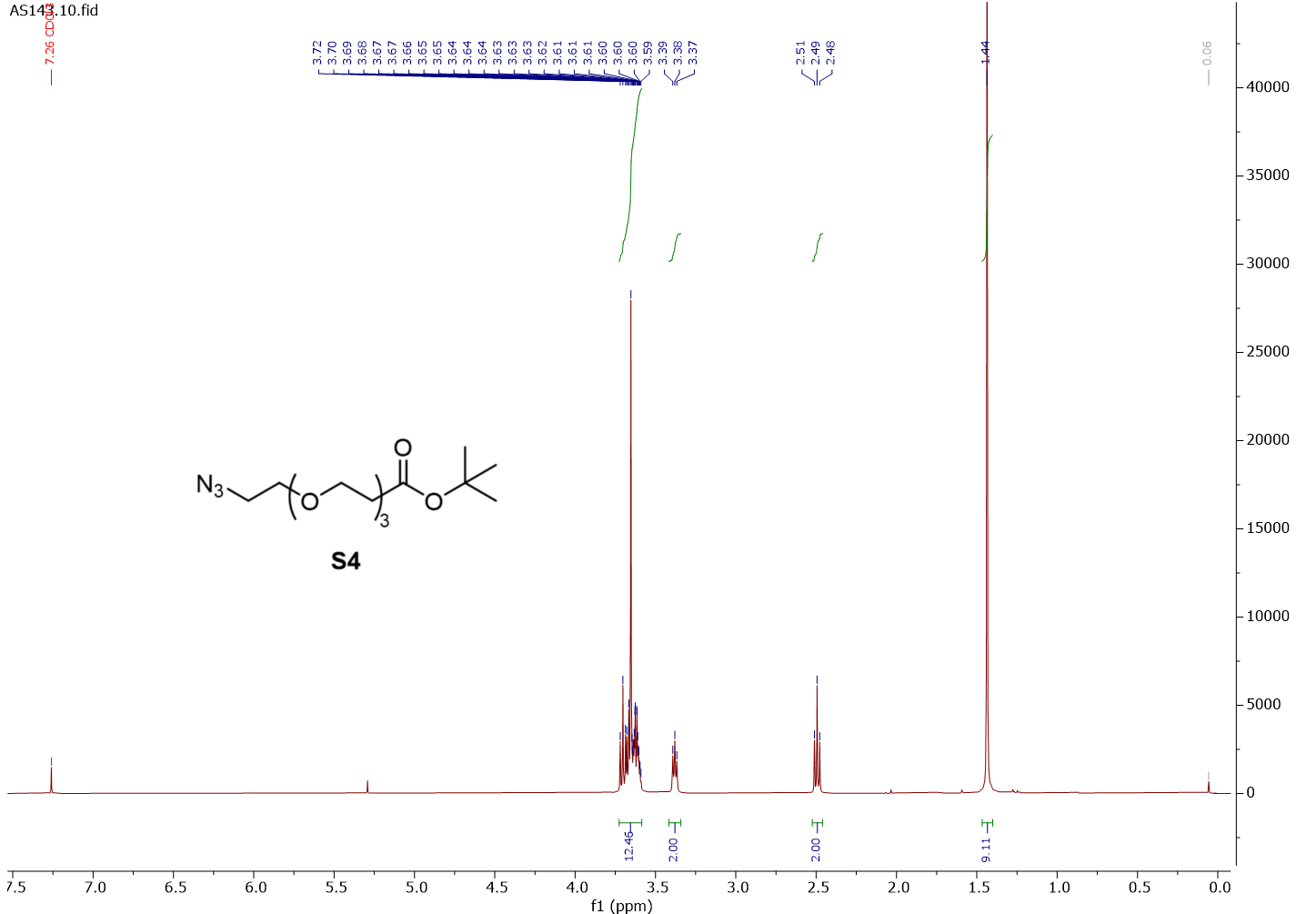
**

^13^CNMR of compound **S4**

**
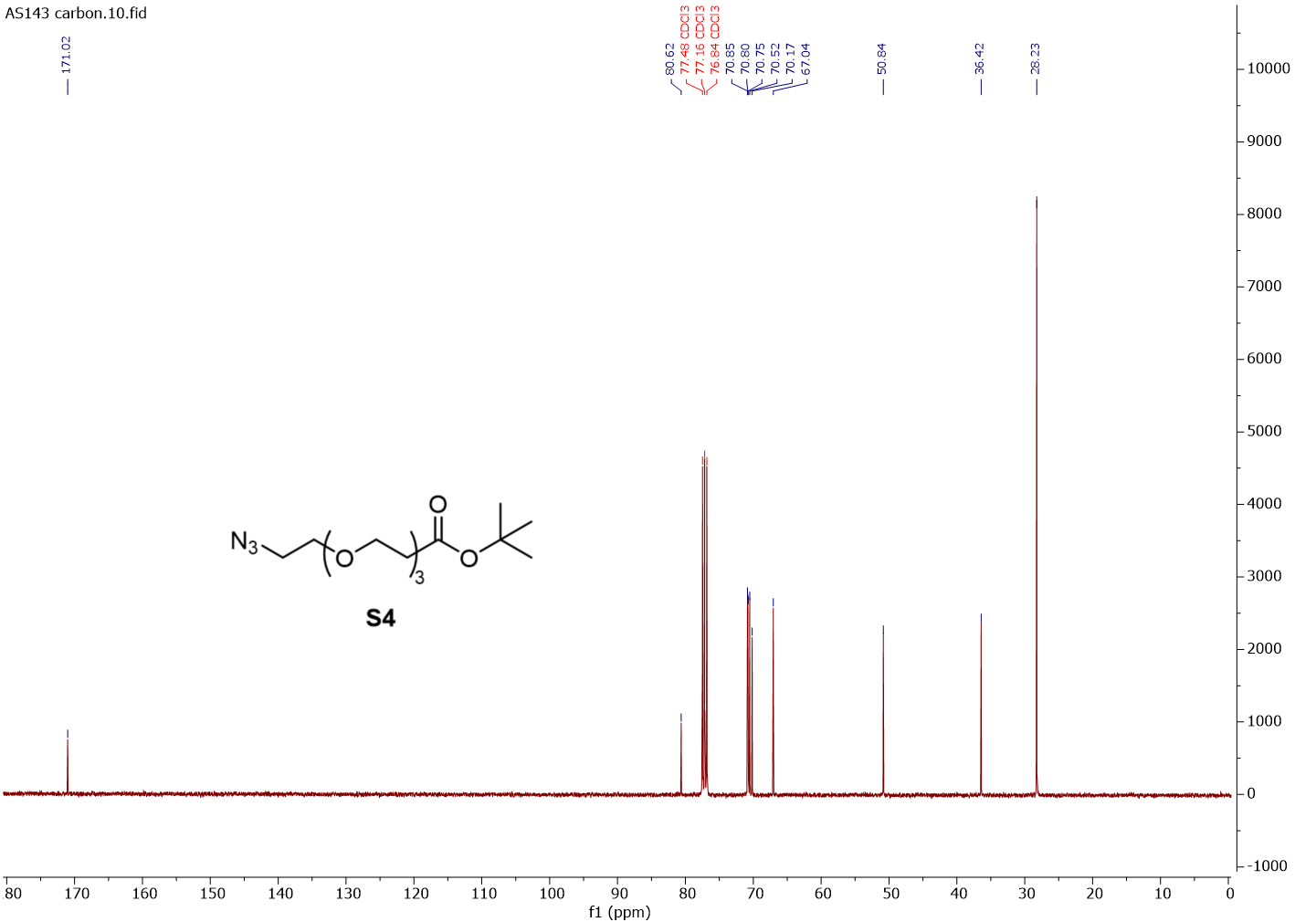
**

^1^HNMR of compound **S5**

**
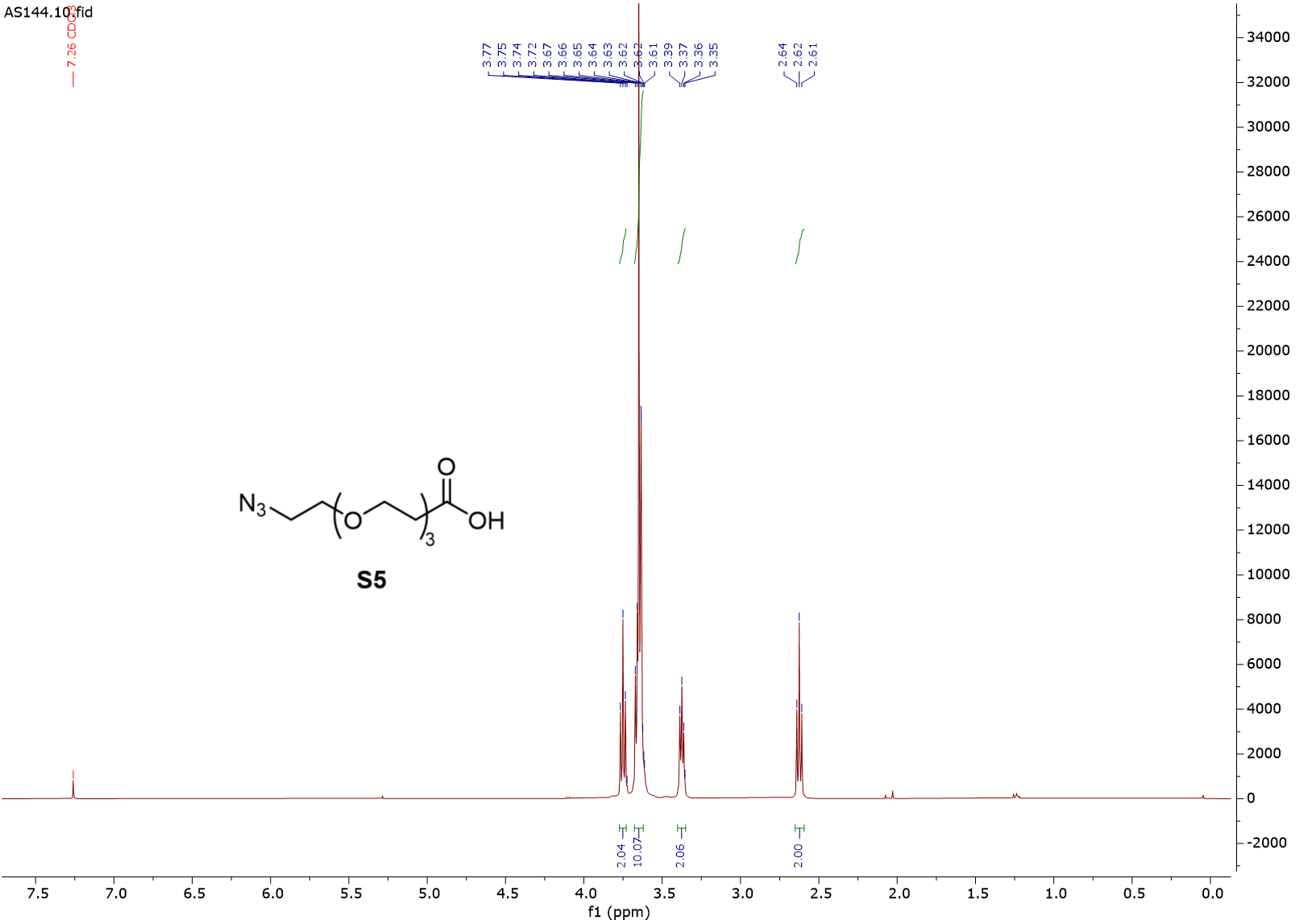
**

^13^CNMR of compound **S5**

**
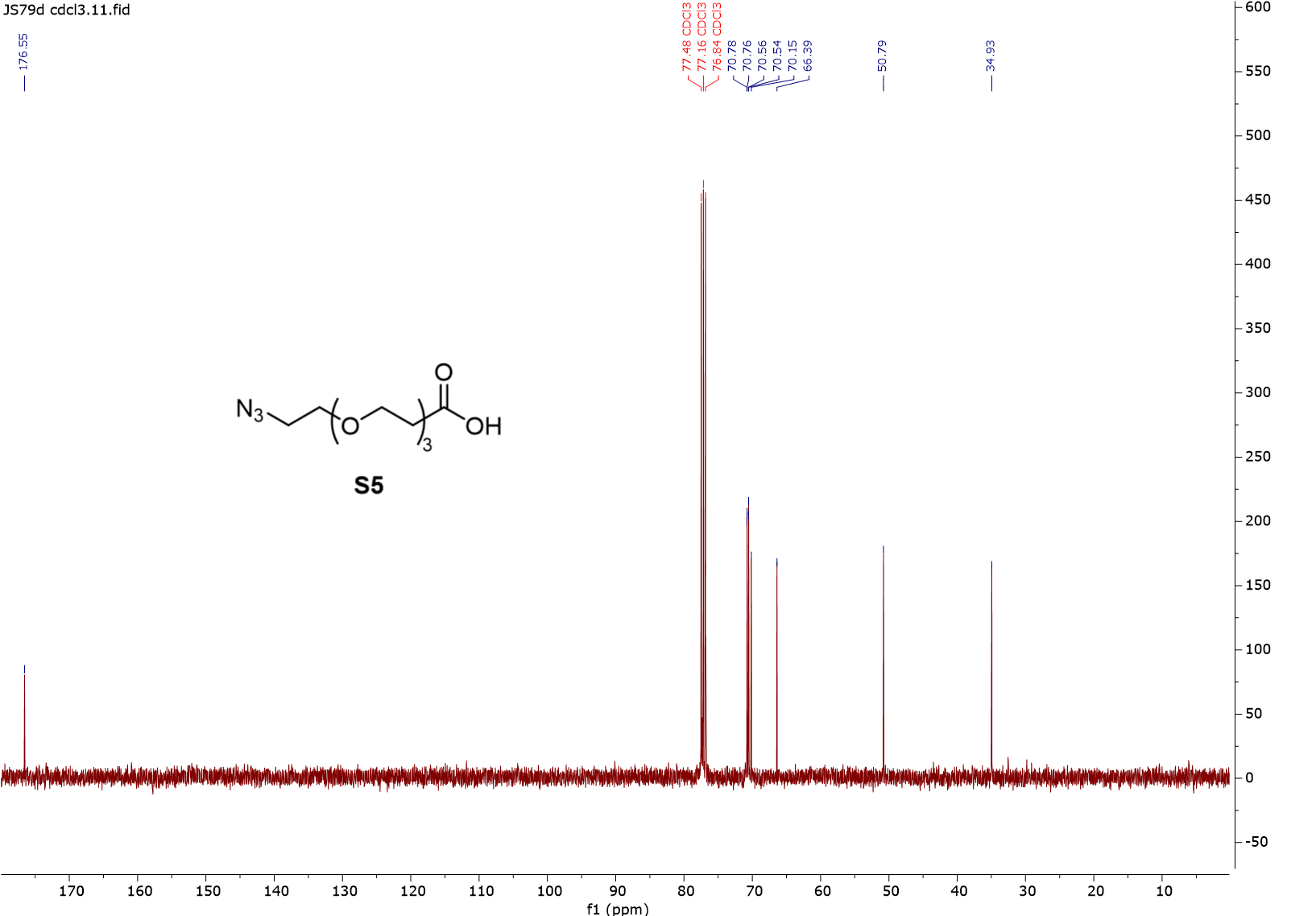
**

^1^HNMR of compound **3d**

**
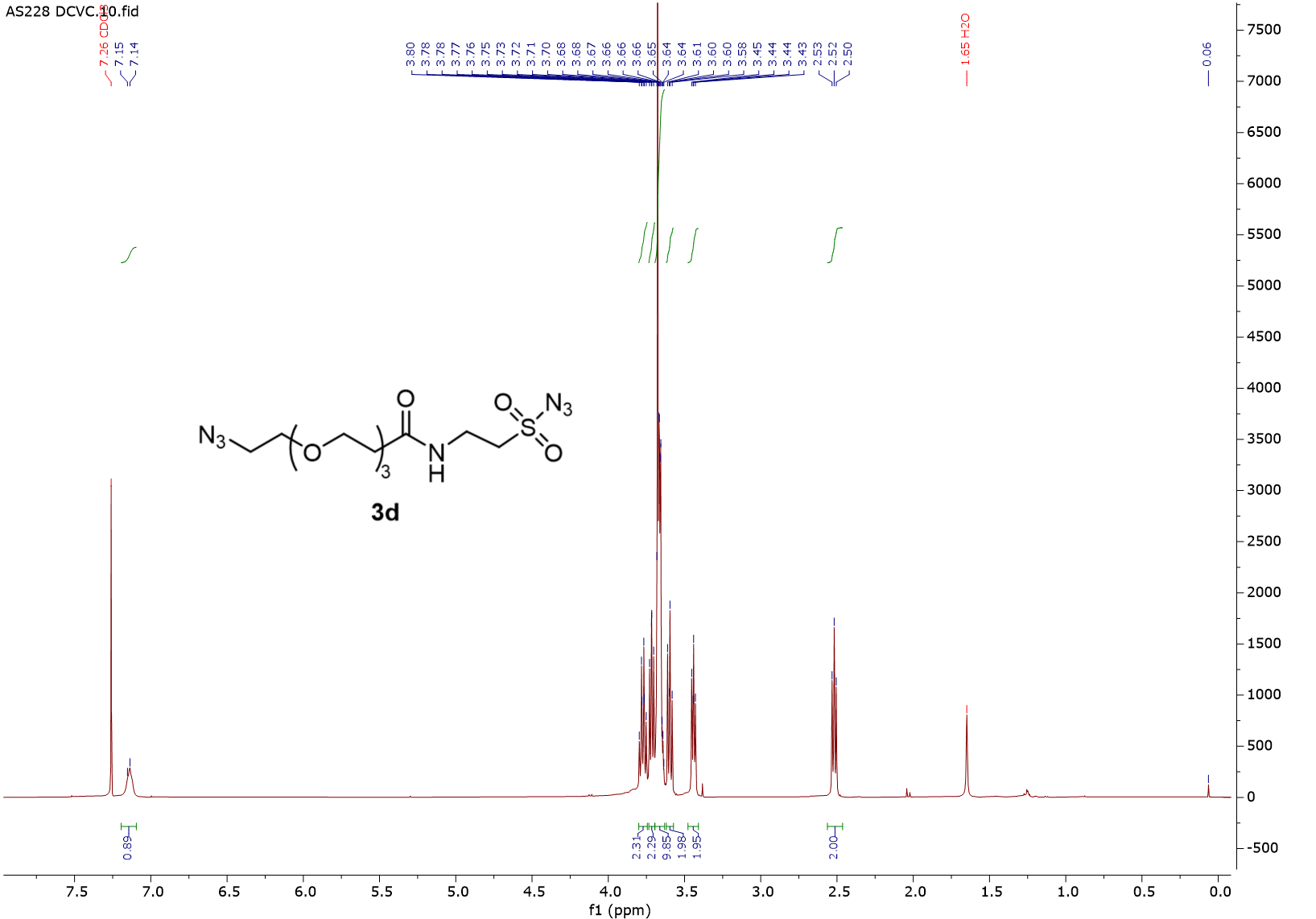
**

^13^CNMR of compound **3d**

**
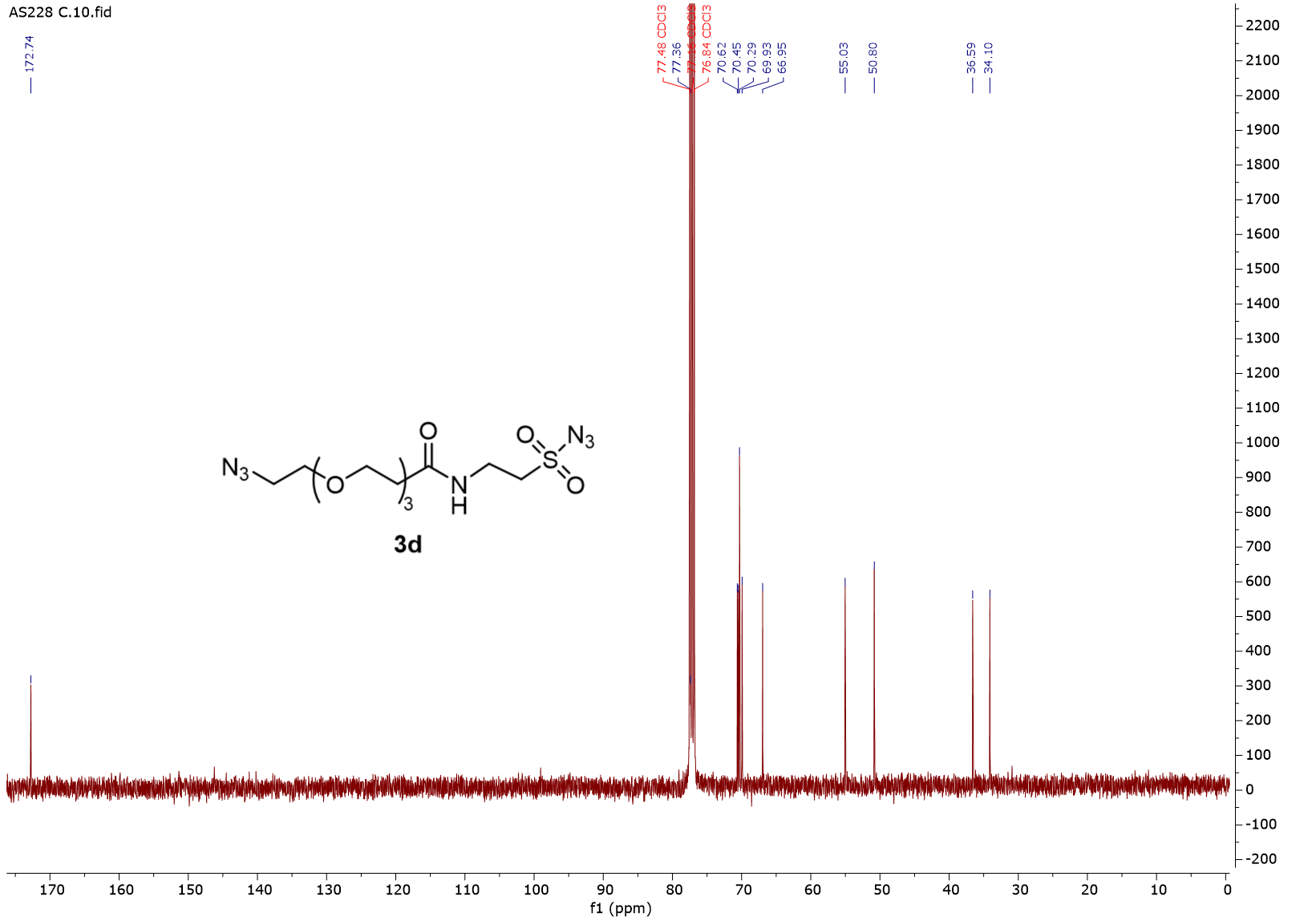
**

^1^HNMR of compound **3e**

**
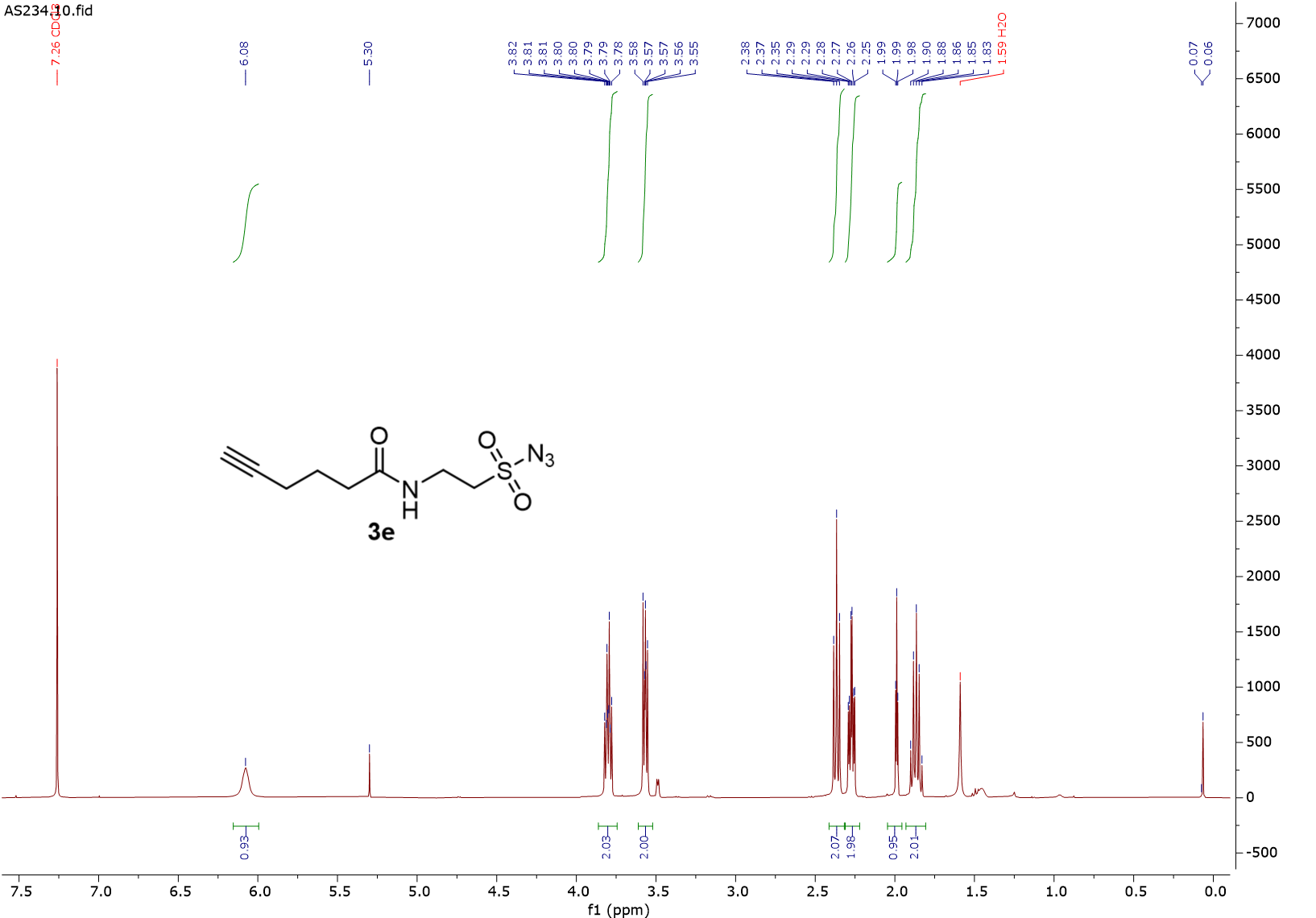
**

^13^CNMR of compound **3e**

**
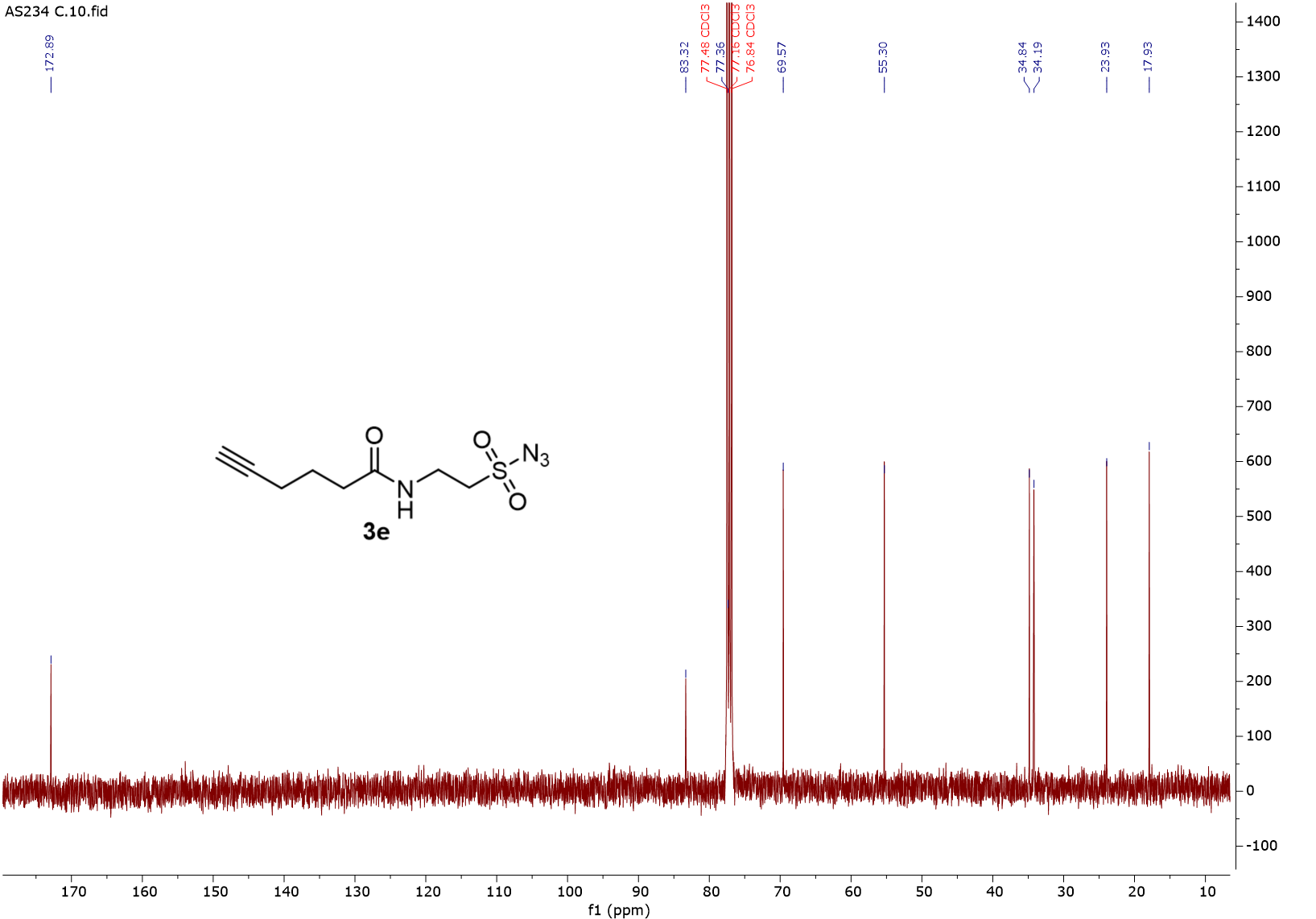
**

^1^HNMR of compound **S6**

**
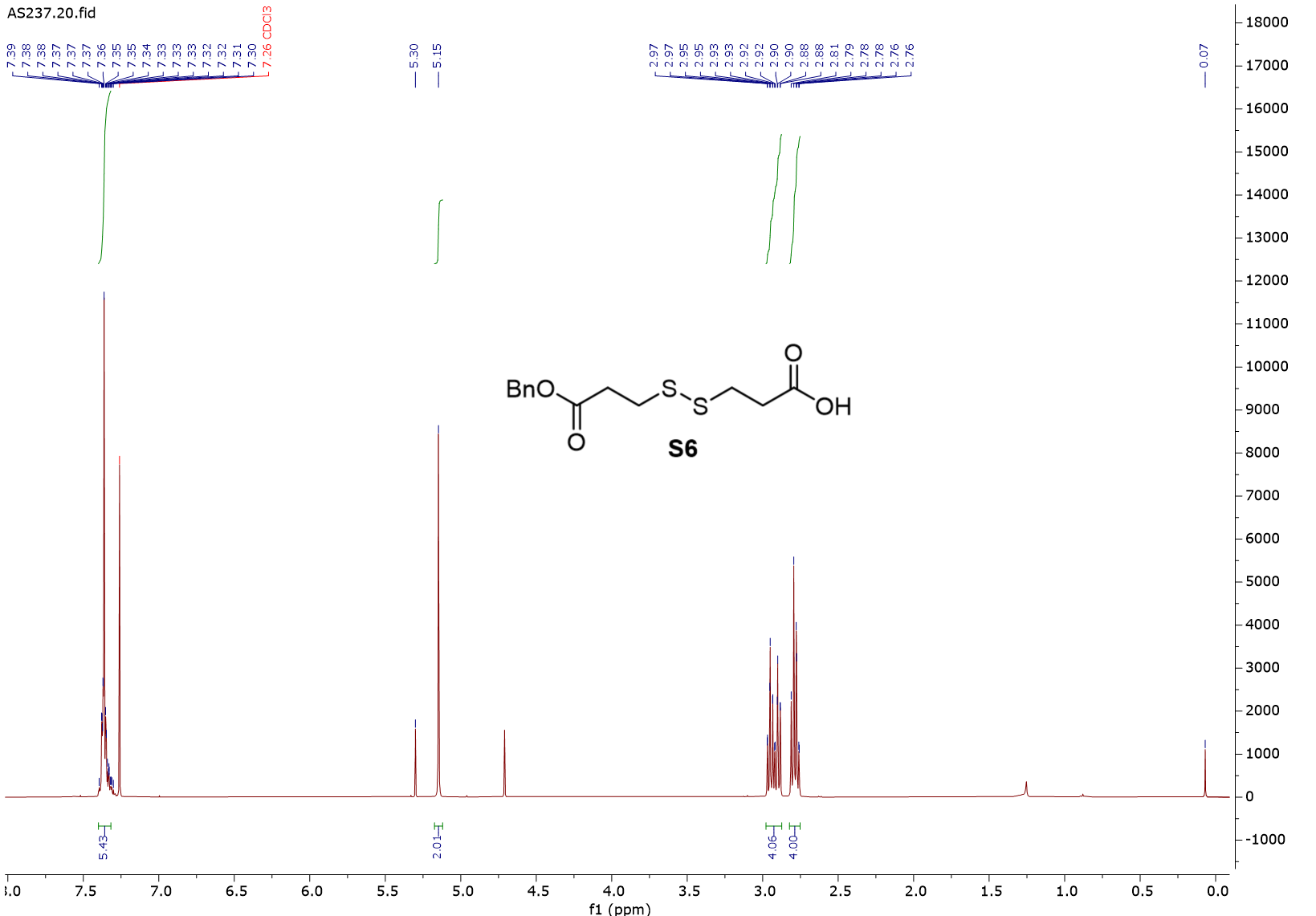
**

^13^CNMR of compound **S6**

**
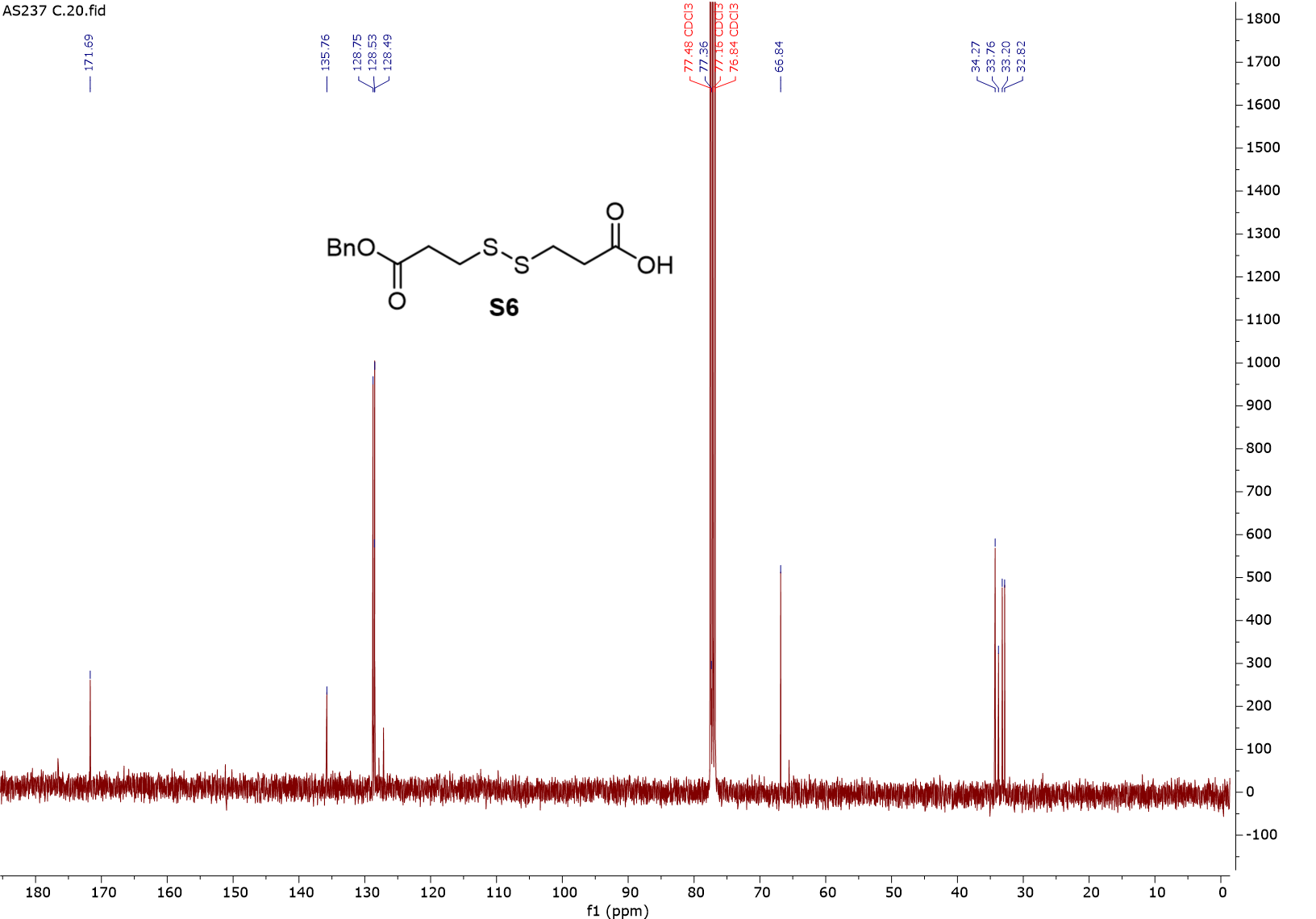
**

^1^HNMR of compound **3f**

**
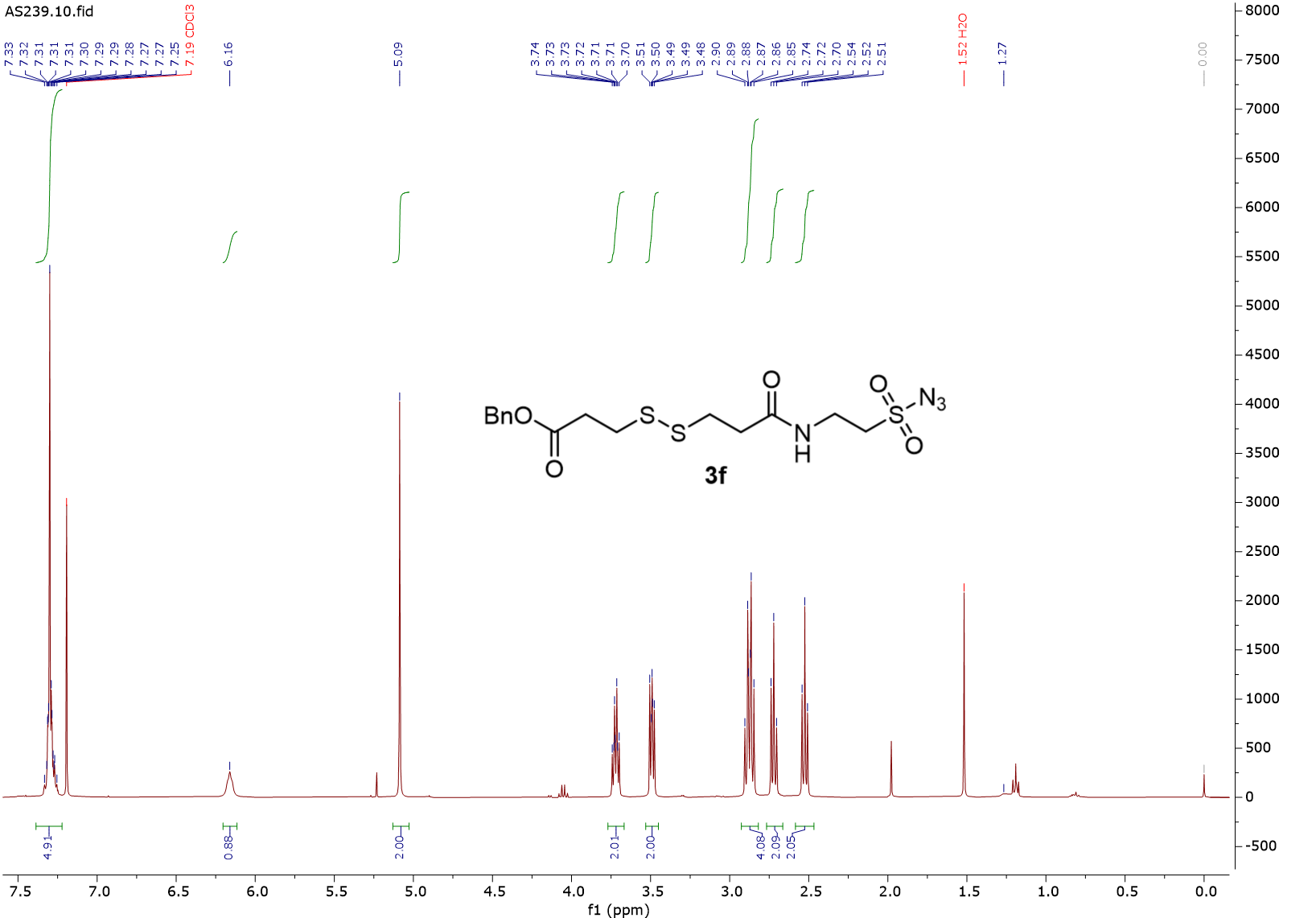
**

^13^CNMR of compound **3f**

**
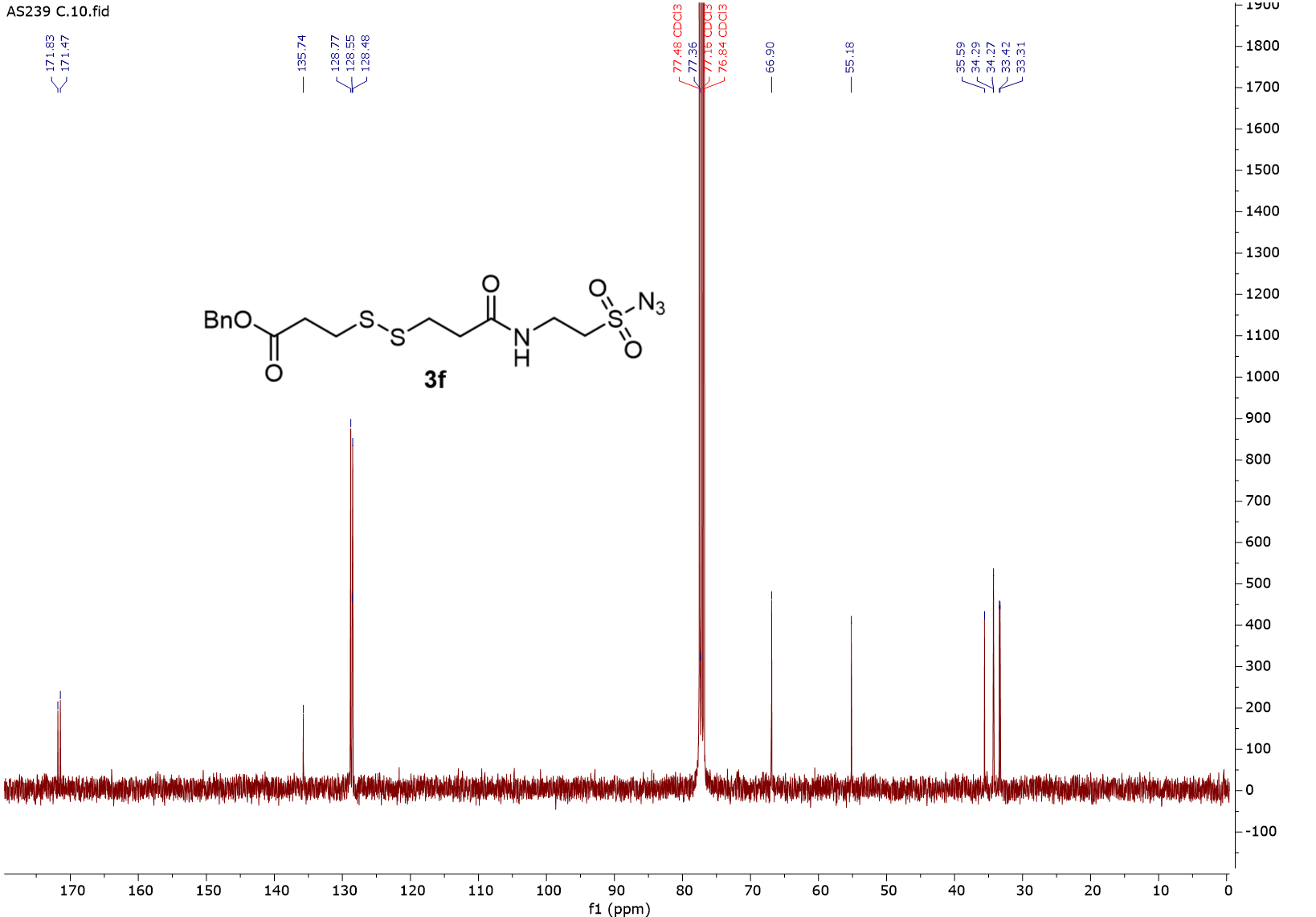
**

^1^HNMR of compound **S7**

**
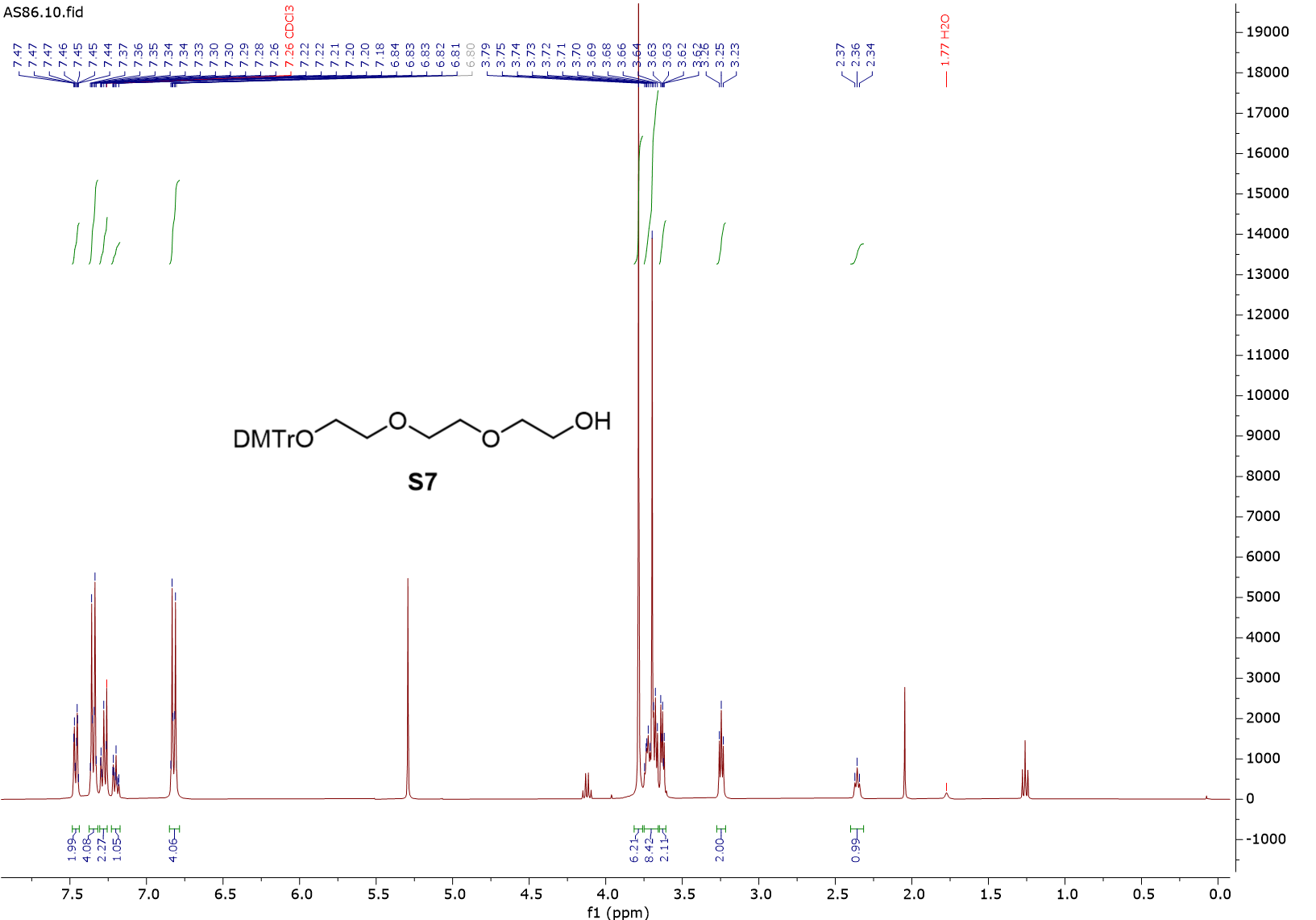
**

^13^CNMR of compound **S7**

**
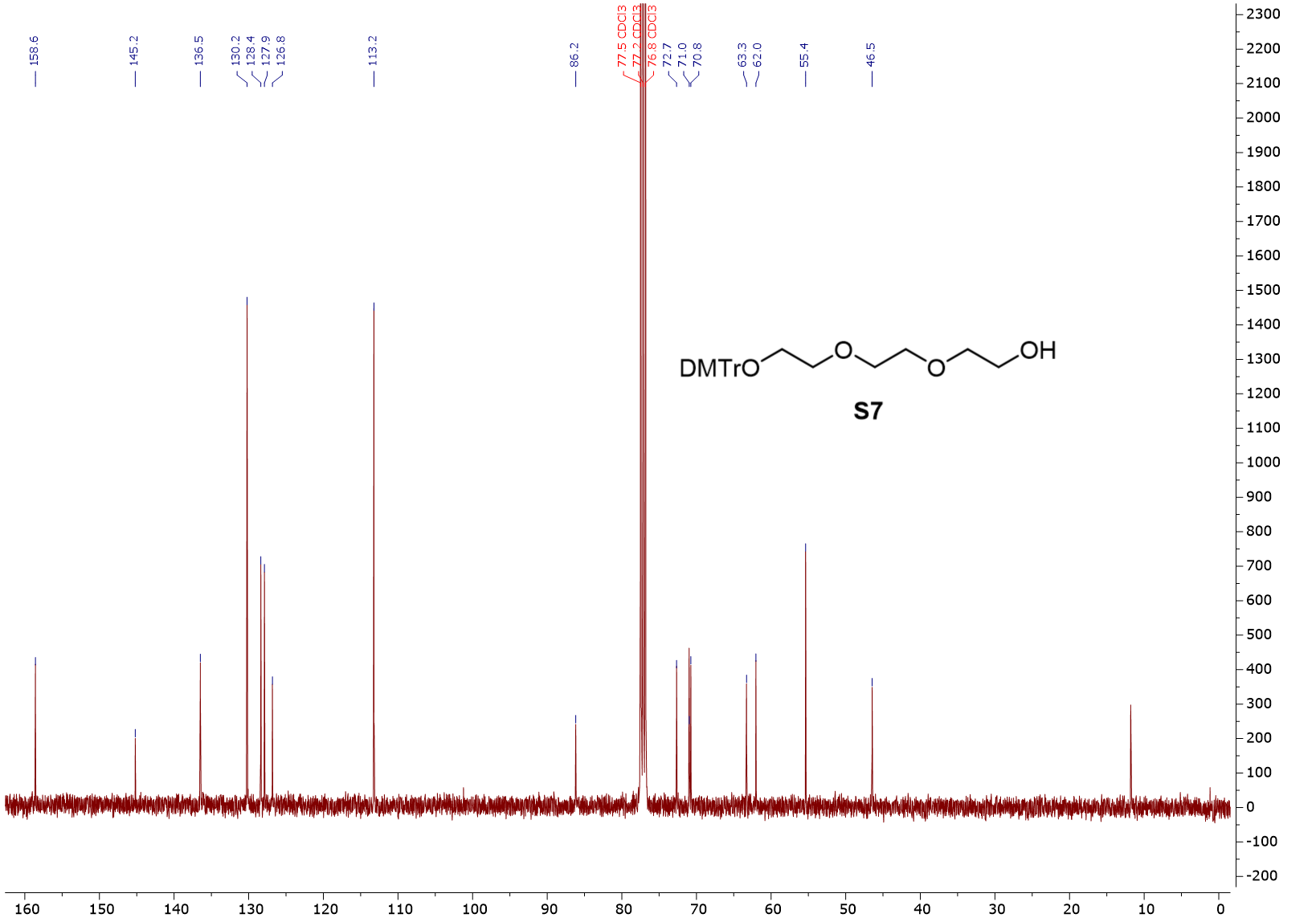
**

^1^HNMR of compound **S8**

**
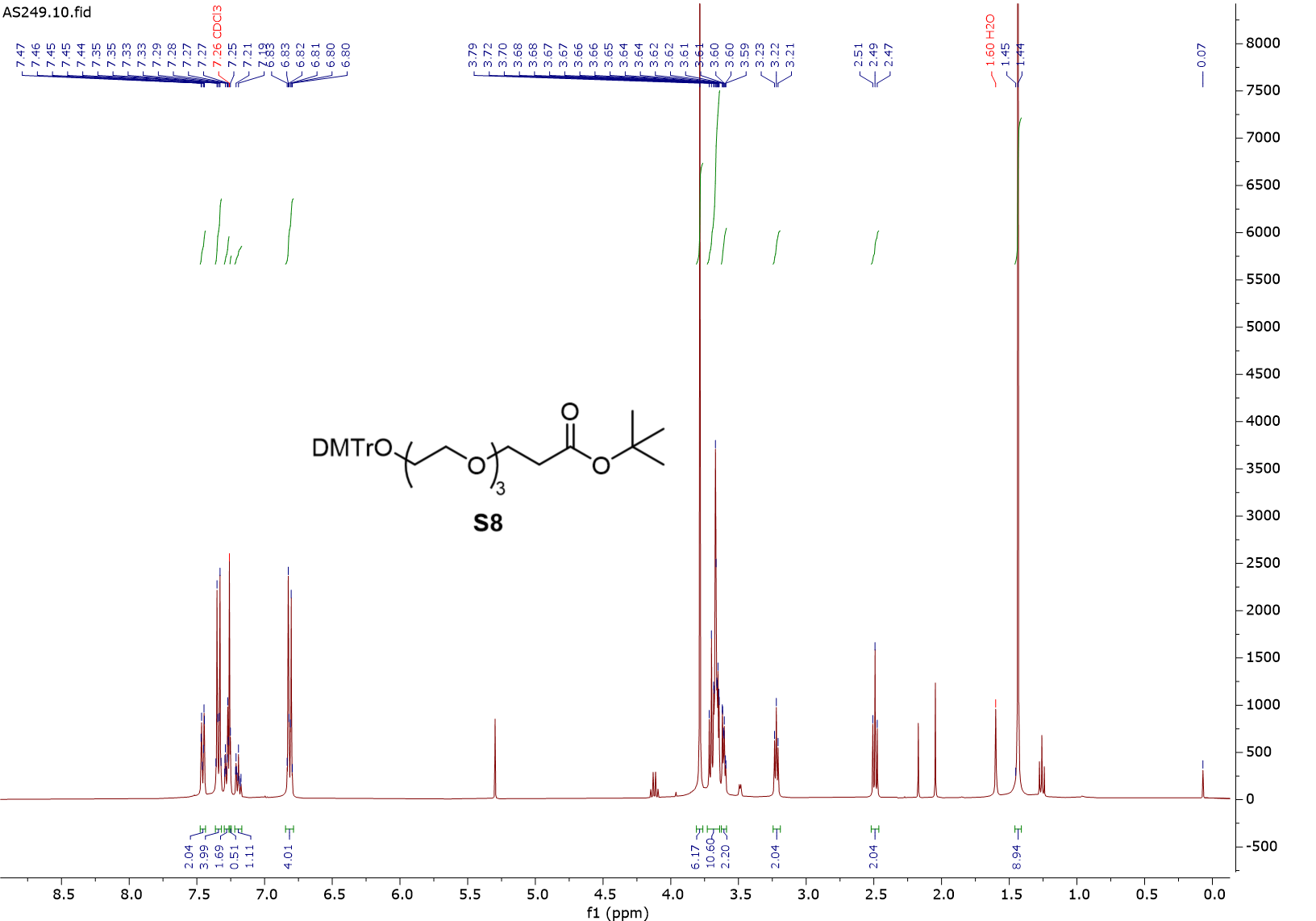
**

^13^CNMR of compound **S8**

**
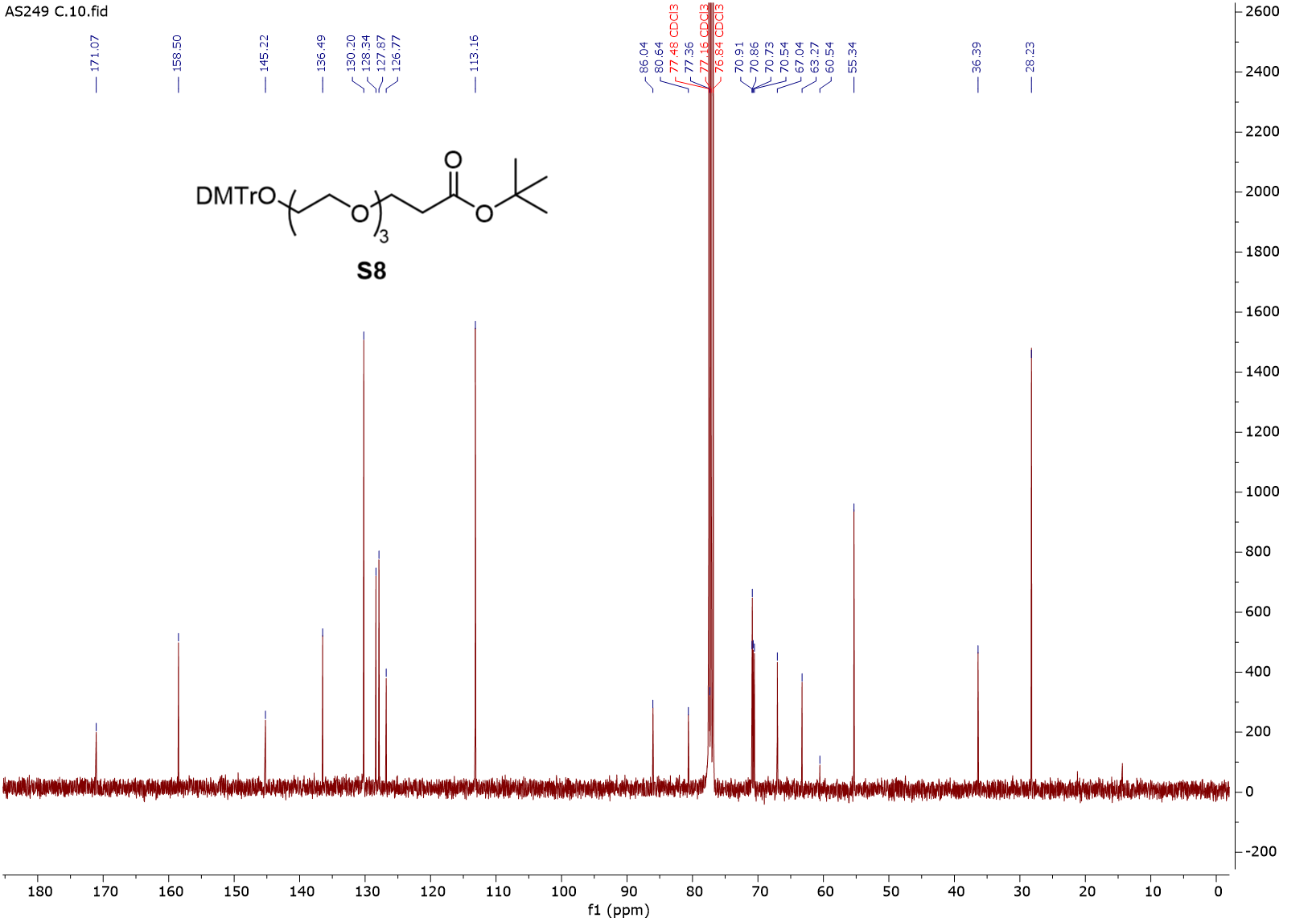
**

^1^HNMR of compound **S9**

**
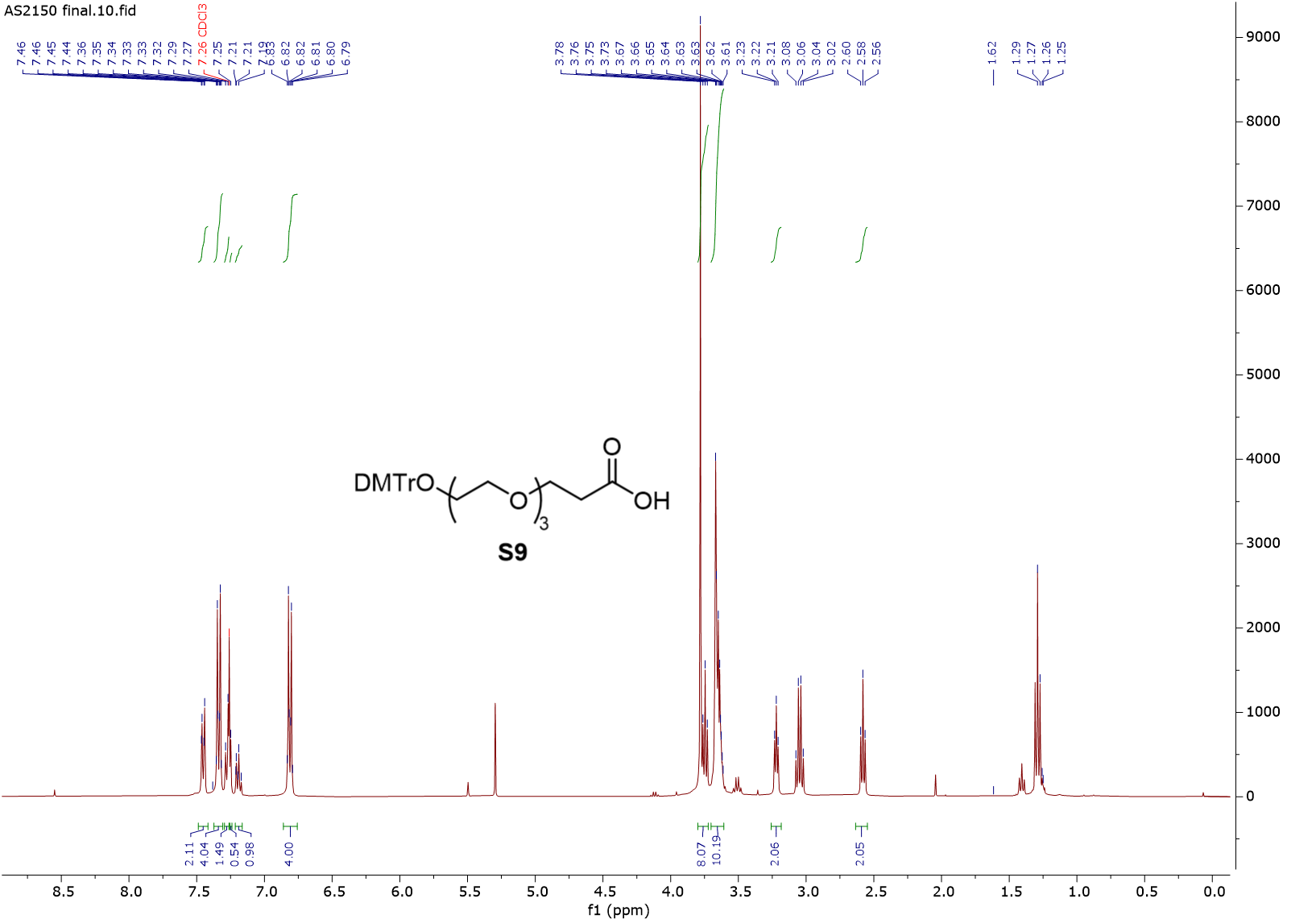
**

^13^CNMR of compound **S9**

**
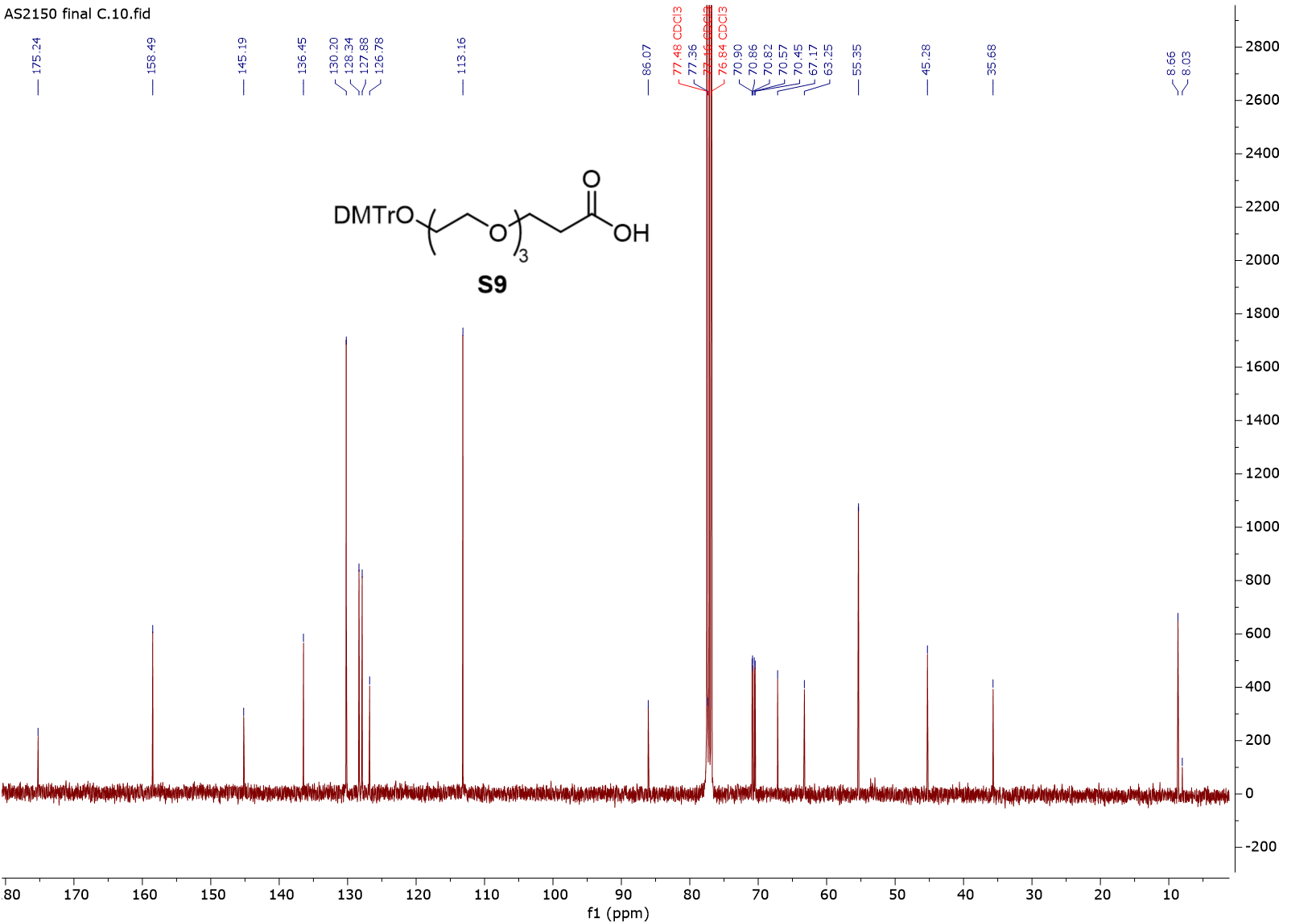
**

^1^HNMR of compound **3g**

**
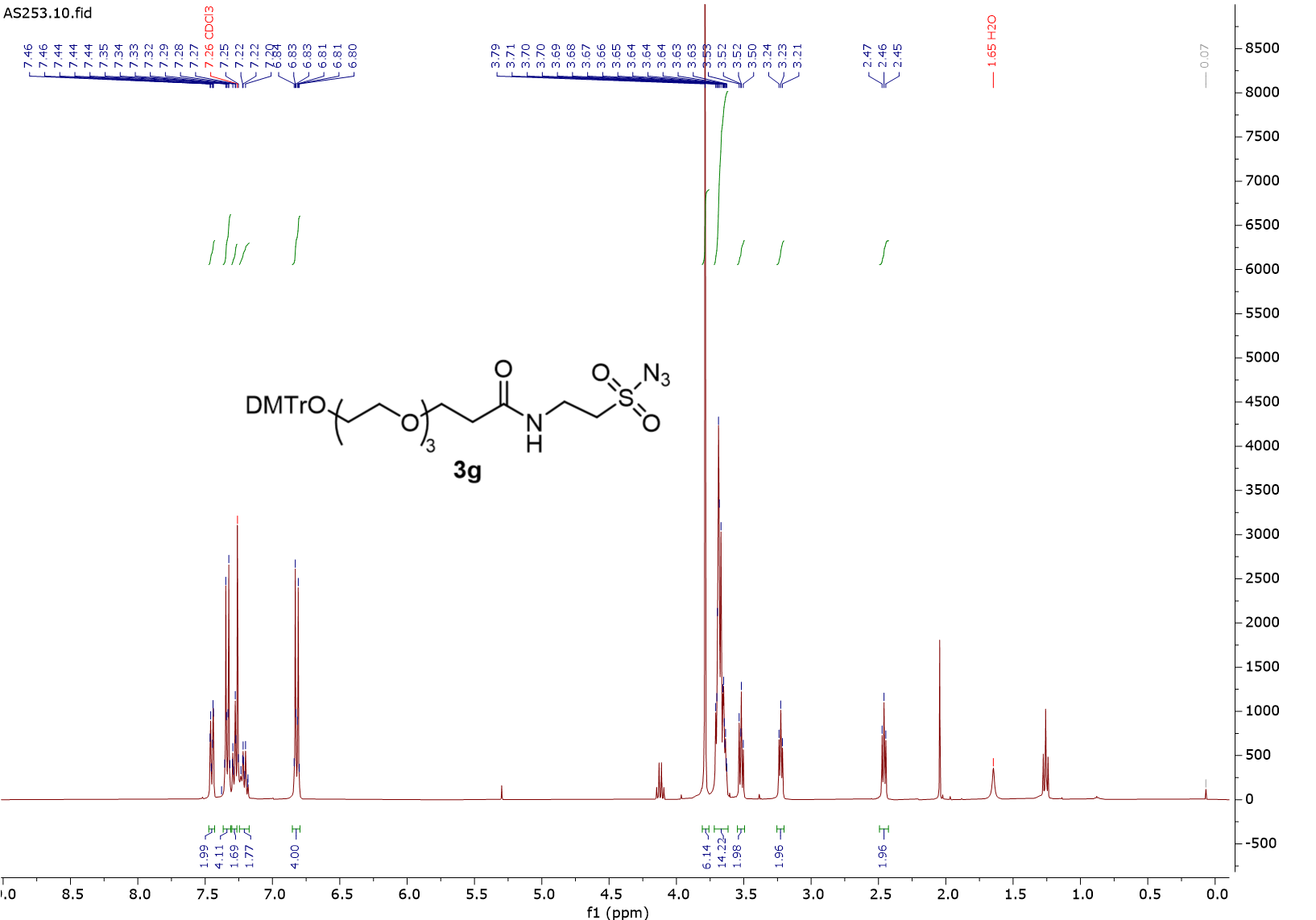
**

^13^CNMR of compound **3g**

**
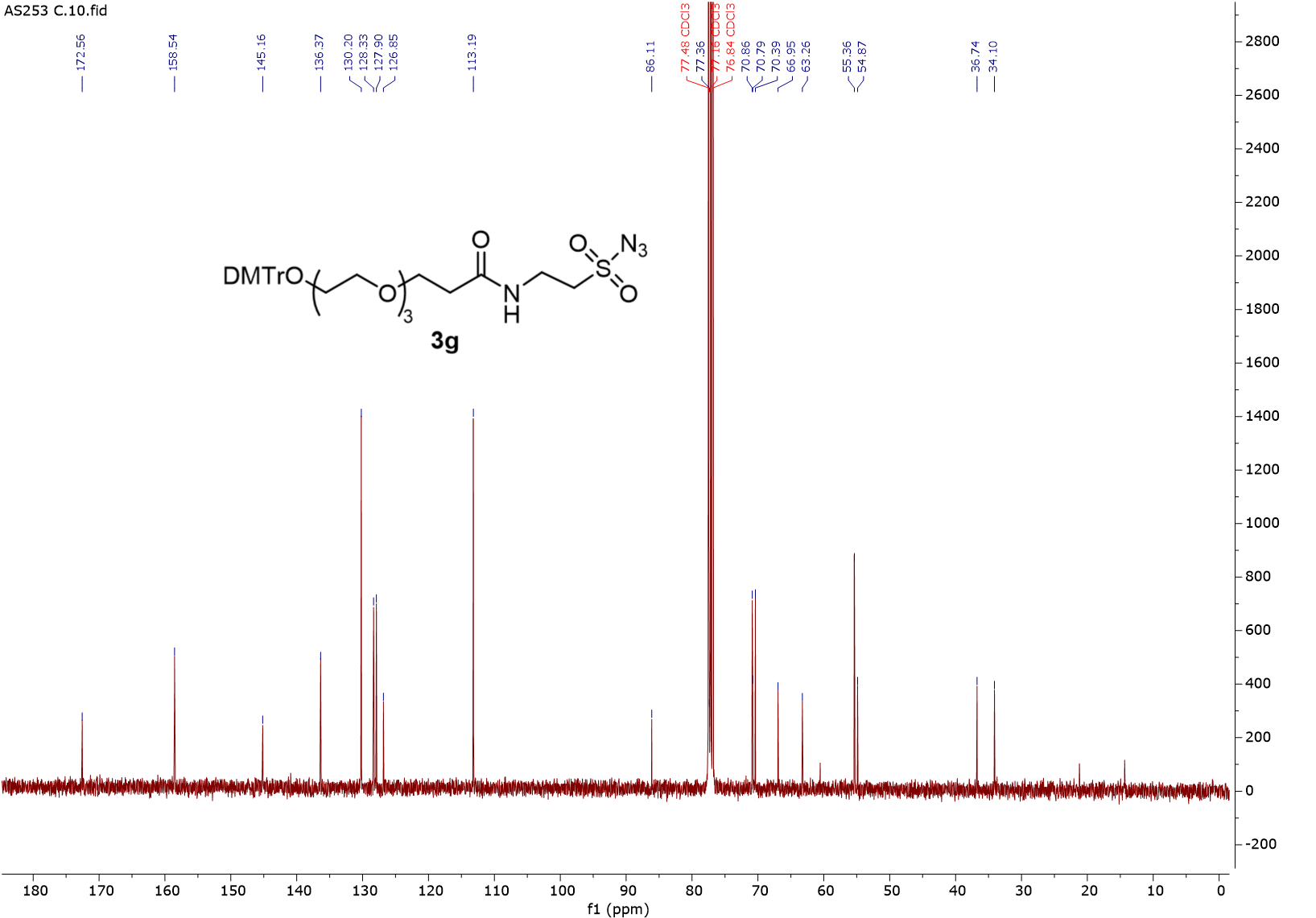
**

^1^HNMR of compound **S10**

**
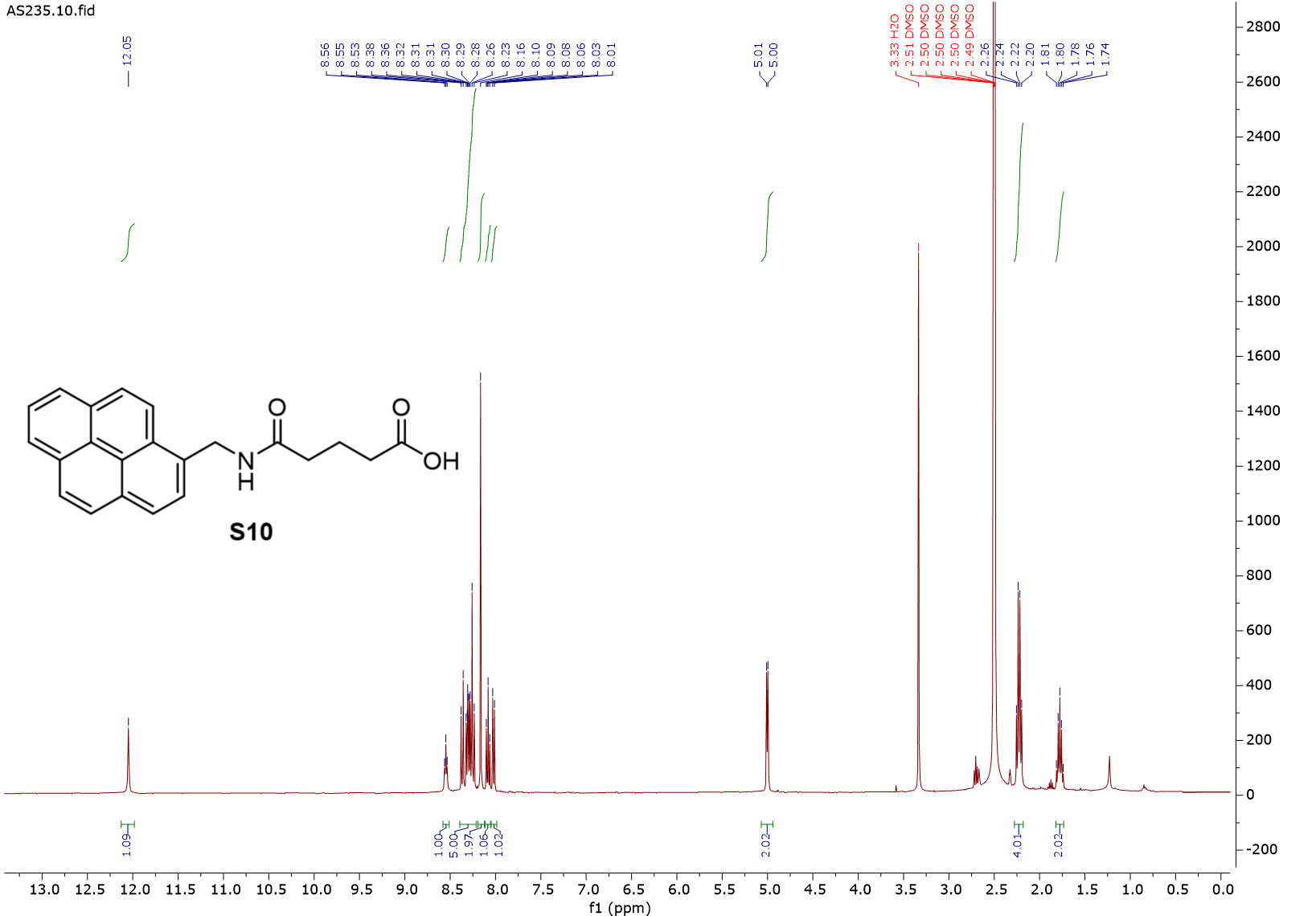
**

^13^CNMR of compound **S10**

**
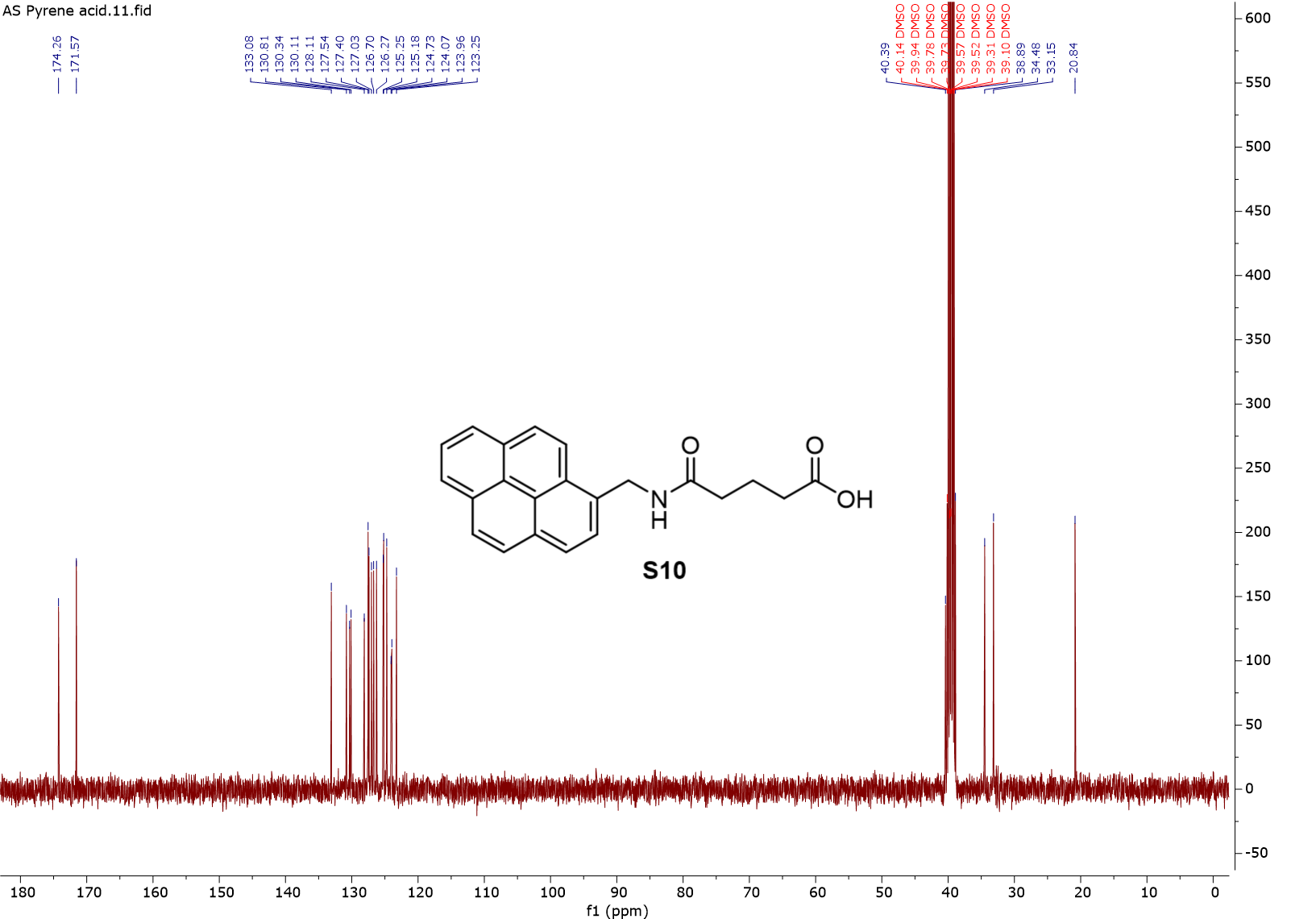
**

^1^HNMR of compound **3h**

**
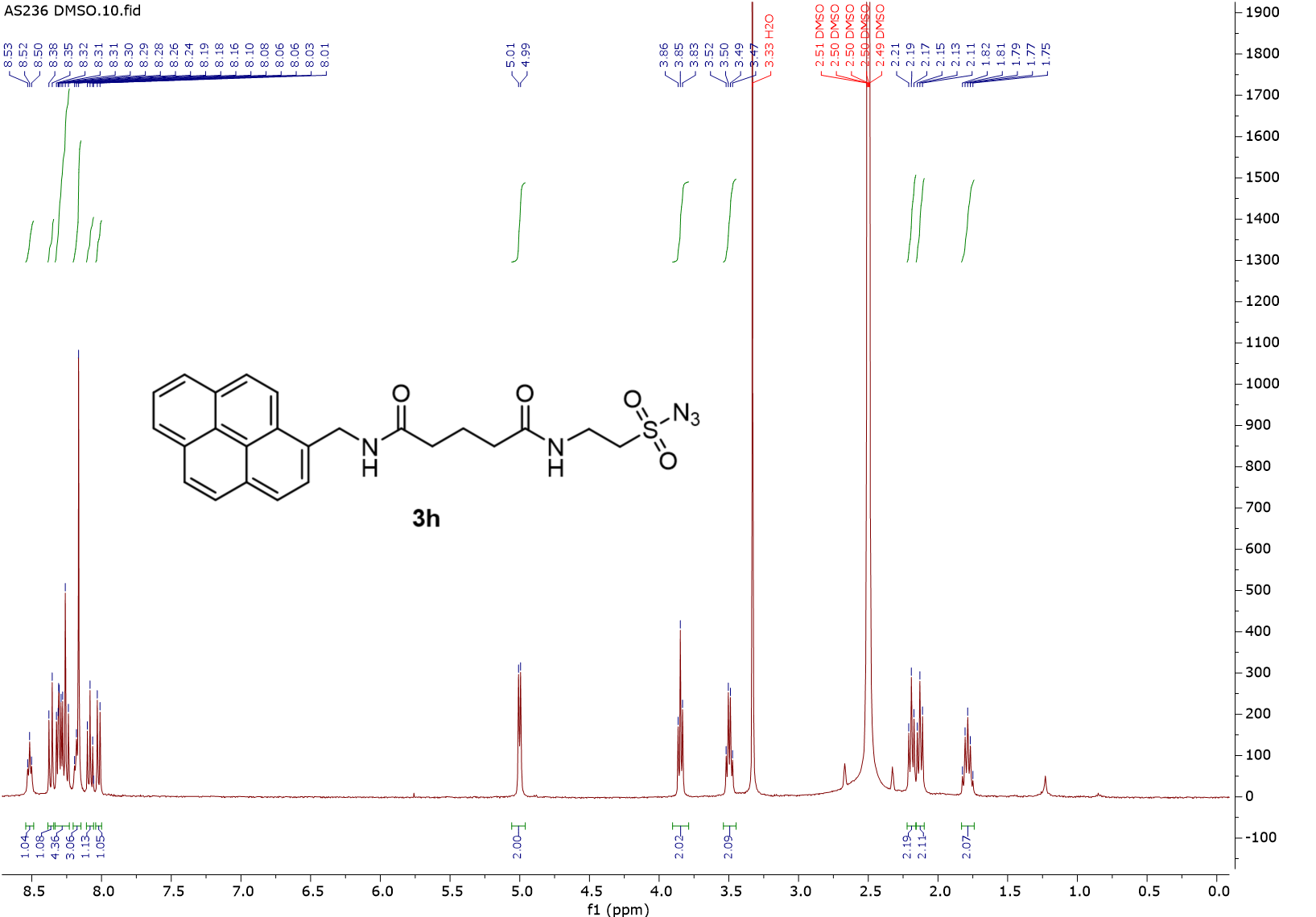
**

^13^CNMR of compound **3h**

**
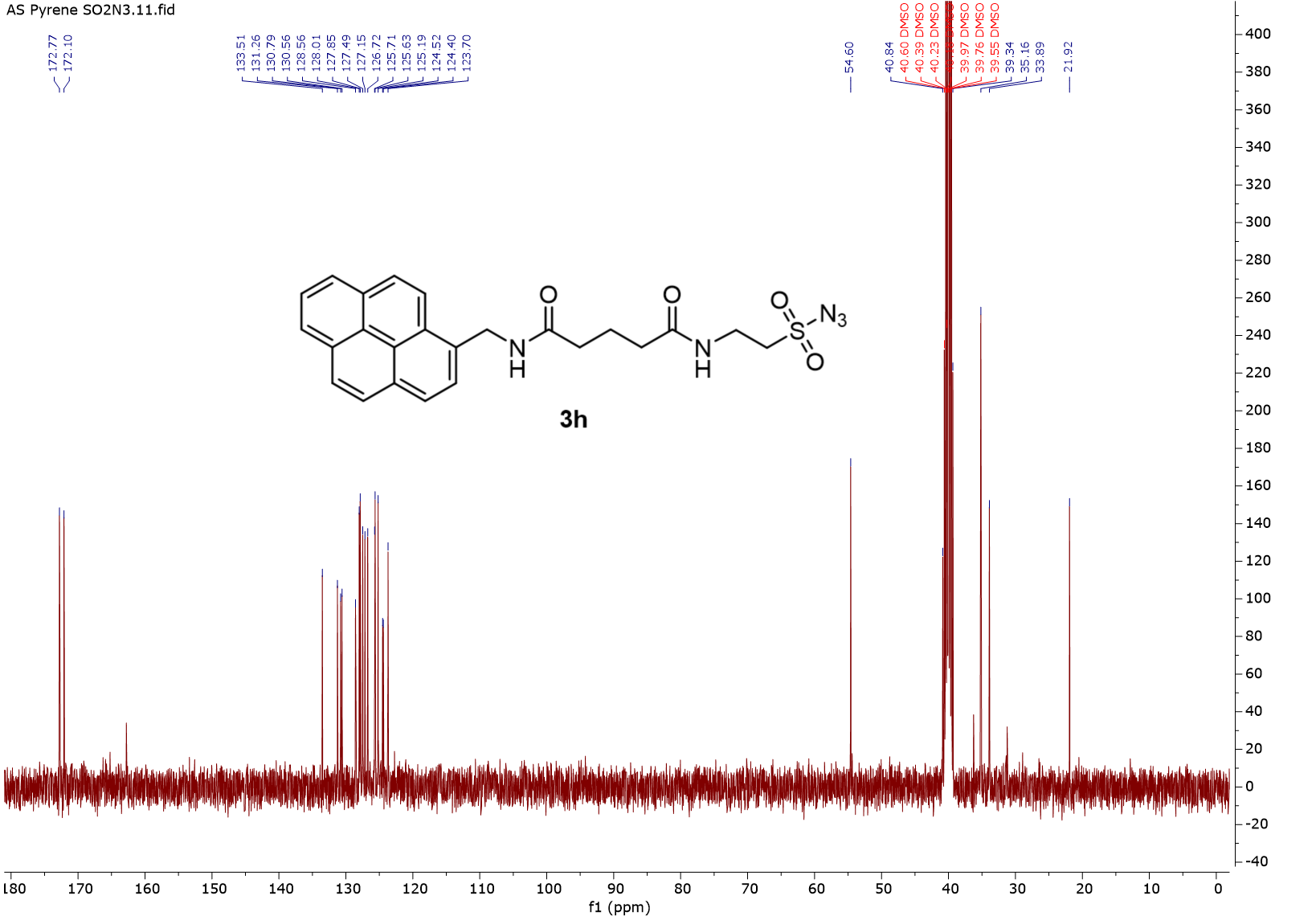
**

^1^HNMR of compound **3i**

**
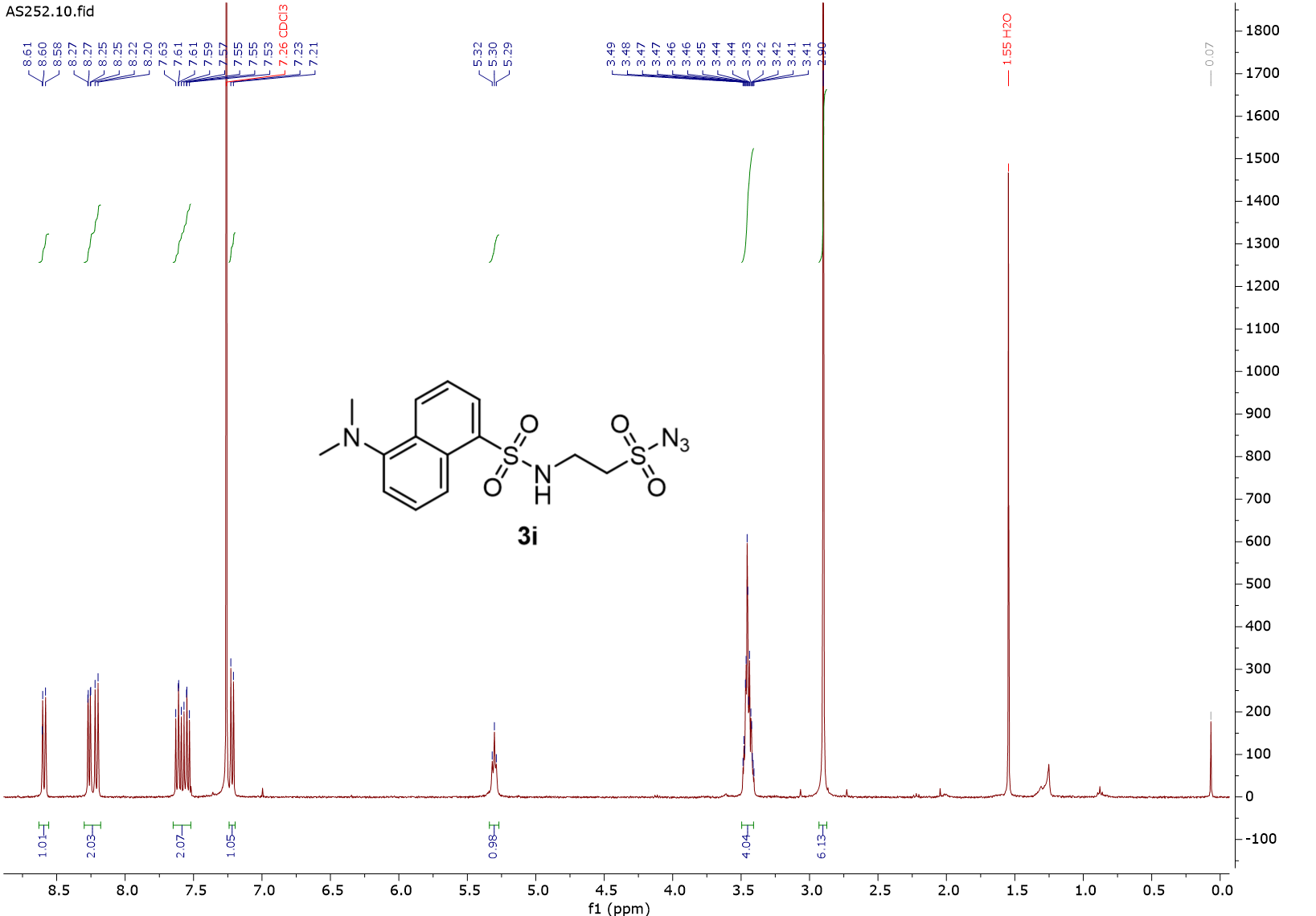
**

^13^CNMR of compound **3i**

**
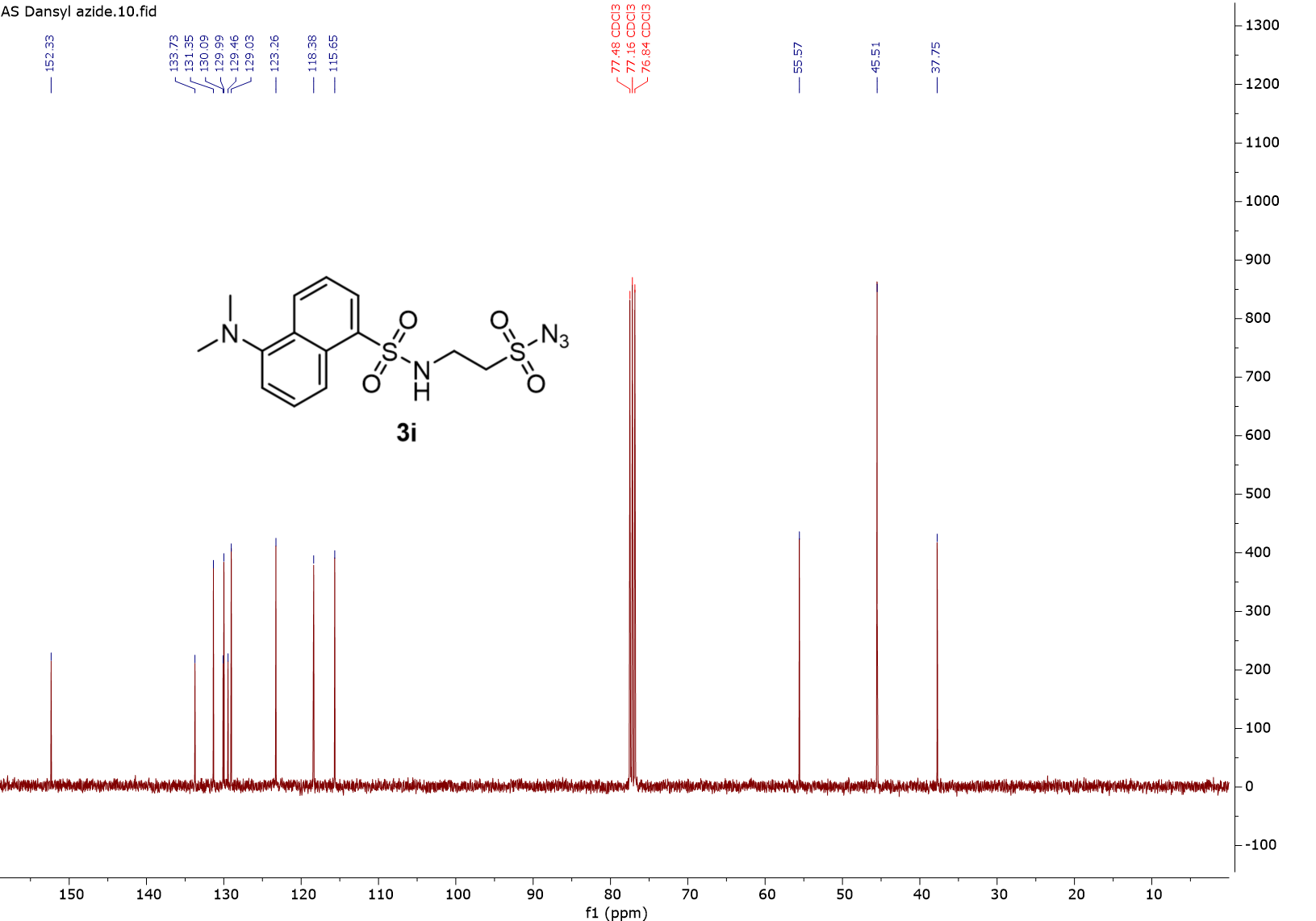
**

^,1^HNMR of compound **S11**

**
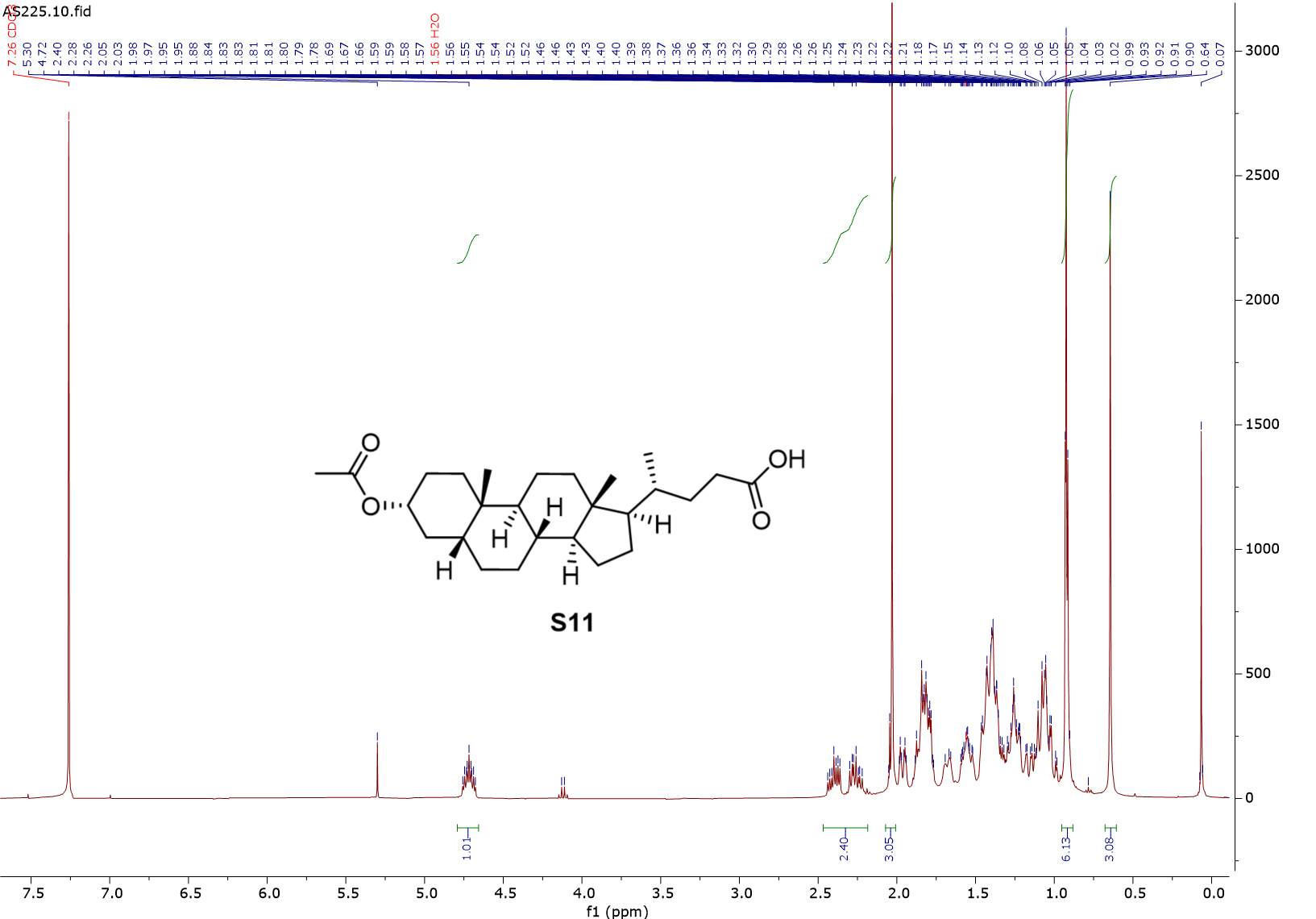
**

^13^CNMR of compound **S11**

**
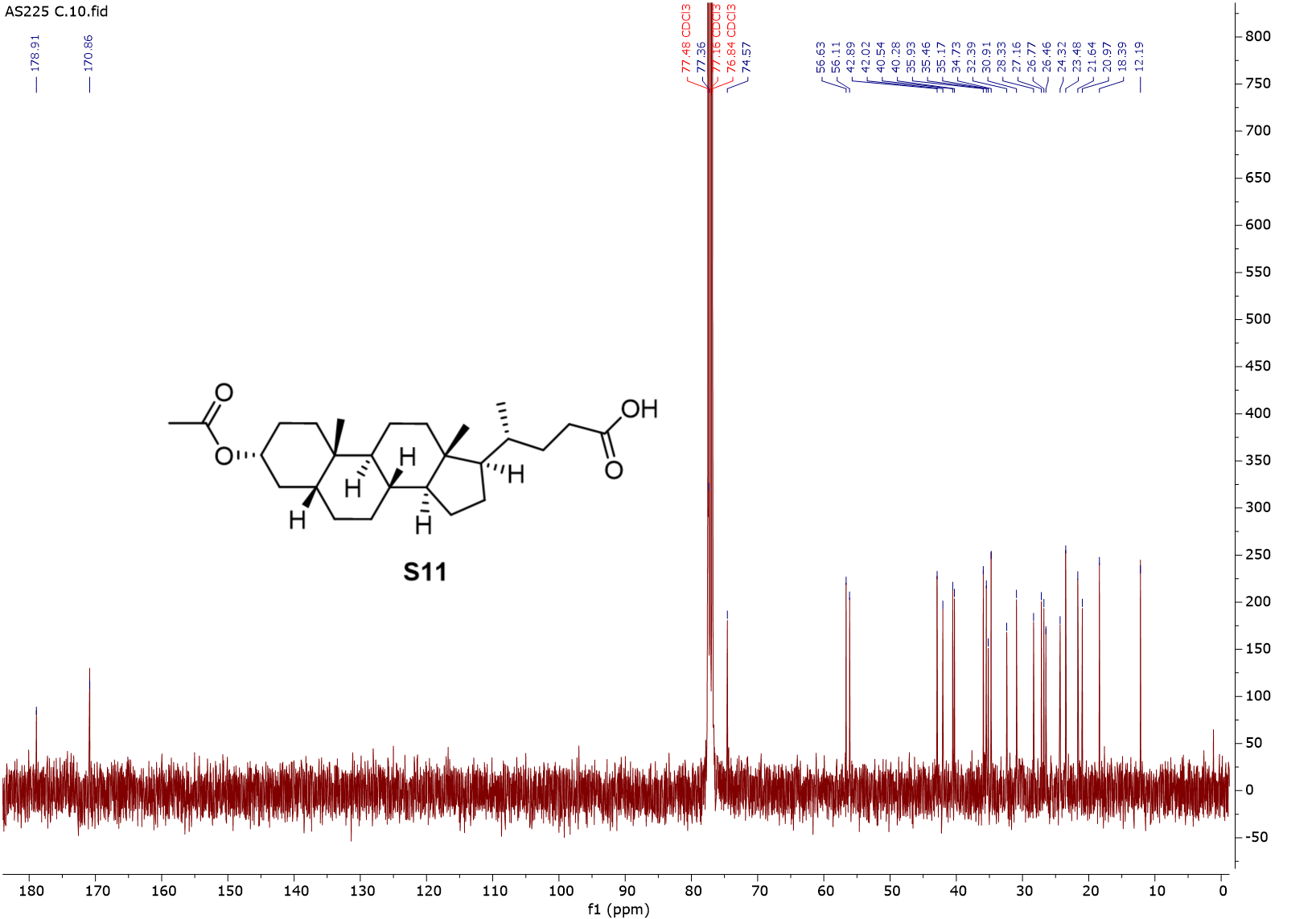
**

^1^HNMR of compound **3j**

**
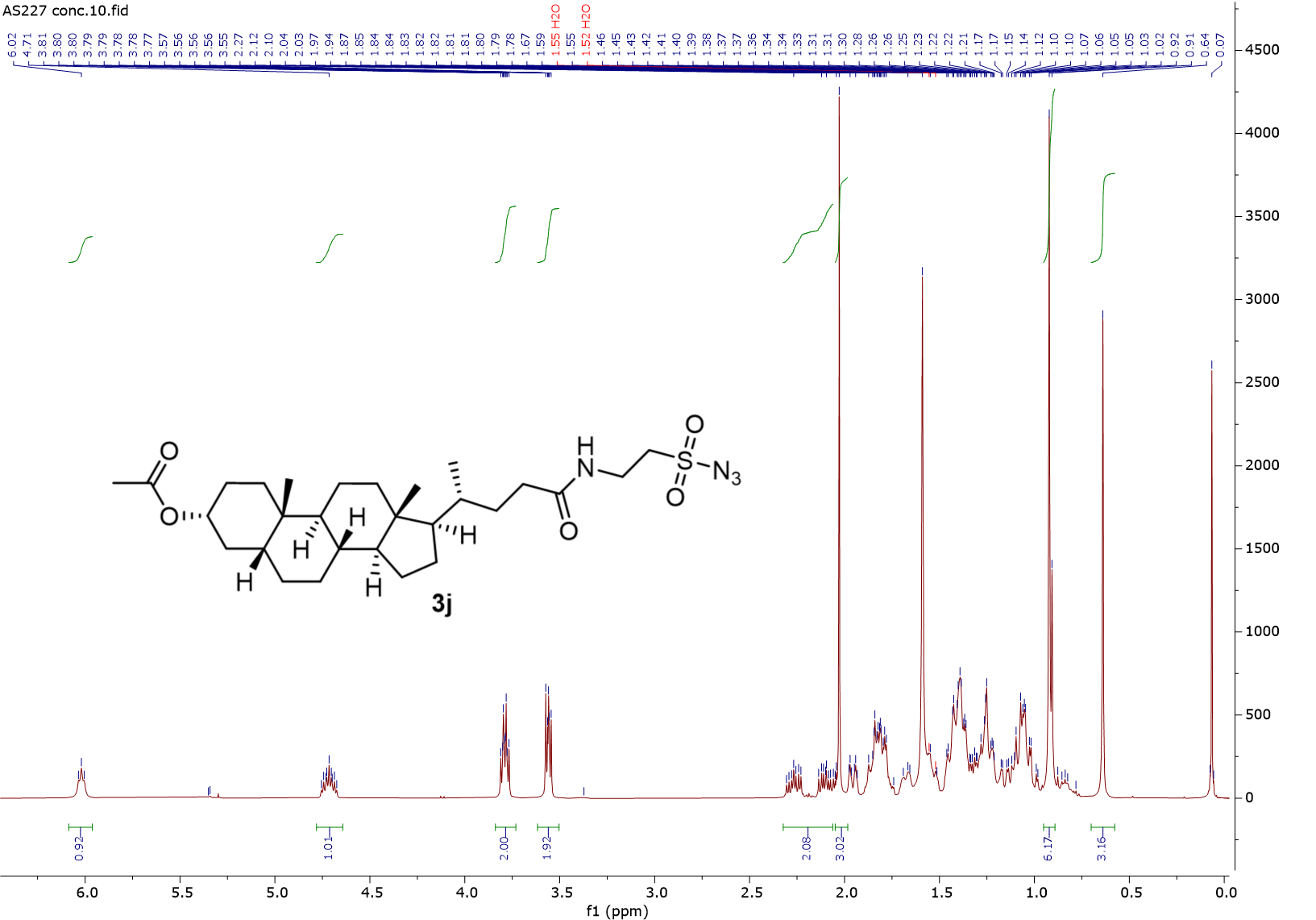
**

^13^CNMR of compound **3j**

**
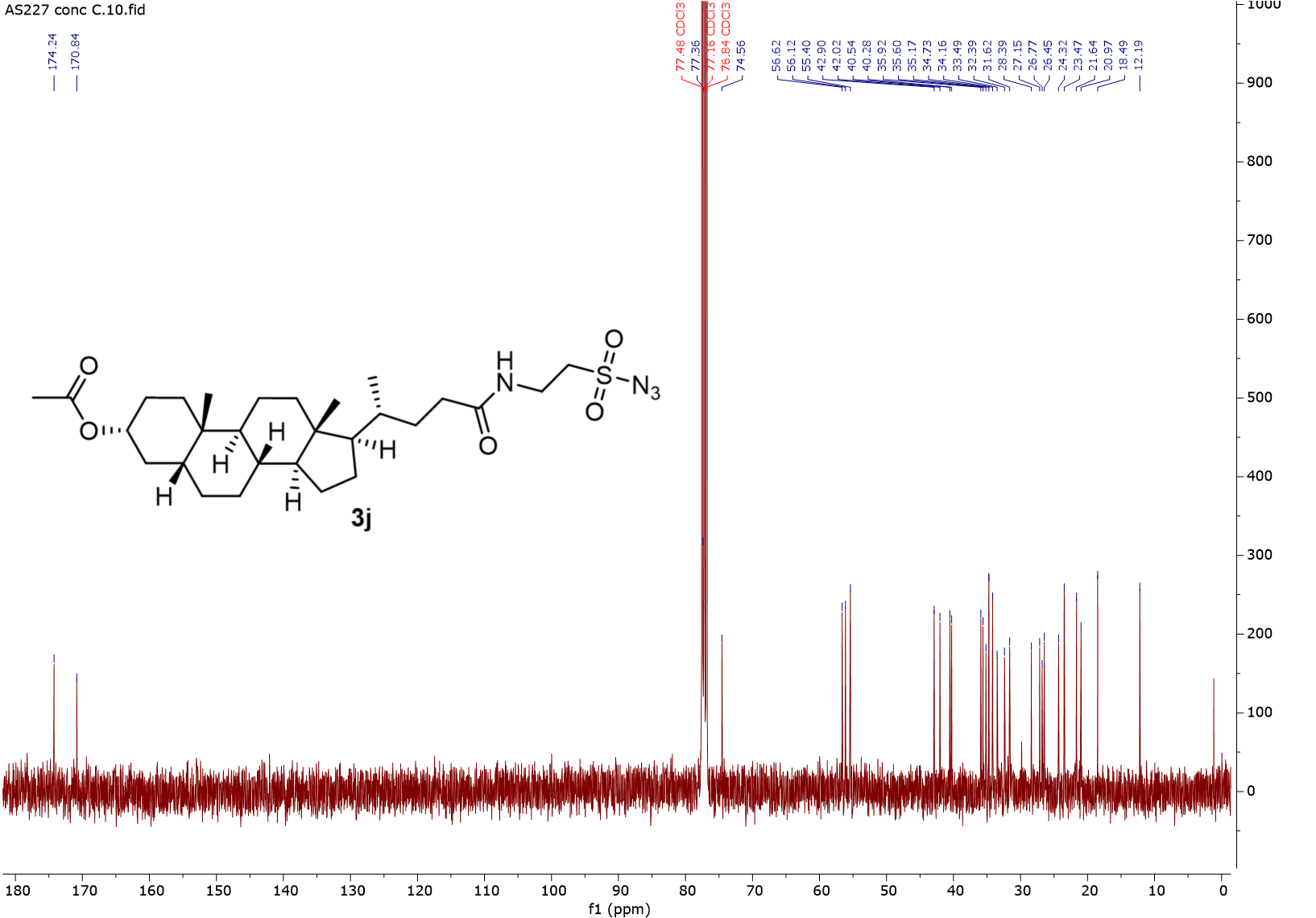
**

^1^HNMR of compound **3k**

**
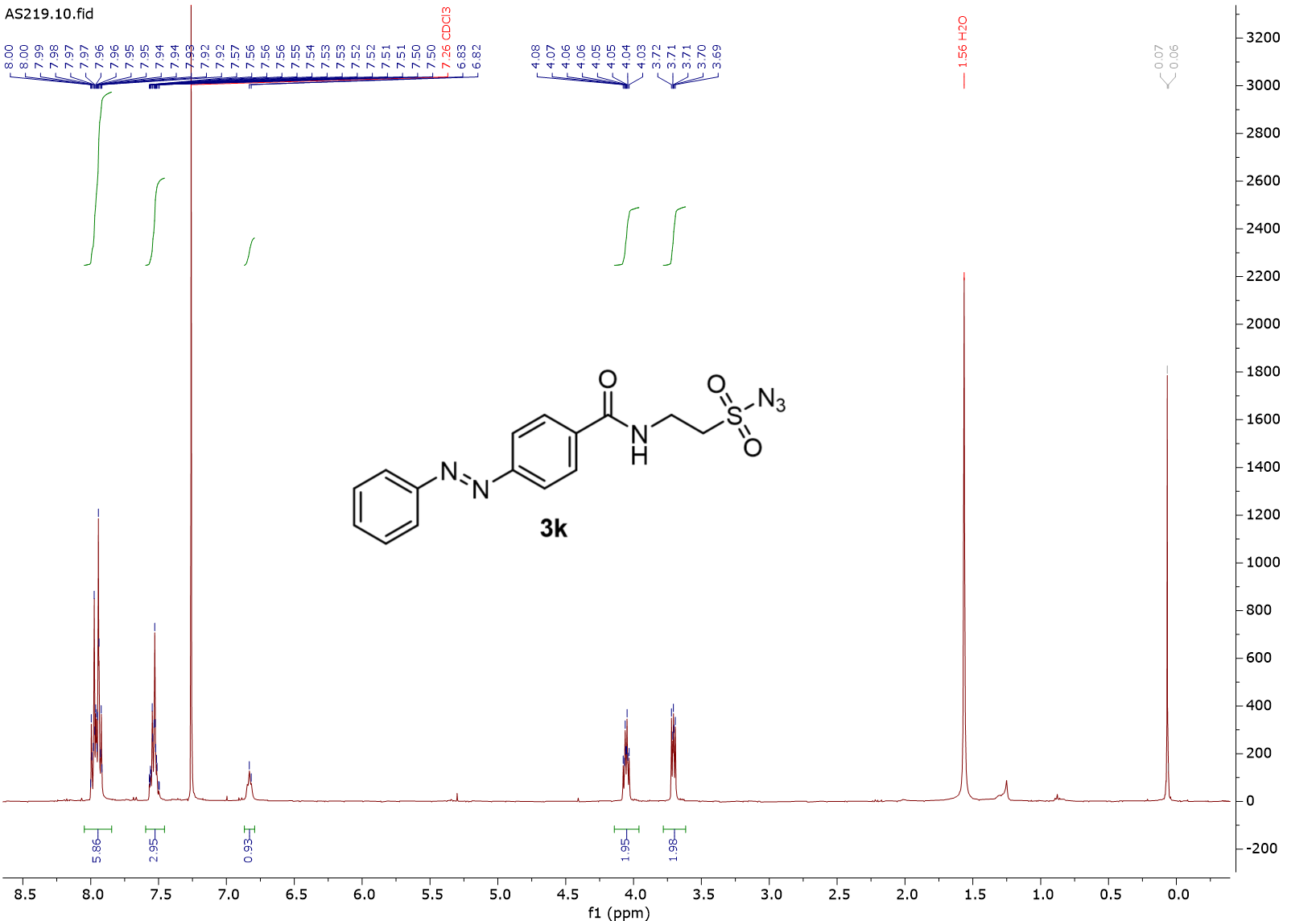
**

^13^CNMR of compound **3k**

**
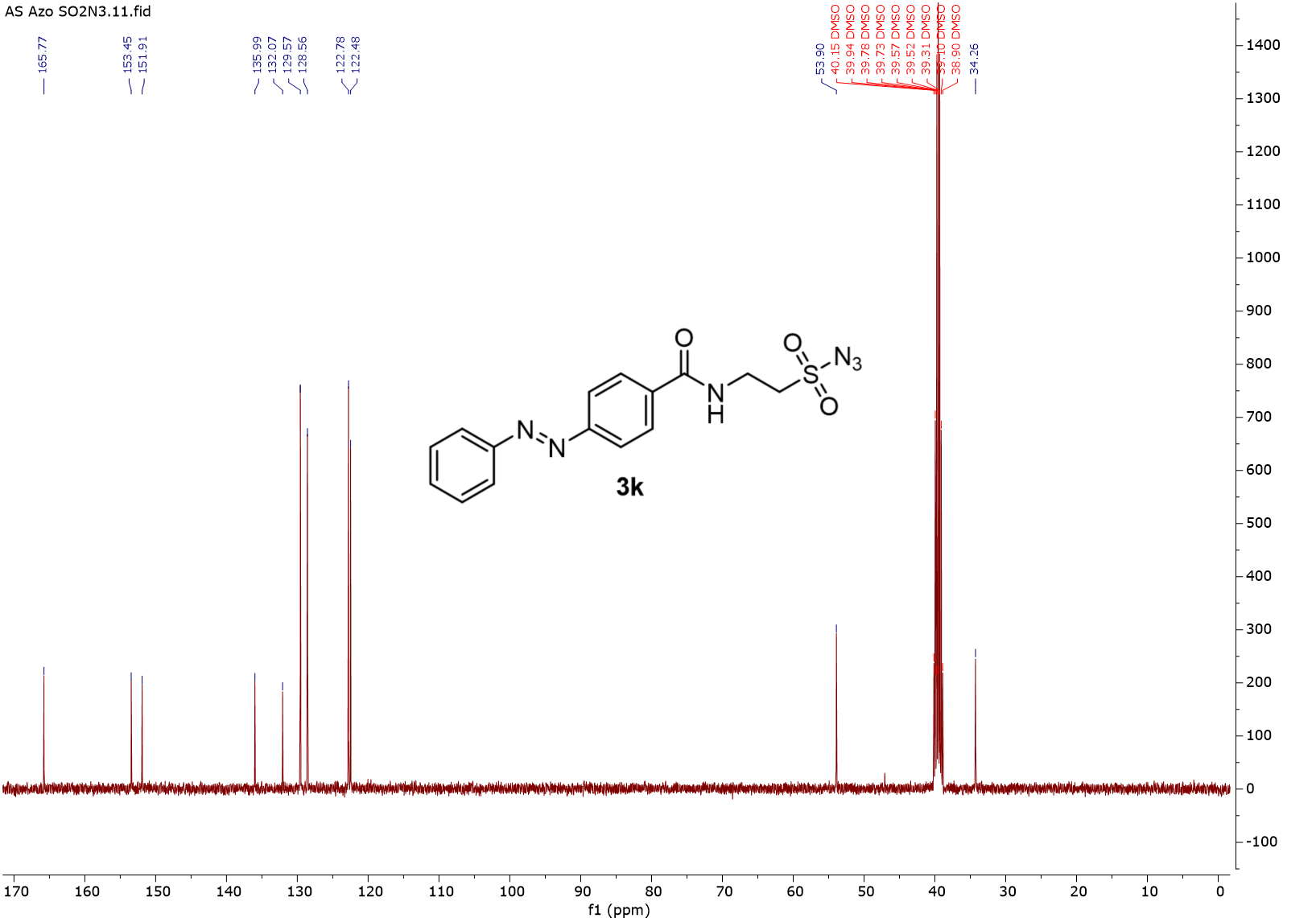
**

**Optimization of solid phase Staudinger reaction for DNA-Modification**

To optimize the reaction time, the synthesis of a poly-T 10-sequence in which the final phosphite in the sequence was subjected to the sulfonyl azide 3e at different coupling times was performed**.**

**
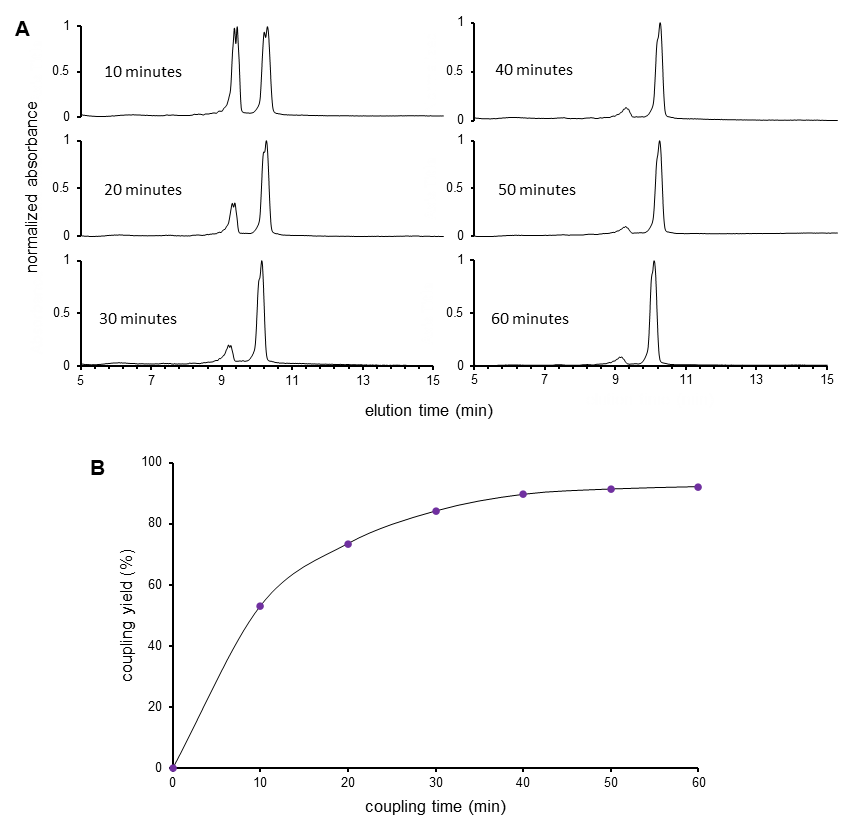
**

**Figure S1.** Time study of the Staudinger reaction within 3e and CPG-support-bound phosphites. (**A**) Chromatograms of the poly-T 10-mer final phosphite reacting with 3e for 10 - 60 minutes. (**B**) Coupling yield as a function of reaction time for the synthesis of 5’-T(4e)TTTTTTTTT-3’.

**Mass spectrum characterization of the synthesized strands**


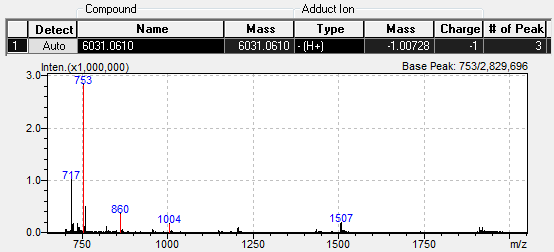


MS and mass deconvolution of strand **5a**


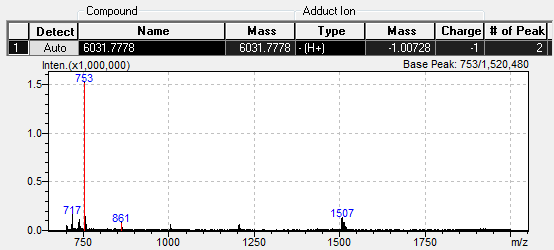


MS and mass deconvolution of strand **5b**


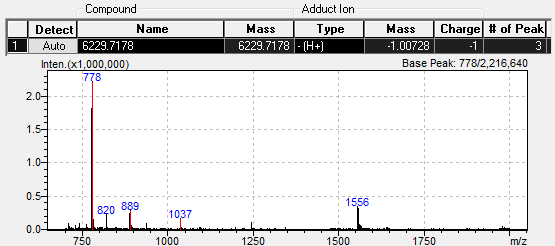


MS and mass deconvolution of strand **5c**


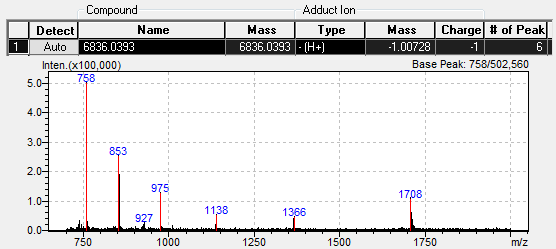


MS and mass deconvolution of strand **5c3**


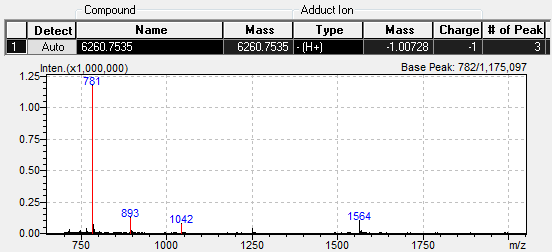


MS and mass deconvolution of strand **5d**


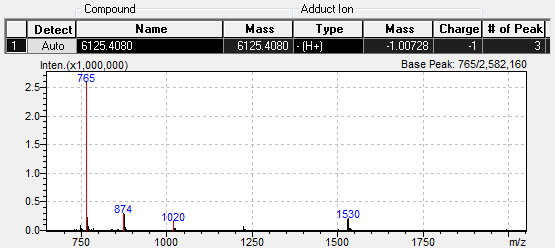


MS and mass deconvolution of strand **5e**


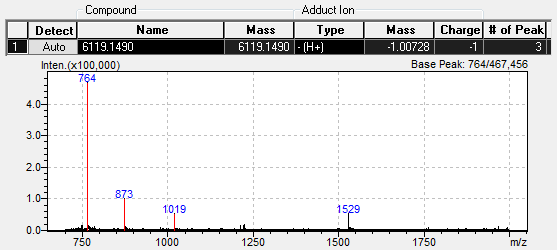


MS and mass deconvolution of strand **5f**


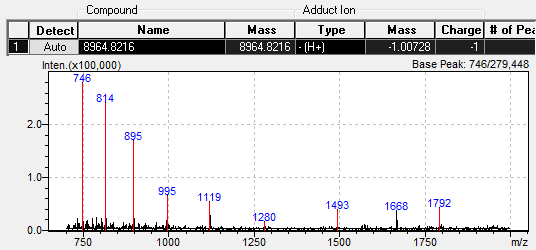


MS and mass deconvolution of strand **5g**


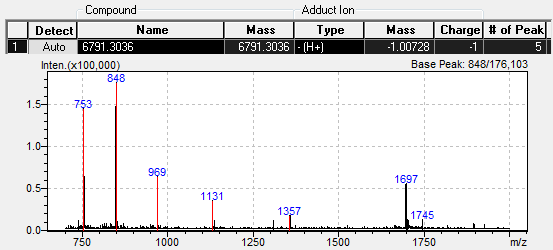


MS and mass deconvolution of strand **5h2**


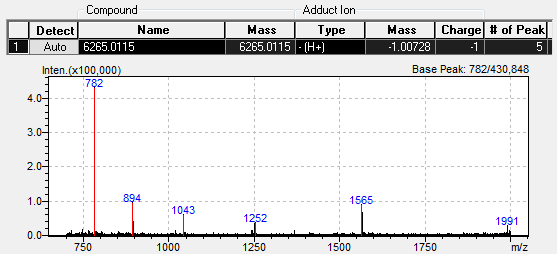


MS and mass deconvolution of strand **5i**


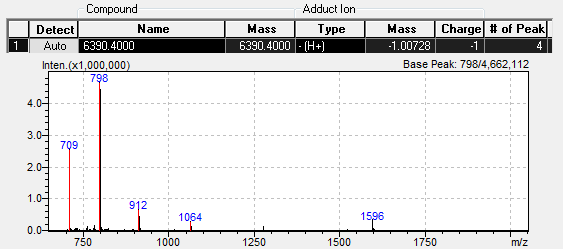


MS and mass deconvolution of strand **5j**


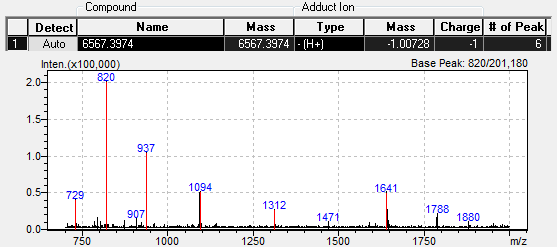


MS and mass deconvolution of strand **5bde**


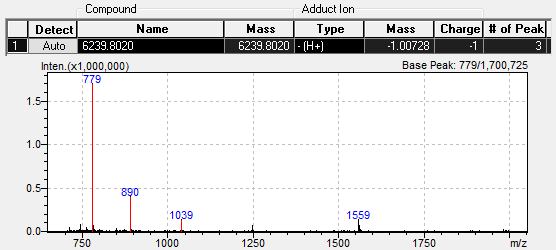


MS and mass deconvolution of strand **5k**


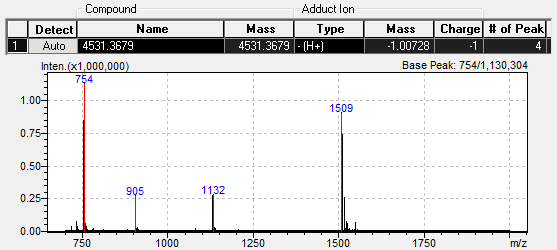


MS and mass deconvolution of strand **7d1’**


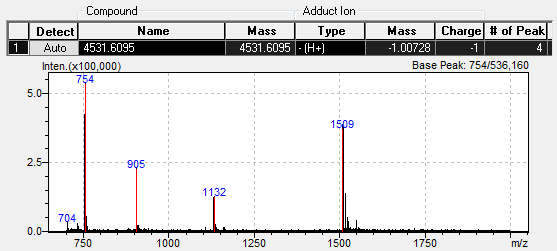


MS and mass deconvolution of strand **7d2’**


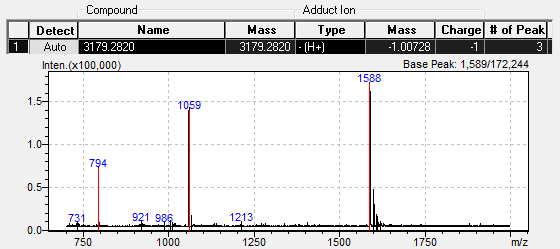


MS and mass deconvolution of strand **7e**


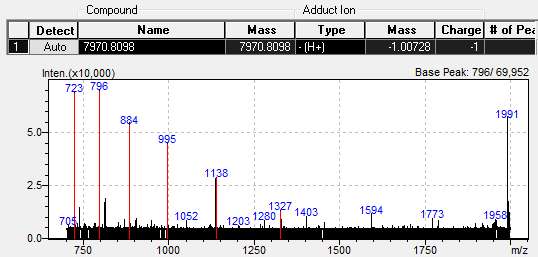


MS and mass deconvolution of strand **8k4**

**DNA melting curves**


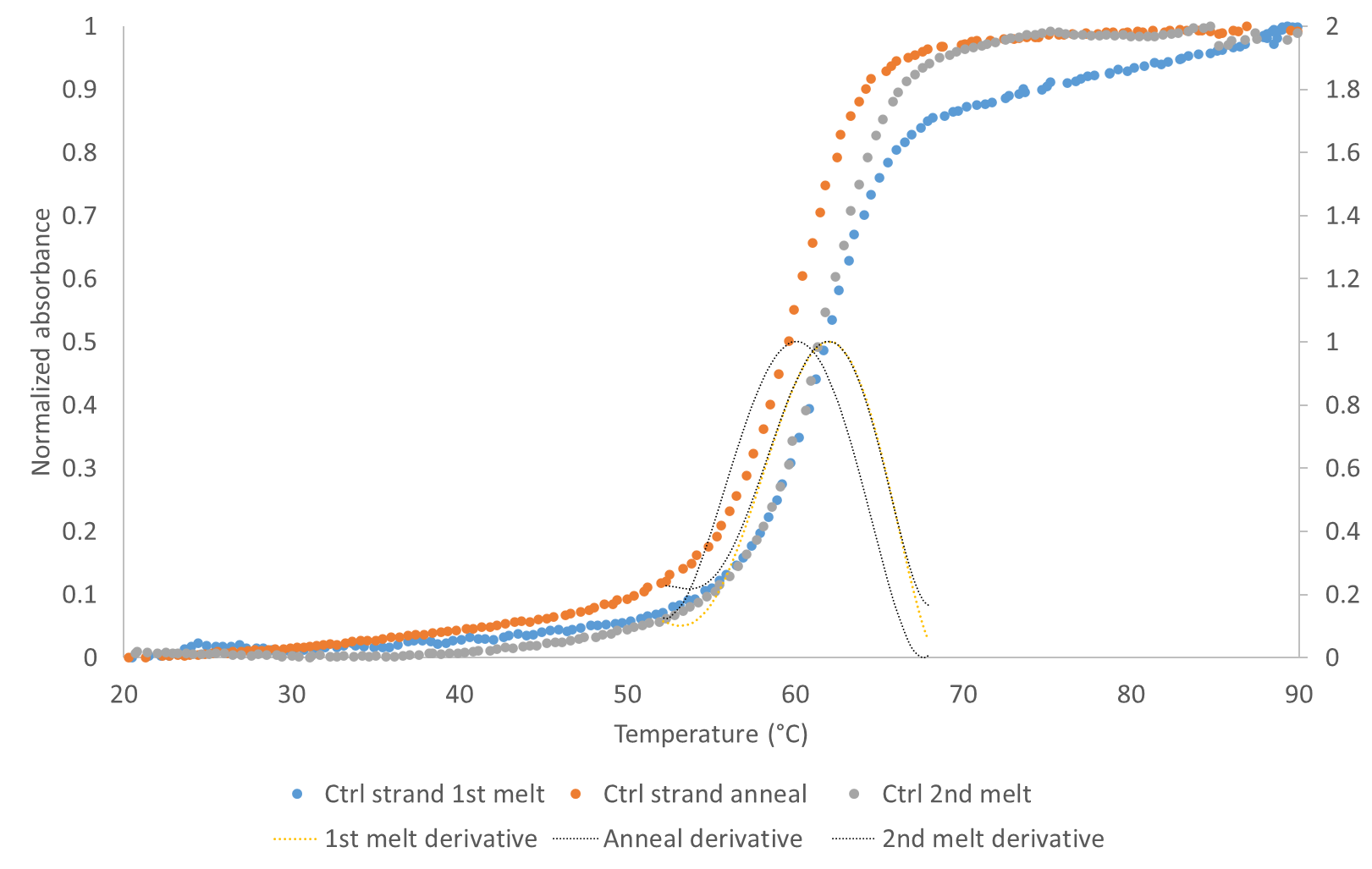


**Figure S2**. DNA melting curve of the control non-modified strands (5’-CCTAATCAACTCCACTCCCA-3’) and its complementary strand (5'-TGGGAGTGGAGTTGATTAGG-3').


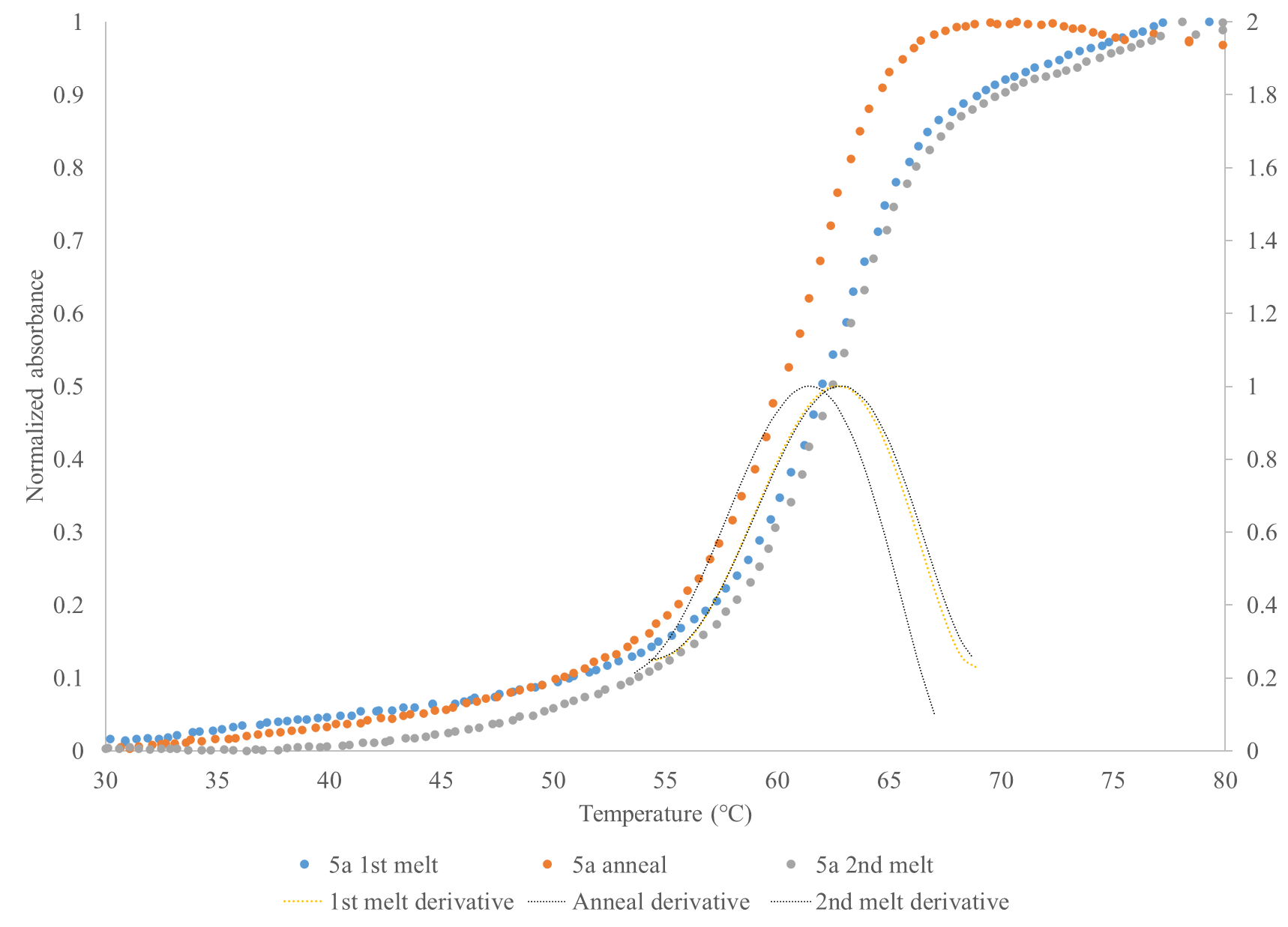


**Figure S3.** DNA melting curve of 5a/b and the complementary strand.


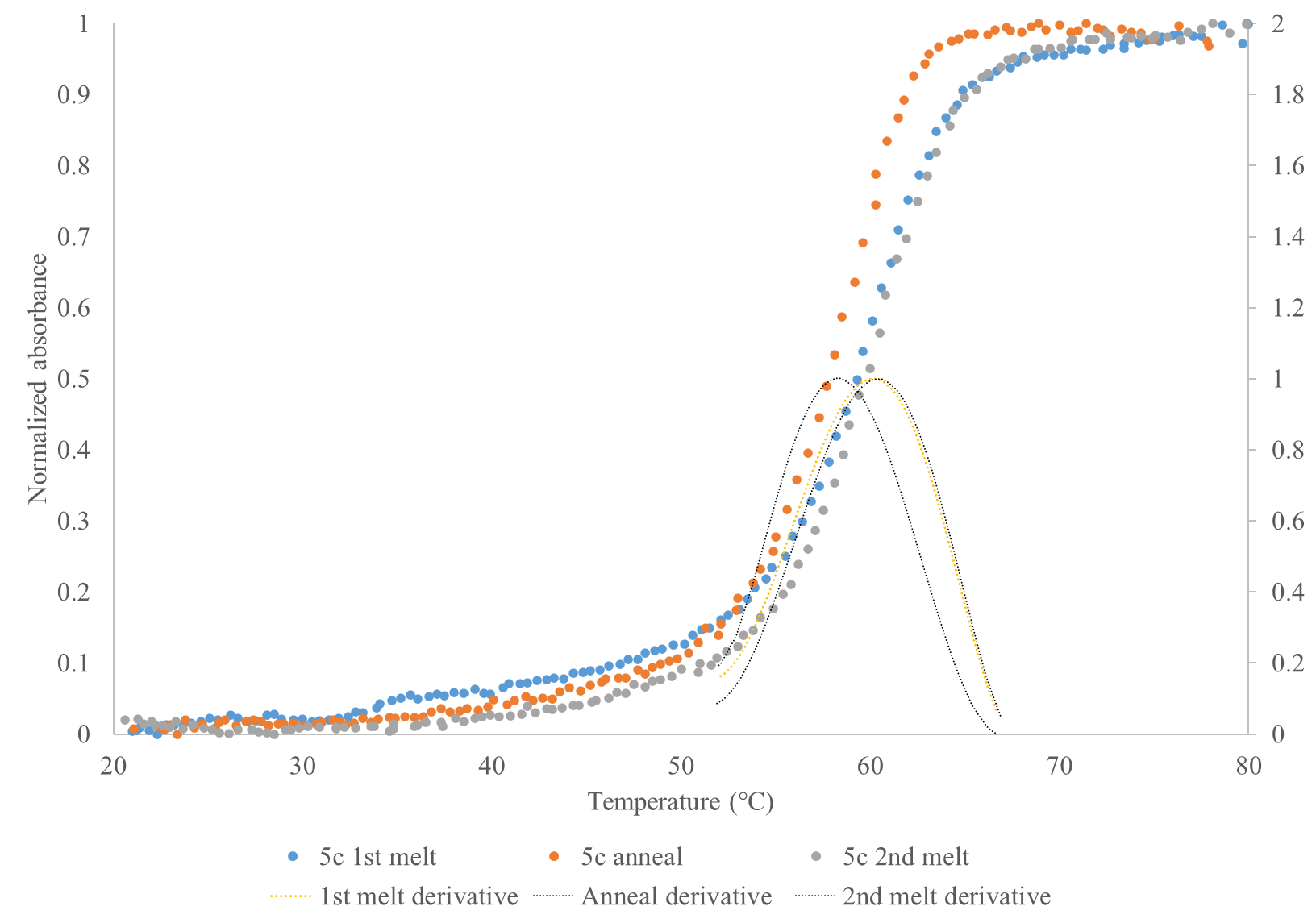


**Figure S4.** DNA melting curve of **5c** and the complementary strand.


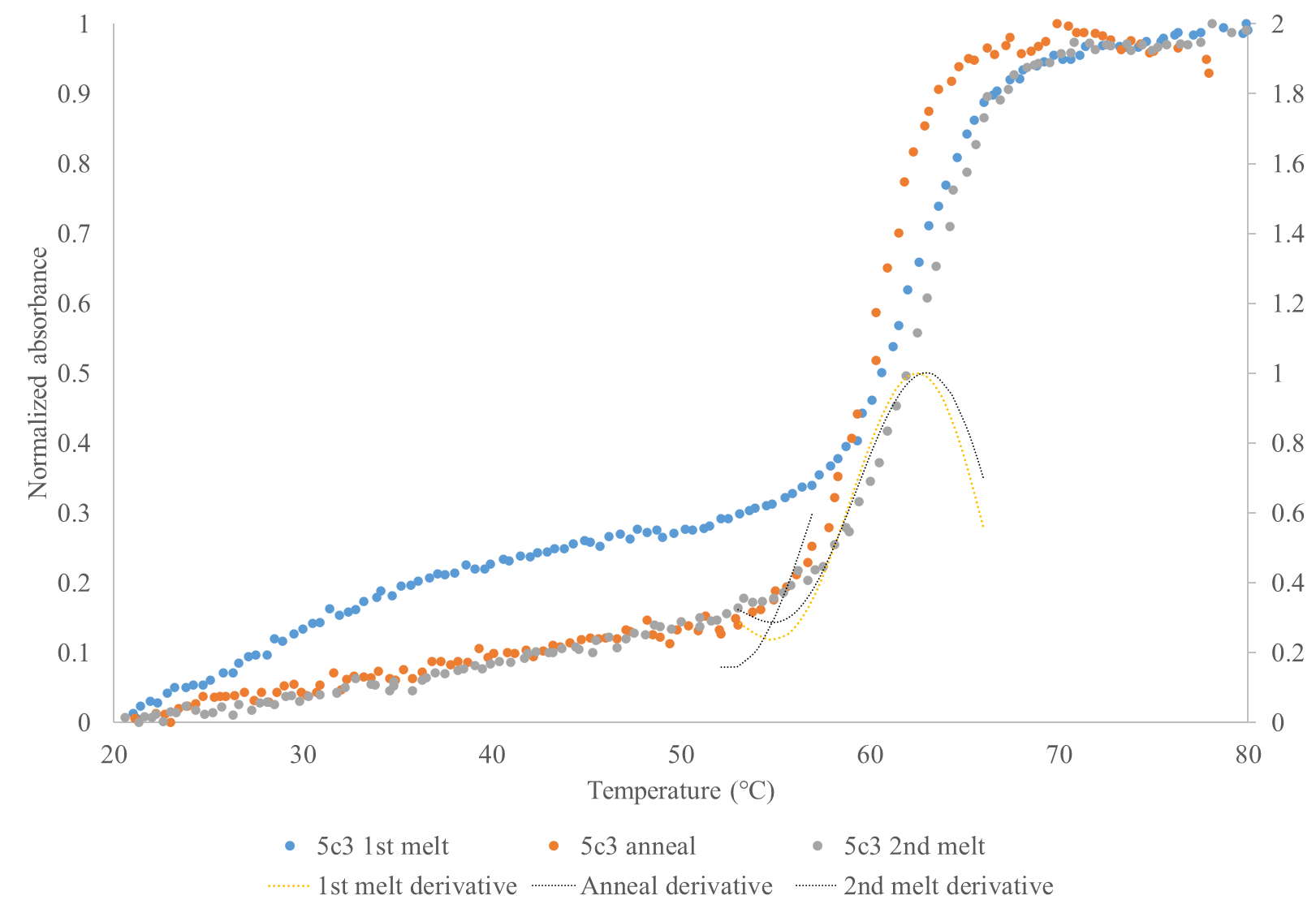


**Figure S5.** DNA melting curve of **5c3** the complementary strand.


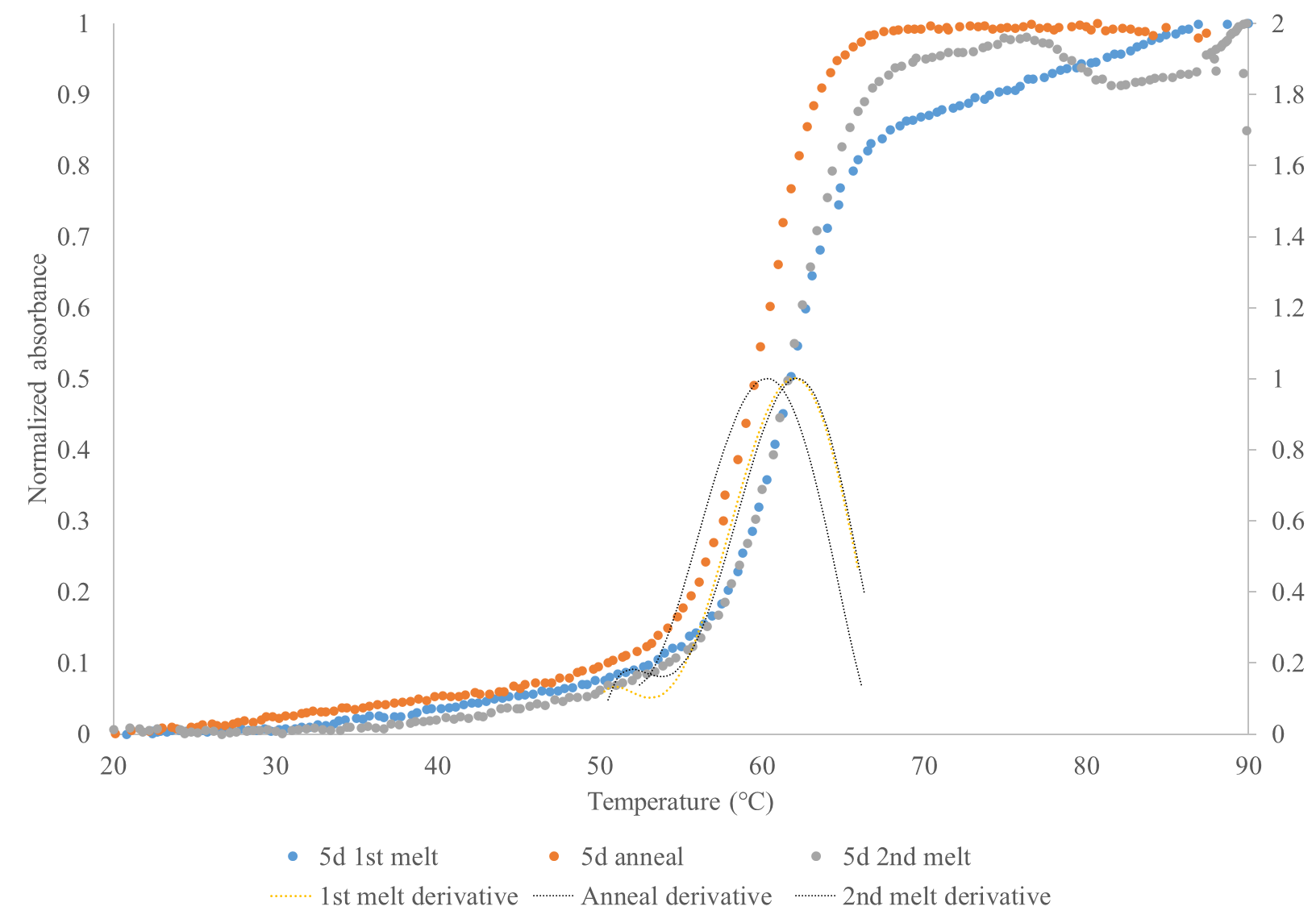


**Figure S6.** DNA melting curve of **5d** and the complementary strand.


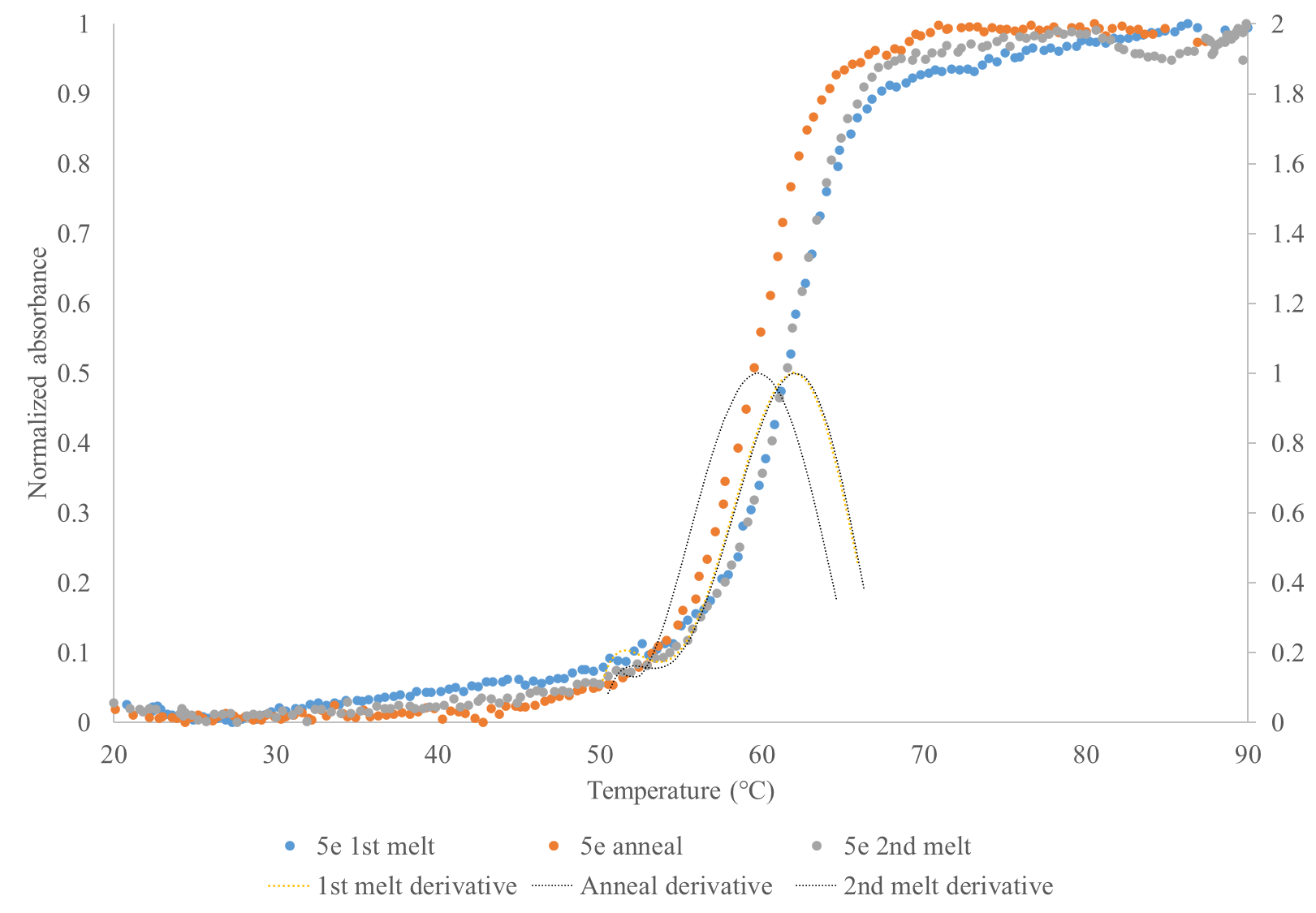


**Figure S7.** DNA melting curve of **5e** and the complementary strand.


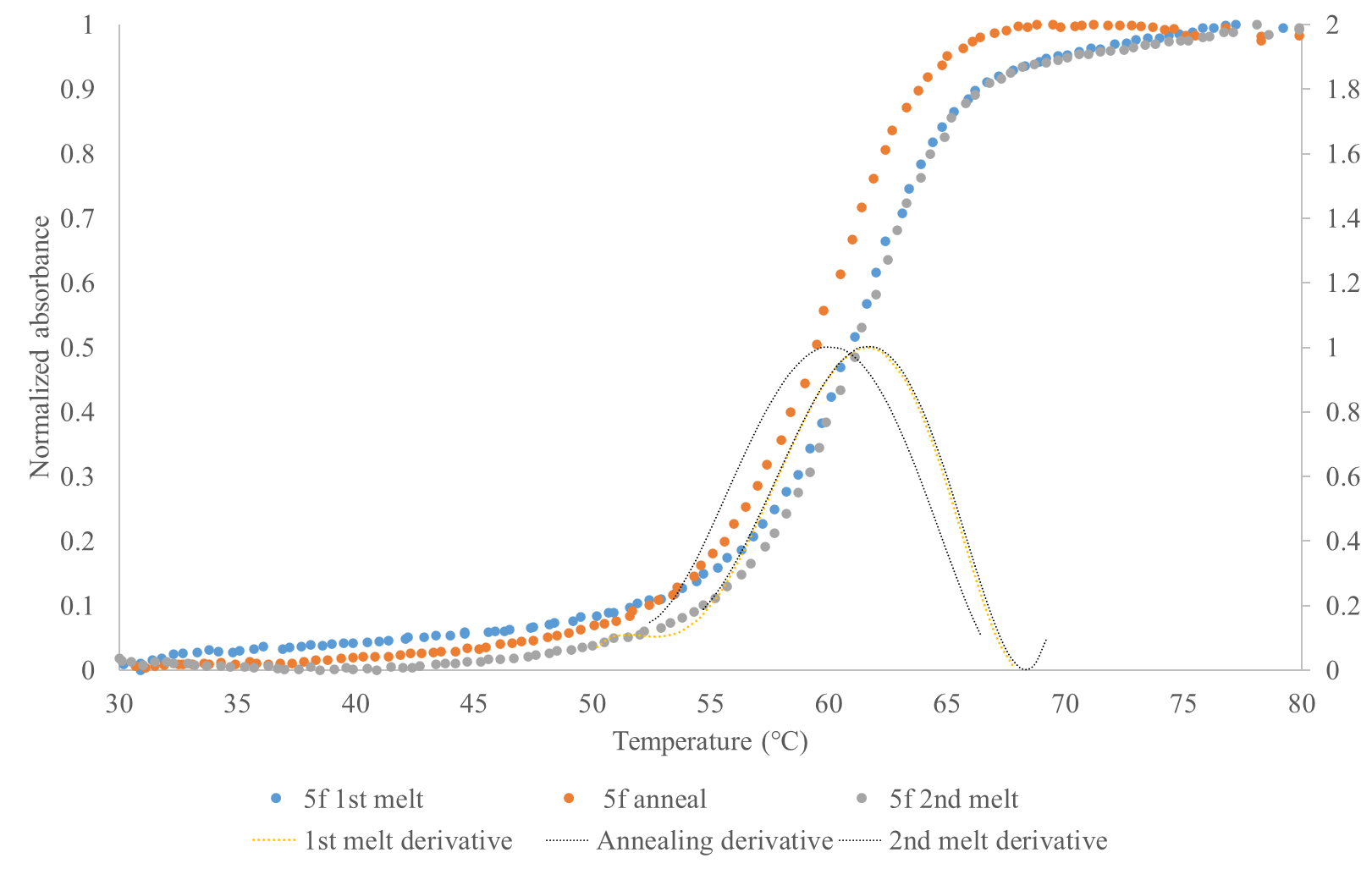


**Figure S8.** DNA melting curve of **5f** and the complementary strand**.**


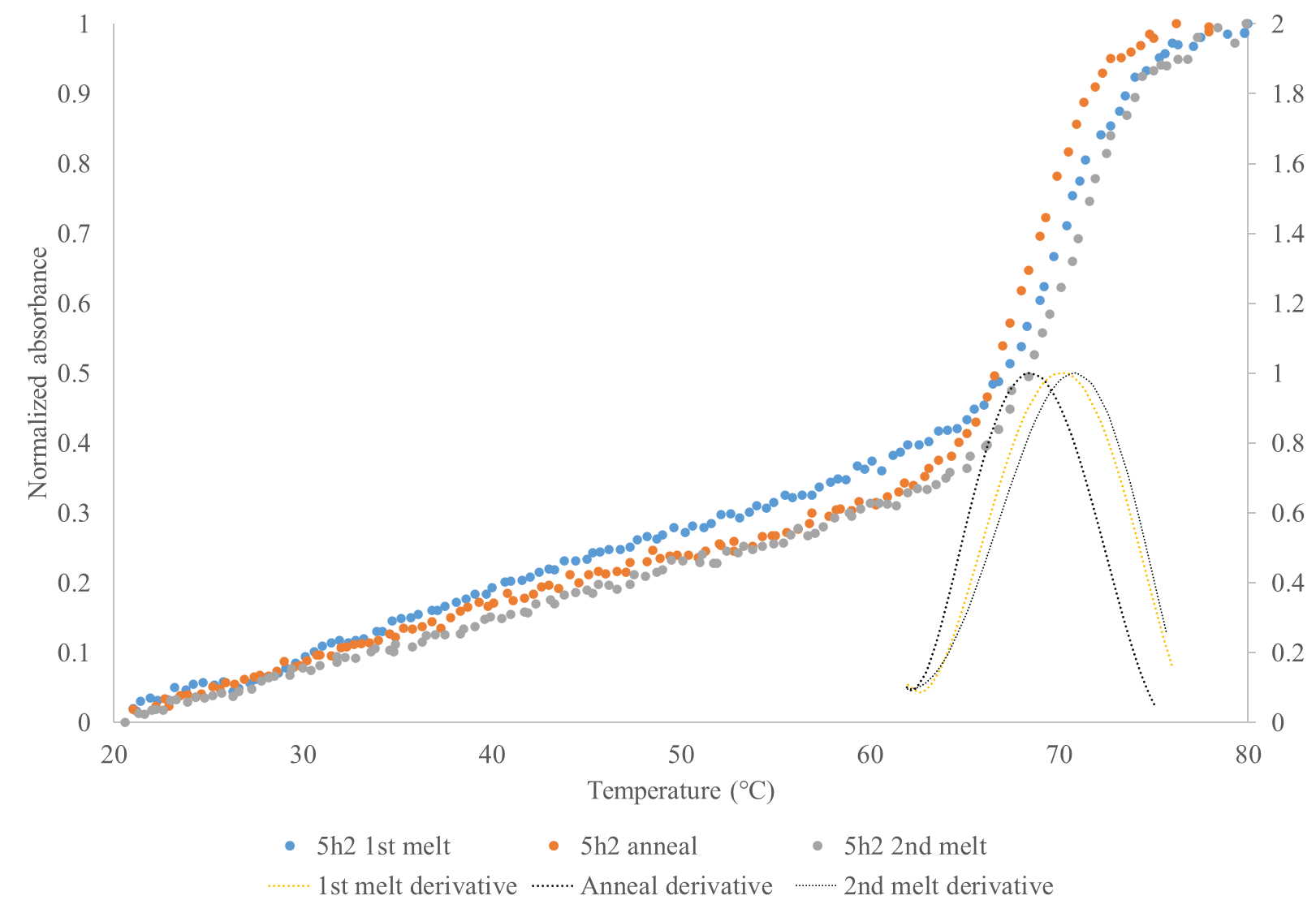


**Figure S9.** DNA melting curve of **5h2** and the complementary strand.


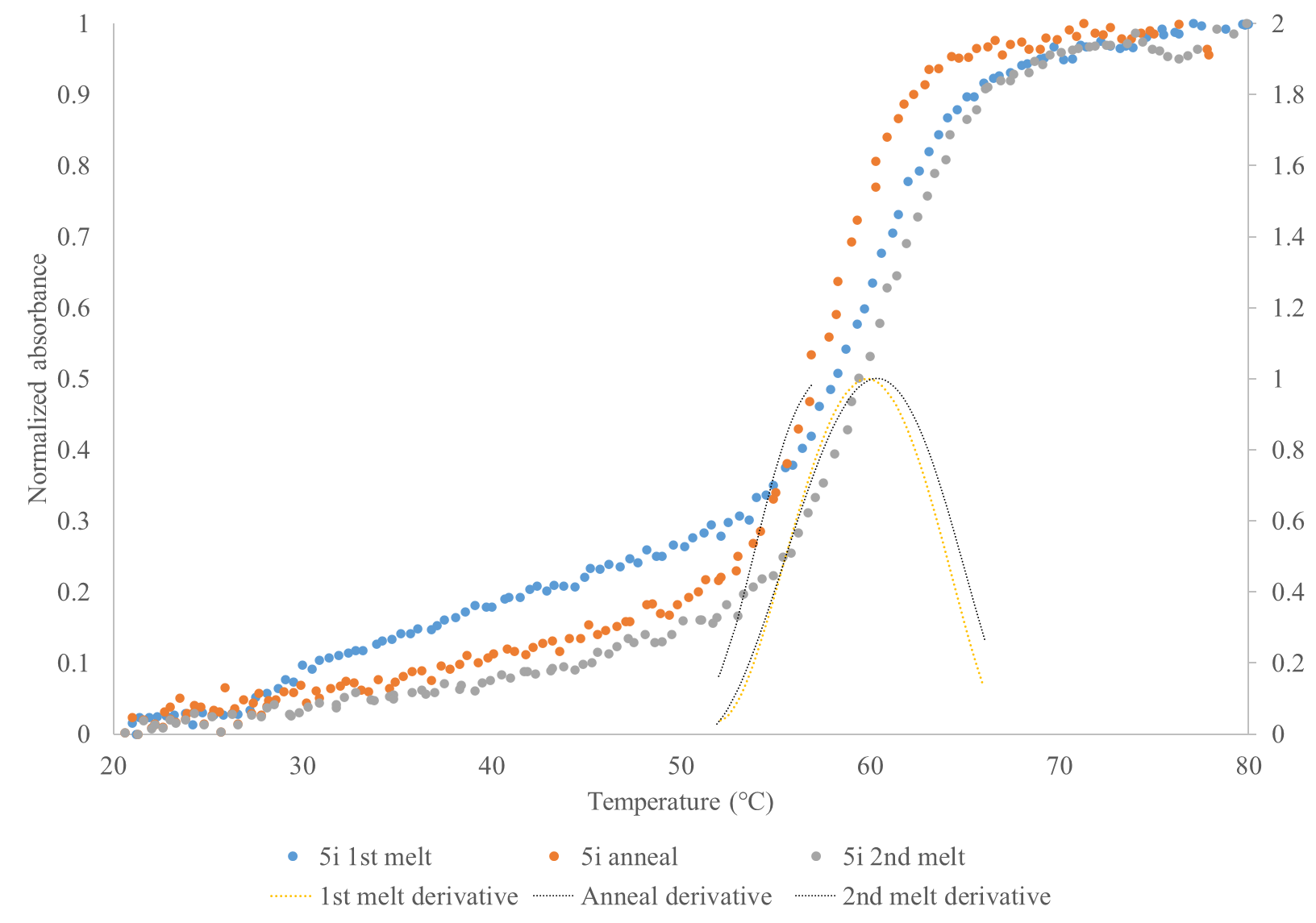


**Figure S10.** DNA melting curve of **5i** and the complementary strand.


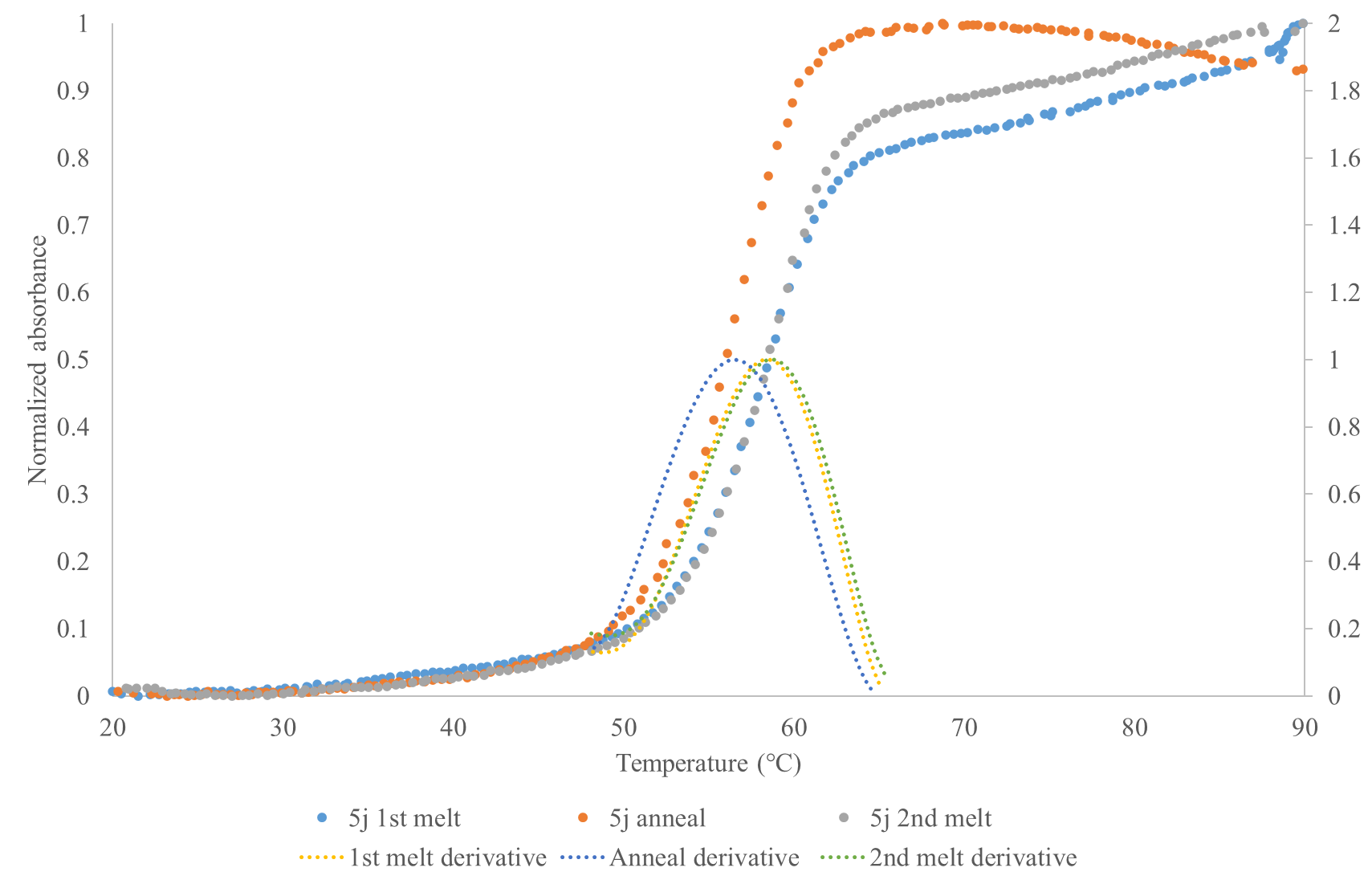


**Figure S11.** DNA melting curve of **5j** and the complementary strand.


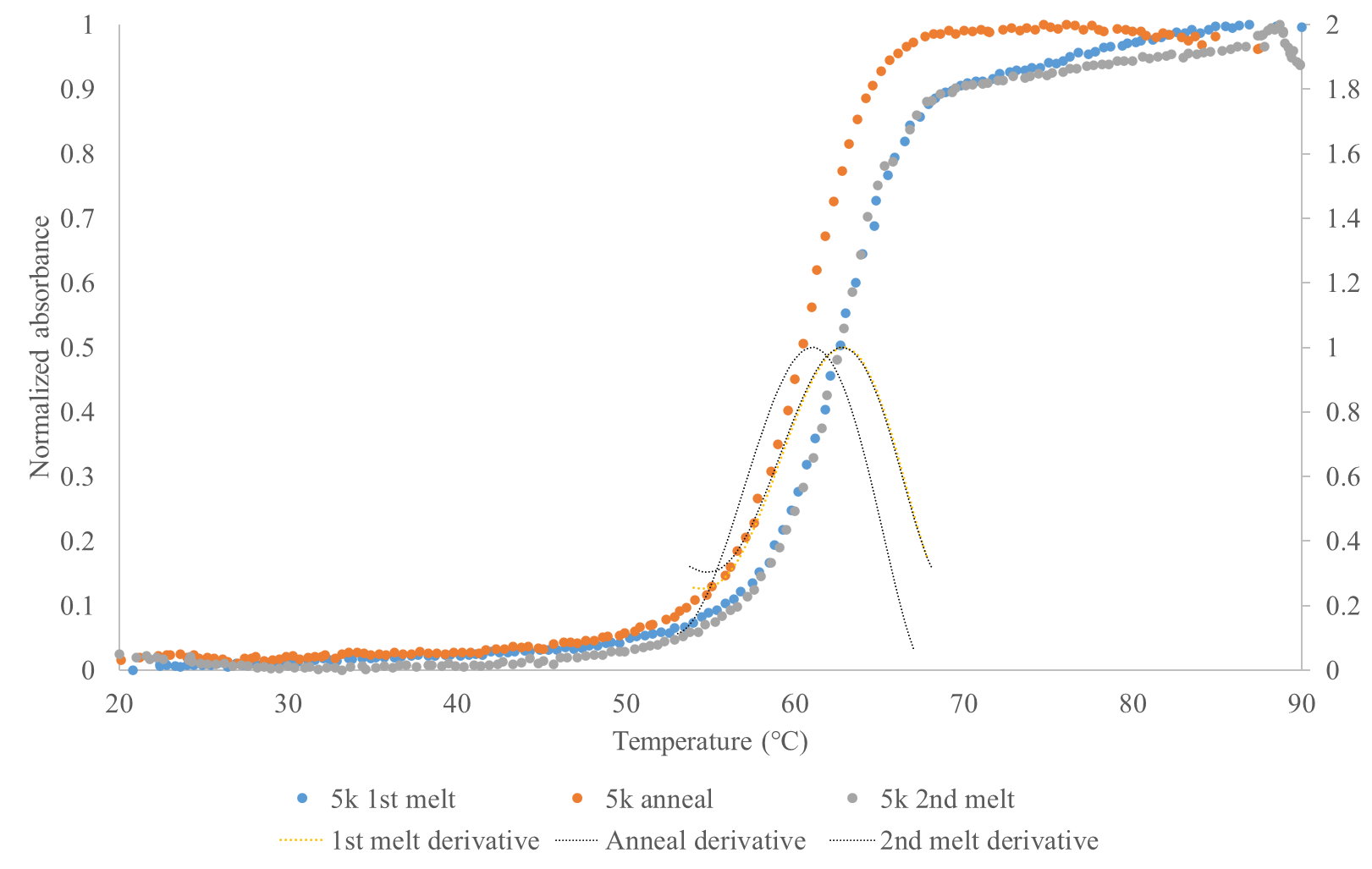


**Figure S12.** DNA melting curve of **5k** and the complementary strand.


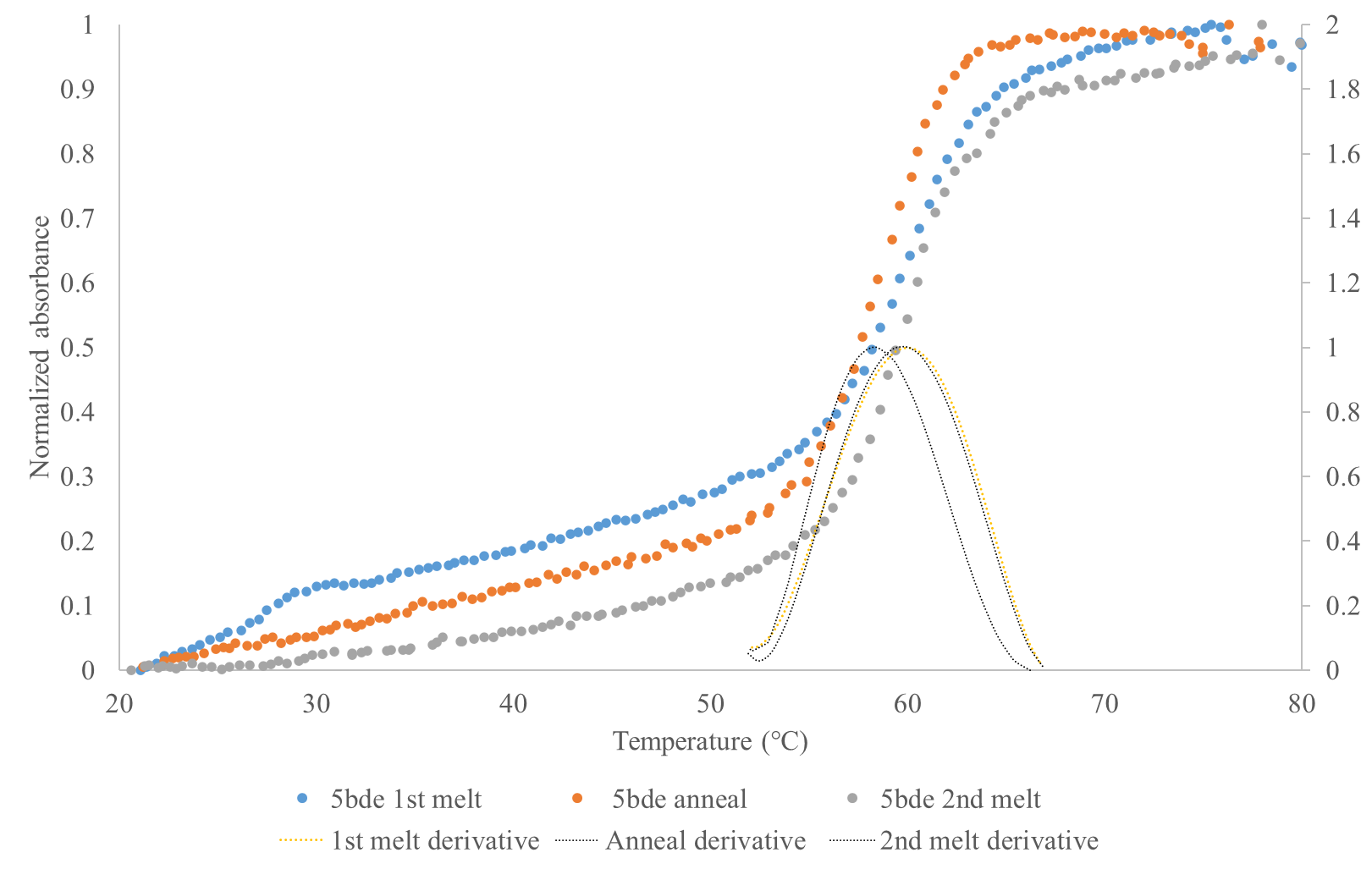


**Figure S13.** DNA melting curve of **5bde** and the complementary strand.

*Azobenzene hairpin (****8k4****) cis/trans DNA melting curves*


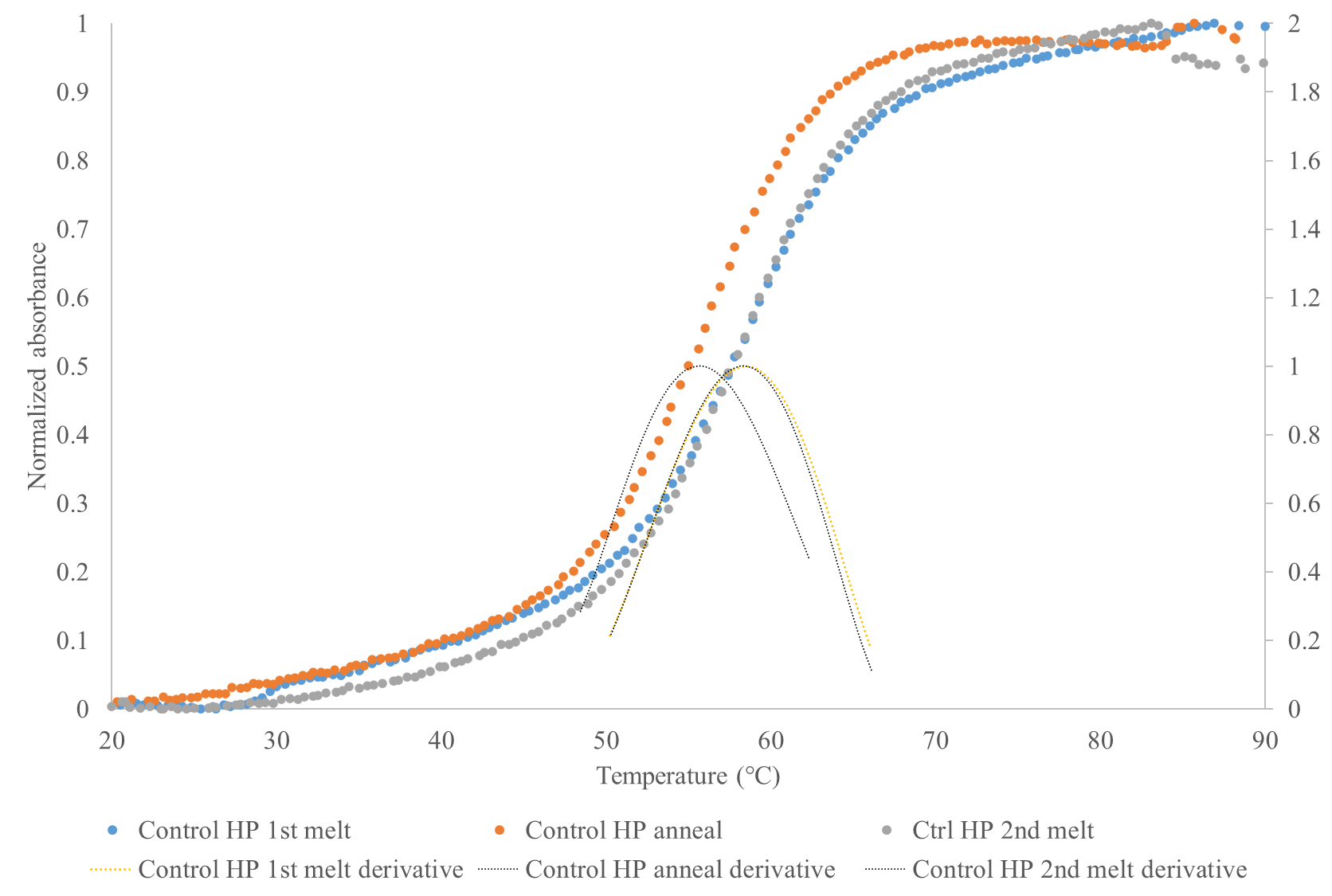


**Figure S14:** DNA melting curve of the non-modified control hairpin


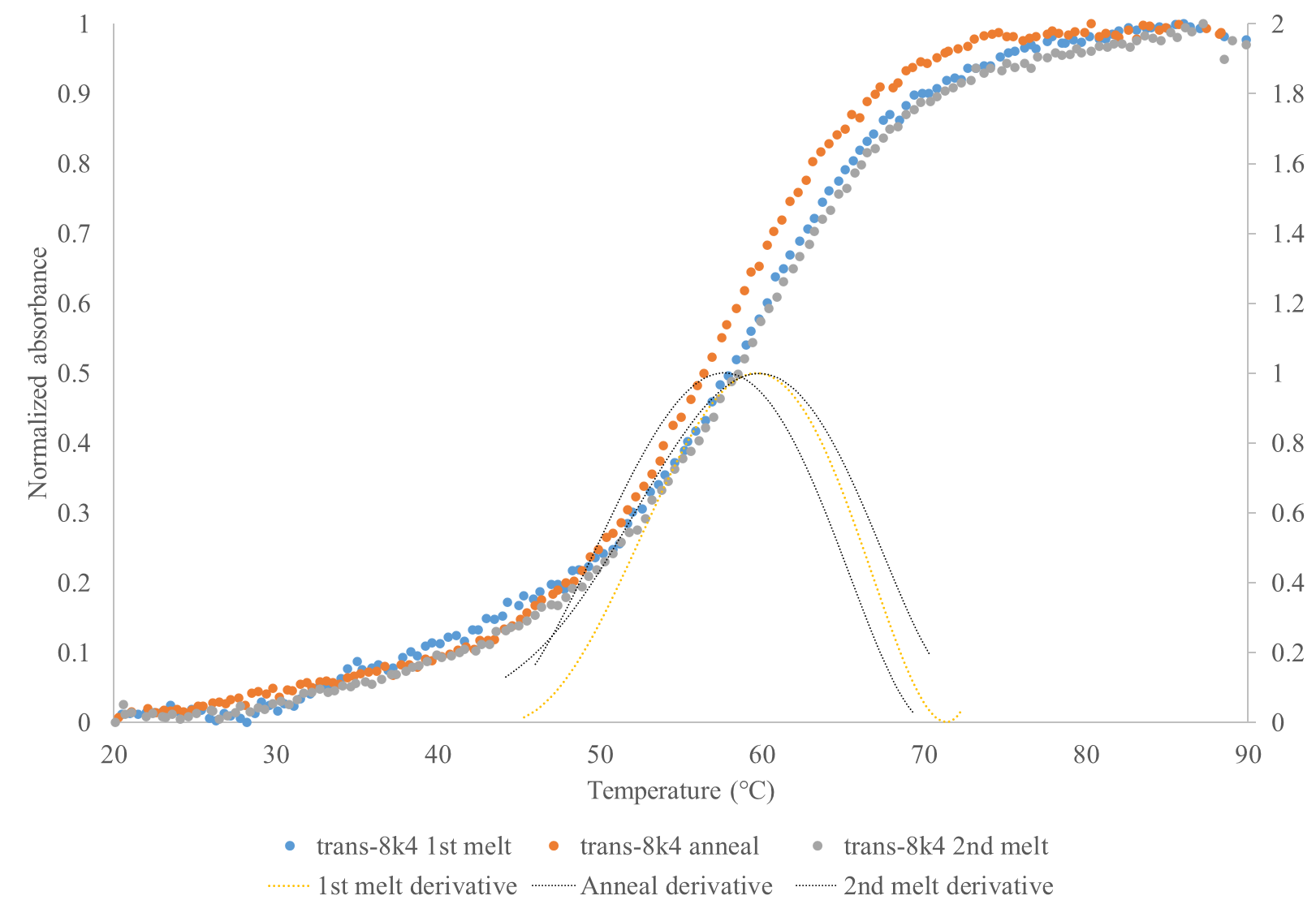


**Figure S15:** DNA melting curve of *trans*-**8k4** (non-irradiated)


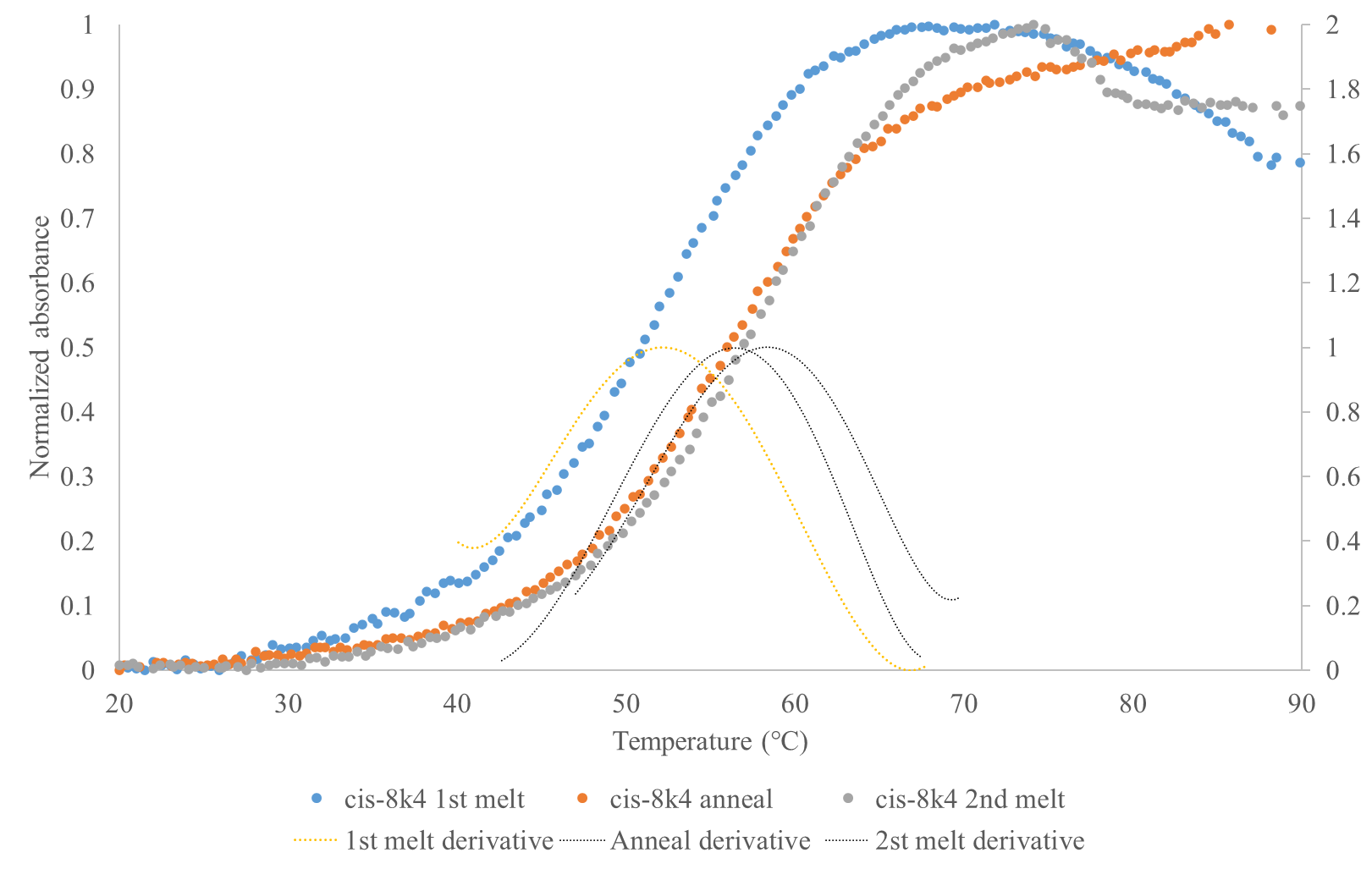


**Figure S16:** DNA melting curve of *cis*-**8k4** (irradiated). After the first melting curve, the annealing temperature and the second melting temperature increase significantly and are close to the ones measured in the *trans*-configuration. This indicates a thermal relaxation process of the *cis*-azobenzene group upon heating.


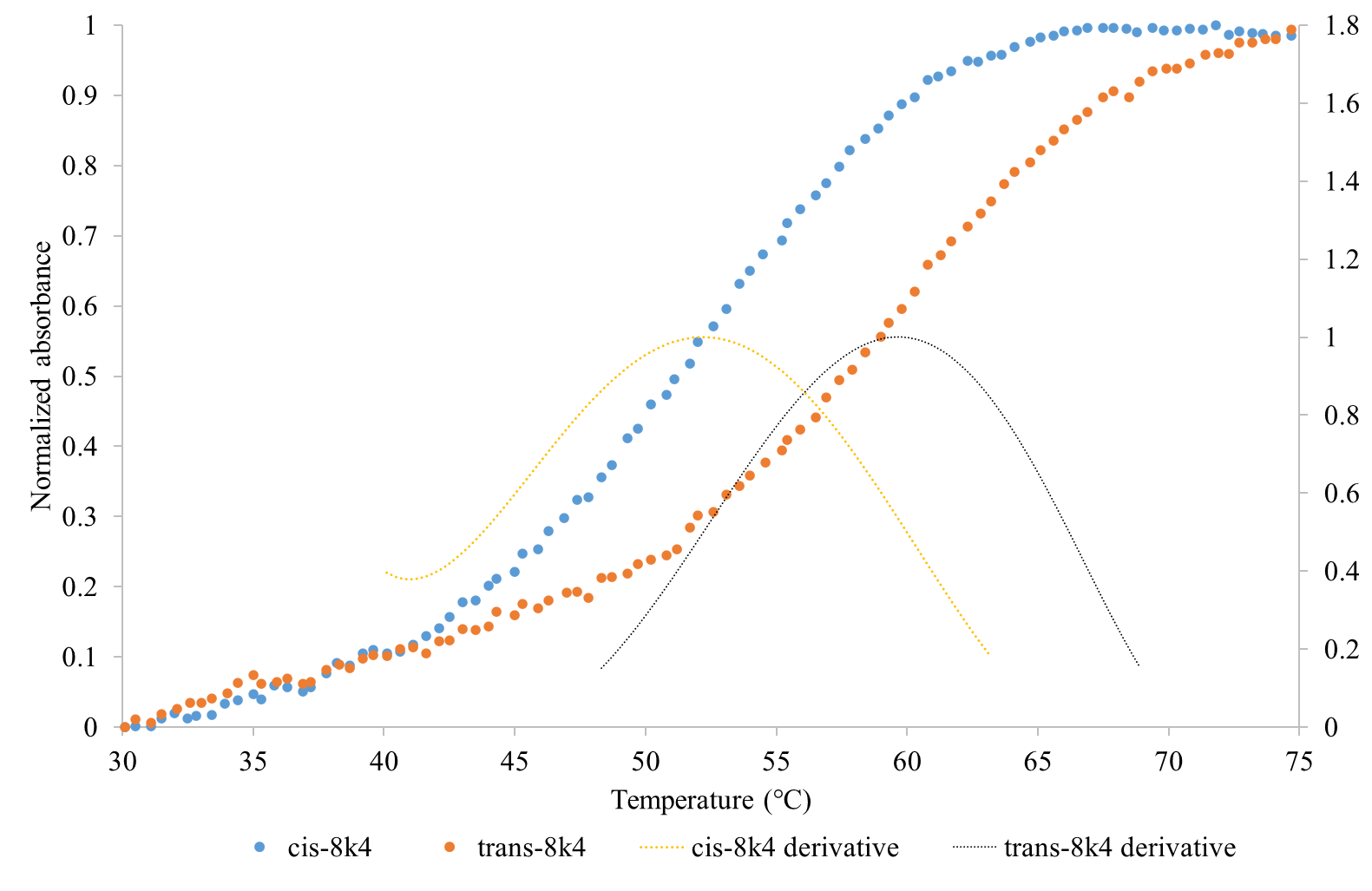


**Figure S17:** Juxtaposition of the first *cis*-**8k4** and *trans*-**8k4** melting curves.

**Table S1.** Melting temperatures of the irradiated and non-irradiated azobenzene modified hairpin (**8k4**) and its control unmodified strand.

| Hairpin strand | 1^st^ T_m_ (°C) | 2^nd^ T_m_ (°C) | | T_annealing_ (°C) |
| --- | --- | --- | --- | --- |
| Control | 58.4 | 58.4 | 55.6 | |
| trans-8K4 (non-irradiated) | 59.8 | 59.9 | 57.8 | |
| cis-8K4 (irradiated) | 52 | 58.5 | 56.4 | |

**Bioconjugation Assays**

The yield of the different reactions was determined via RP-HPLC of the crude mixture. The isolated products characterization was done via LCMS.

*Amino modified strand, NHS-chemistry assay* ***5a/b***

A mixture of **5a/b** (20 µM) and NHS-biotin (1 mM, 50 equivalents) in HEPES (100 mM, pH 8.2), using water as the solvent was done. The reaction was left at rt overnight.


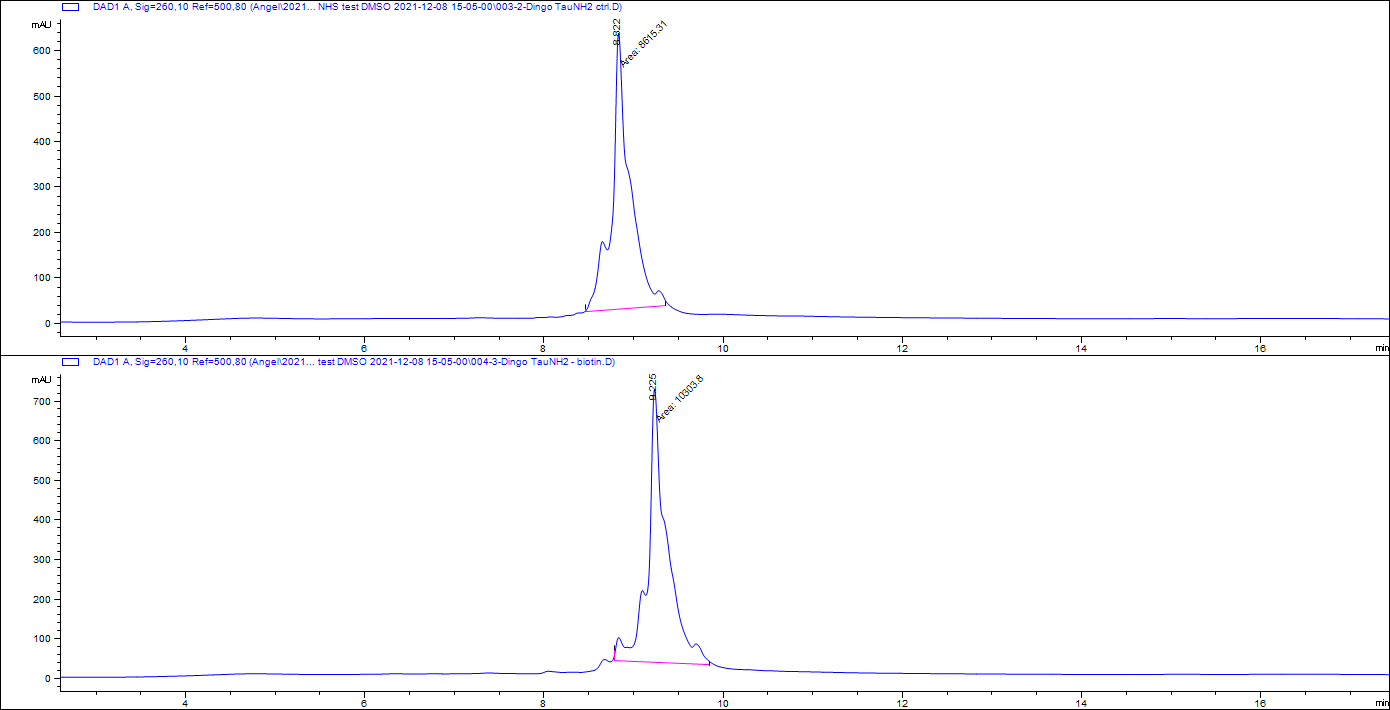


**Figure S18:** Juxtaposition of the **9a/b** reaction. Top chromatogram corresponds to **5a**, bottom chromatogram corresponds to **9a**. The reaction goes to full conversion.


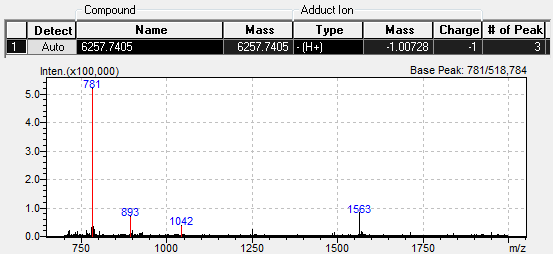


MS and mass deconvolution of strand **9a/b**

*Amino modified strand, NHS-chemistry assay* ***5c***

A mixture of **5c** (20 µM) and NHS-biotin (1 mM, 50 equivalents) in HEPES (100 mM, pH 8.2), using a 1:1 DMSO:water mixture as the solvent was done. The reaction was left at rt overnight.


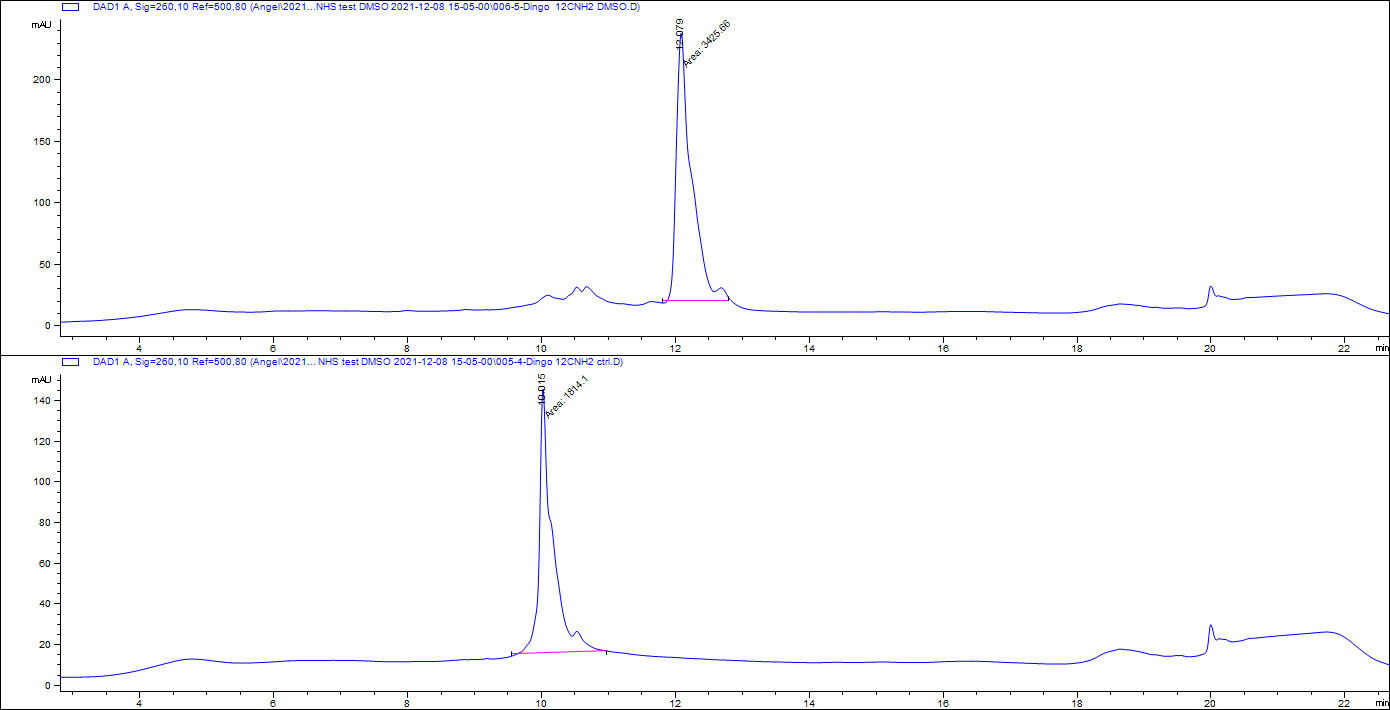


**Figure S19:** Juxtaposition of the **9c** reaction. Top chromatogram corresponds to **9c**, bottom chromatogram corresponds to **5c**. The reaction goes to full conversion.


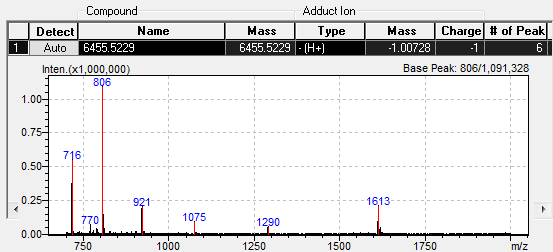


MS and mass deconvolution of strand **9c**

*Azide modified strand, strain-promoted azide-alkyne cycloaddition chemistry assay*

A mixture of **5d** (20 µM) and DBCO-PEG_4_-5/6-FAM (80 µM, 4 equivalents) in TEAA buffer (100 mM, pH 7), using water as the solvent was done. The reaction was left at rt overnight.


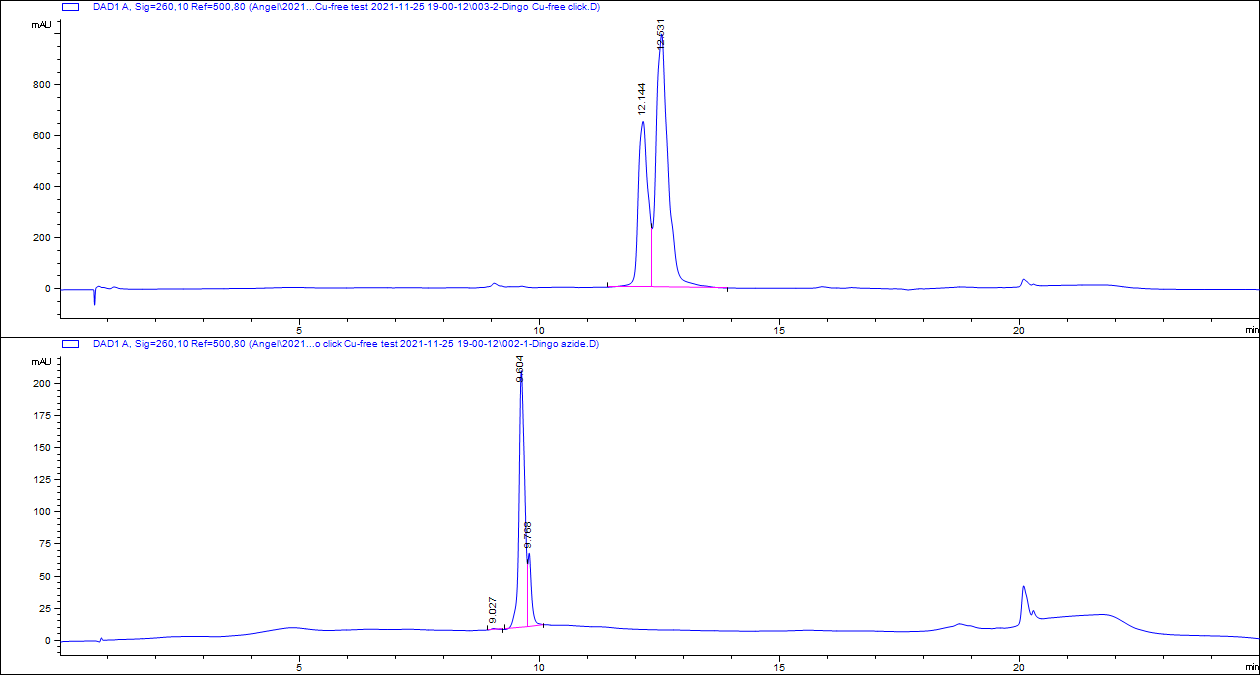


**Figure S20:** Juxtaposition of the **9d** reaction. Top chromatogram corresponds to **9d**, bottom chromatogram corresponds to **5d**. The reaction goes to full conversion.


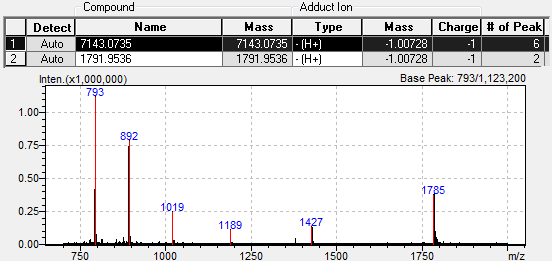


MS and mass deconvolution of strand **9d**

*Alkyne modified strand, copper catalyzed azide-alkyne cycloaddition chemistry assay*

A mixture of **5e** (20 µM), azide-PEG_3_-biotin (100 µM, 5 equivalents), ascorbic acid (0.5 mM), CuTBTA (0.5 mM), MgCl_2_ (10 mM) in a phosphate buffer (100 mM, pH 7.4), using water as the solvent was done. The reaction was left at 40 °C overnight.


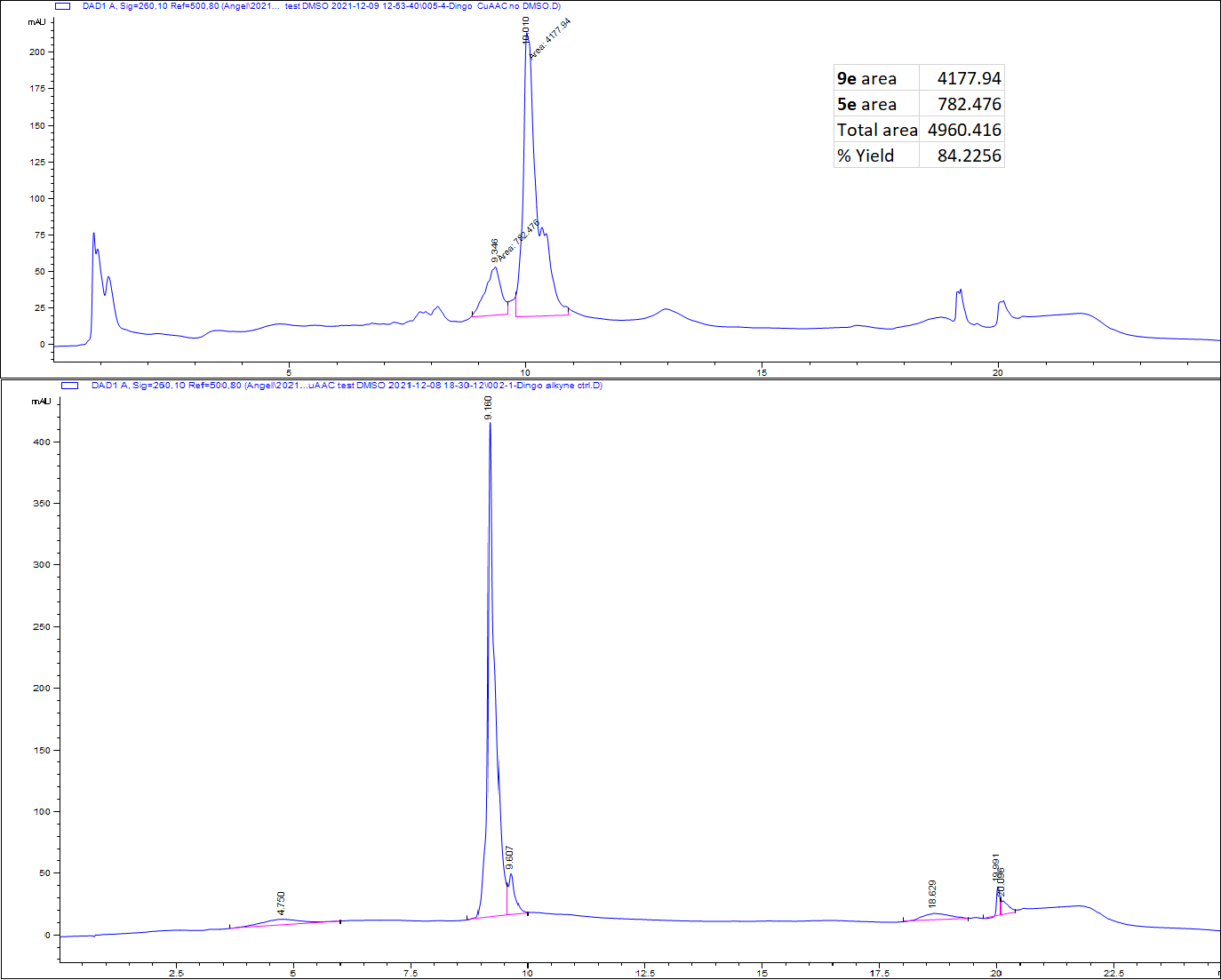


**Figure S21:** Juxtaposition of the **9e** reaction. Top chromatogram corresponds to **9e**, bottom chromatogram corresponds to **5e**. The reaction yield is calculated to be 84 %.


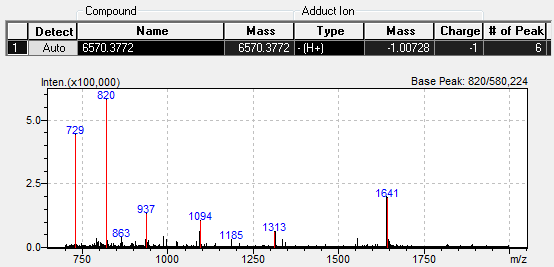


MS and mass deconvolution of strand **9e**

*Thiol modified strand, maleimide chemistry assay*

A mixture of 5f (20 µM), maleimide-biotin (400 µM, 20 equivalents) and TCEP (1 mM, 50 equivalents) in TEAA buffer (100 mM, pH 7), using water as the solvent was done. The reaction was left at rt overnight.


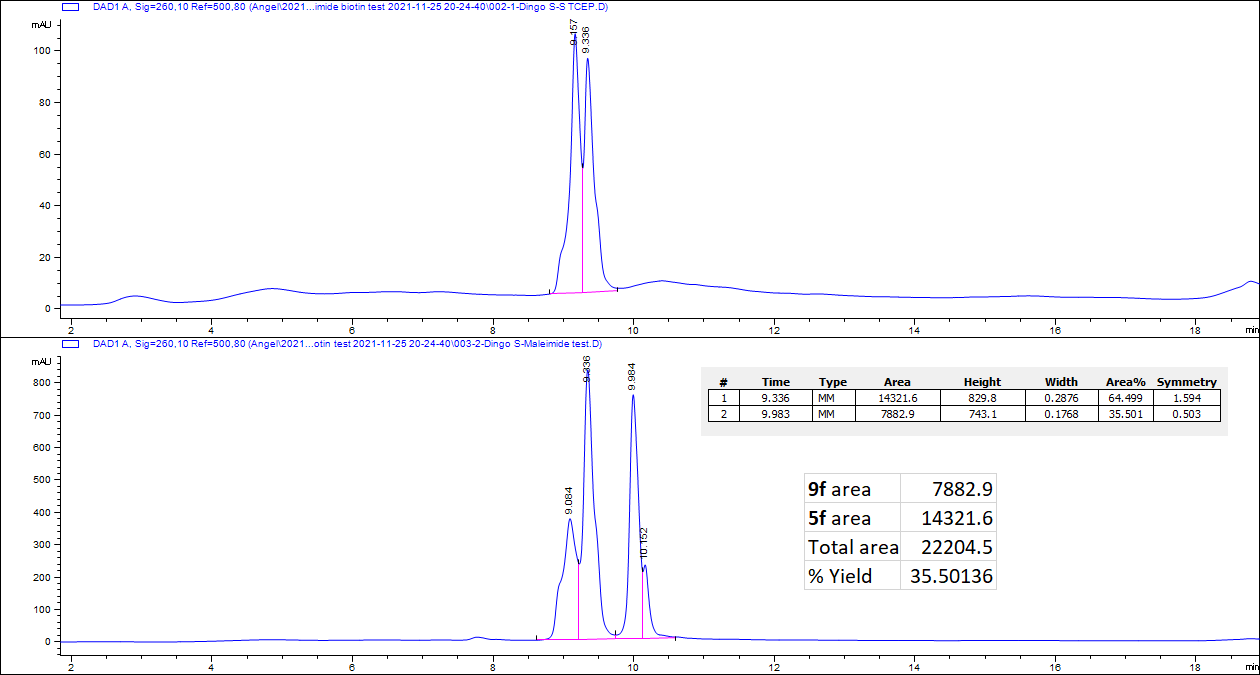


**Figure S22:** Juxtaposition of the **9f** reaction. Top chromatogram corresponds to **5f**, bottom chromatogram corresponds to **9f**. The reaction yield is calculated to be 36 %.


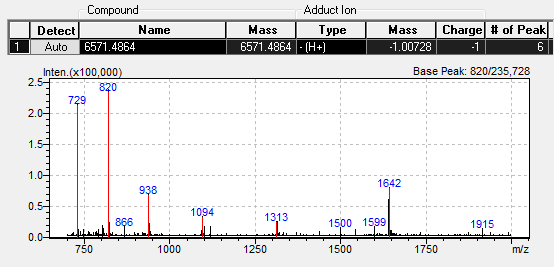


MS and mass deconvolution of strand **9f**

**References**

1. Brouwer,A., Monnee,M. and Liskamp,R. (2000) An Efficient Synthesis of N-Protected β-Aminoethanesulfonyl Chlorides: Versatile Building Blocks for the Synthesis of Oligopeptidosulfonamides. Synthesis, 2000, 1579-1584.
2. Liskamp,R.M., Brouwer,A.J., Merkx,R., Dabrowska,K., Rijkers,D.T. (2006) Synthesis and Applications of β-Aminoethanesulfonyl Azides. Synthesis, 2006, 455-460
3. Clavé,G., Dorsum,E., Vasseur,J.-J., Smietana,M. (2020) An Entry of the Chemoselective Sulfo-Click Reaction into the Sphere of Nucleic Acids. Org. Lett., 22, 1914-1918
4. Novopashina,D., Sinyakov,A., Ryabinin,V., Perrouault,L., Giovannangeli,C., Venyaminova,A. and Boutorine,A. (2013) Oligo(2′-O-methylribonucleotides) and their derivatives: IV. Conjugates of oligo(2′-O-methylribonucleotides) with minor groove binders and intercalators: Synthesis, properties, and application. Russ. J. Bioorg. Chem., 39, 138-152.
